# Bay-Region Functionalisation of Ar-BIAN Ligands and Their Use Within Highly Absorptive Cationic Iridium(III) Dyes

**DOI:** 10.1038/s41598-017-14996-4

**Published:** 2017-11-14

**Authors:** Kamrul Hasan, Jingyi Wang, Amlan K. Pal, Claus Hierlinger, Véronique Guerchais, Han Sen Soo, Felipe García, Eli Zysman-Colman

**Affiliations:** 1 Département de Chimie, Université de Sherbrooke, 2500 Boul. de l’Université, Sherbrooke, QC J1K 2R1 Canada; 2 Department of Chemistry, College of Sciences, University of Sharjah, Sharjah, P. O. Box 27272 UAE; 3 Division of Chemistry and Biological Chemistry, School of Physical and Mathematical Sciences, Nanyang Technological University, 21 Nanyang Link, Singapore, 637371 Singapore; 4 Organic Semiconductor Centre, EaStCHEM School of Chemistry, University of St Andrews, St Andrews, Fife KY16 9ST UK; 5 Institut des Sciences Chimiques de Rennes, UMR 6226 CNRS-Université de Rennes 1, Campus de Beaulieu, 35042 Rennes Cedex, France; 6 Solar Fuels Laboratory, Nanyang Technological University, 50 Nanyang Avenue, Singapore, 639798 Singapore

## Abstract

We report the synthesis, UV-vis absorption, electrochemical characterisation, and DFT studies of five panchromatic, heteroleptic iridium complexes (four of which are new) supported by Ar-BIAN ligands. In particular, the synthesis of an ester-functionalised Ar-BIAN ligand was carried out by a mechanochemical milling approach, which was advantageous over conventional metal templating solution methods in terms of reaction time and product purity. The introduction of ester and carboxylate functionalities at the bay region of the acenaphthene motif increases each ligand’s π-accepting capacity and imparts grafting capabilities to the iridium complexes. These complexes have absorption profiles that surpass the renowned **N3** dye [Ru(dcbpy)_2_(NCS)_2_] (dcbpy = 4,4′-dicarboxy-2,2′-bipyridine), making them of interest for solar-energy-harvesting applications.

## Introduction

There is growing recognition of the need for alternative sources of energy to reduce our reliance on fossil fuels and overcome the challenges of global climate change and environmental pollution. Solar energy is one of the sustainable alternatives, and several promising approaches to use sunlight include the generation of electricity with photovoltaics, the production of solar fuels and chemicals by artificial photosynthesis, and the storage of solar thermal energy^1^. One of the established technologies that can be exploited for both photovoltaics and artificial photosynthesis is the dye-sensitised solar cell (DSSC)^2^, which has lately been adapted for the production of solar chemicals in a dye-sensitised photoelectrosynthesis cell (DSPEC)^3–6^. The workhorse behind both the DSSCs and the DSPECs are the photosensitisers that absorb UV to NIR radiation^2–6^, but notably, the dyes predominantly comprise heteroleptic ruthenium complexes (*e*.*g*. the celebrated **N3 dye**) or complicated porphyrin systems^7,8^. On the contrary, iridium-based photosensitisers have received greater attention in the nascent field of photoredox catalysis for organic syntheses^9–13^. Iridium complexes have potential benefits of higher thermal and chemical stability, similar quantum yields, and longer-lived excited states in comparison to ruthenium compounds^14^, and iridium systems have been successfully deployed in numerous light harvesting and emitting applications^15–19^. Despite this, there has been a dearth of reports on iridium-based photosensitisers in DSSCs or DSPECs^14^.

Our team has sought to expand the library of potential candidates for light harvesting by developing iridium and other metal complexes composed of bis(arylimino)acenaphthene (Ar-BIAN) ligands that are highly modular and can be readily synthesised from commercially available reagents^20–24^. The Ar-BIANs are known to be versatile π-acceptors and have been employed for hydroamination and hydrogenation catalysis with iridium complexes^25–27^. We surmised that the Ar-BIAN ligand can be functionalised with an electron-withdrawing anchoring functionality such as a carboxylate, with the dual function of facilitating grafting on the surface of metal oxides, and also enhancing the absorption profile. Herein, we describe the synthesis of the first Ar-BIAN ligand possessing methyl carboxylate and carboxylic acid substituents in the bay region of the acenaphthene motif *via* a mechanochemical milling approach, in which reactions are induced by mechanical energy through ball milling and grinding^28^.

**Figure 1:**
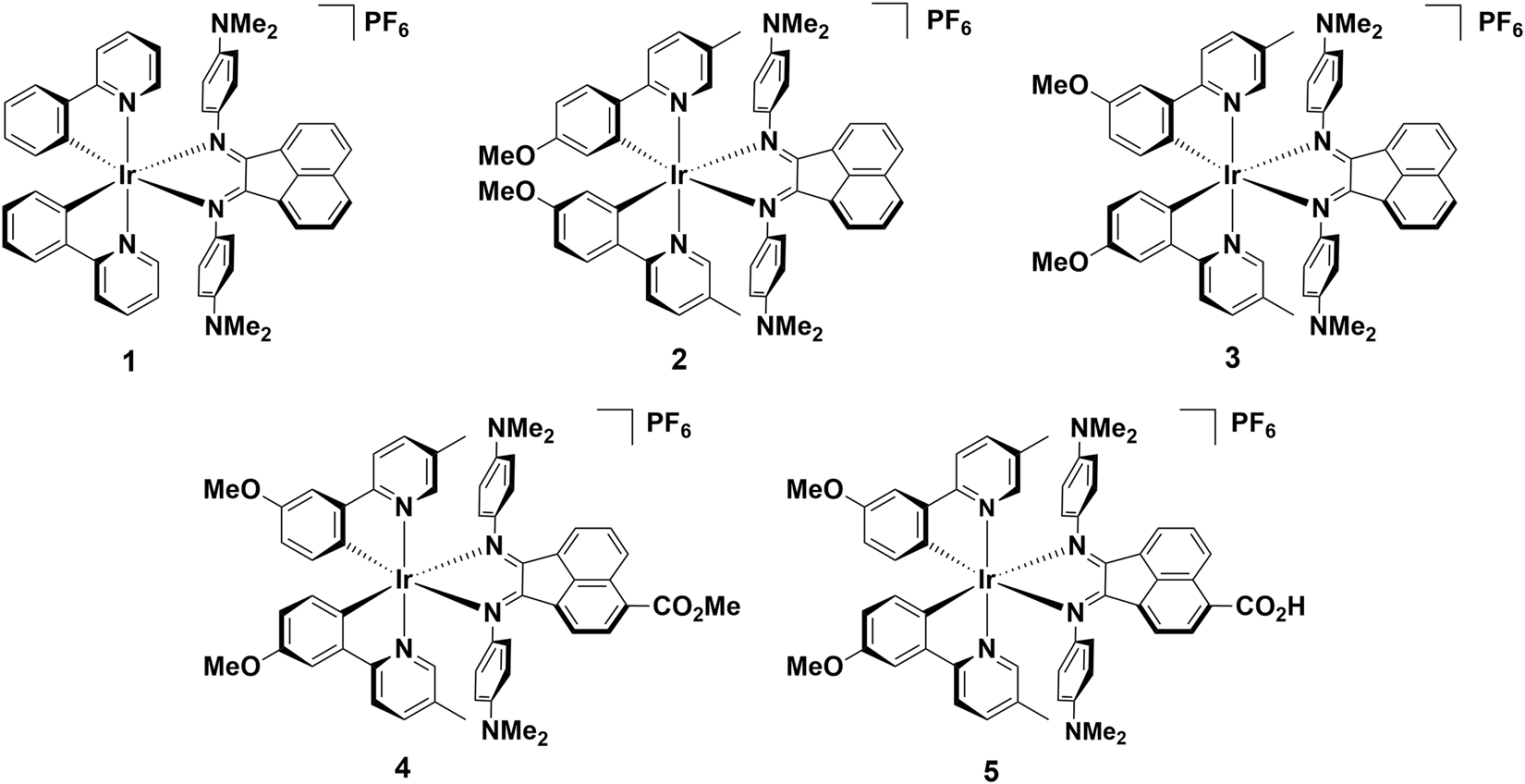
Complexes investigated in this study.

Although solution-based methods have traditionally been utilised for chemical synthesis by default, there has been increasing interest in recent years towards mechanochemical synthesis, partly because it has been shown to reduce reaction times and can sometimes provide almost quantitative yields^29^. A specific mechanochemical technique is ball milling, in which reaction vessels are oscillated from side-to-side^30^. This motion creates impact between the ball-bearing inside the reaction vessel, the chemical contents, and the walls of the vessel, providing energy input to drive chemical reactions. The speed and milling time, together with the size of the ball bearing in the reaction vessel are adjustable and can be systematically varied^30^. Mechanochemical synthesis through ball milling is a more eco-friendly, essentially solvent-free approach that has been exceptionally effective to access metastable compounds and materials that may react with coordinating solvents^31^. We present optical absorption spectroscopy, electrochemical measurements, and density functional theory (DFT) calculations of a series of five complexes (Fig. 1) to highlight how we have achieved panchromatic light absorption with modest effects on the electrochemical potentials, indicating that the carboxylate on the Ar-BIAN ligand can potentially be employed in DSSCs and DSPECs.

## Results and Discussion

**Figure 2:**
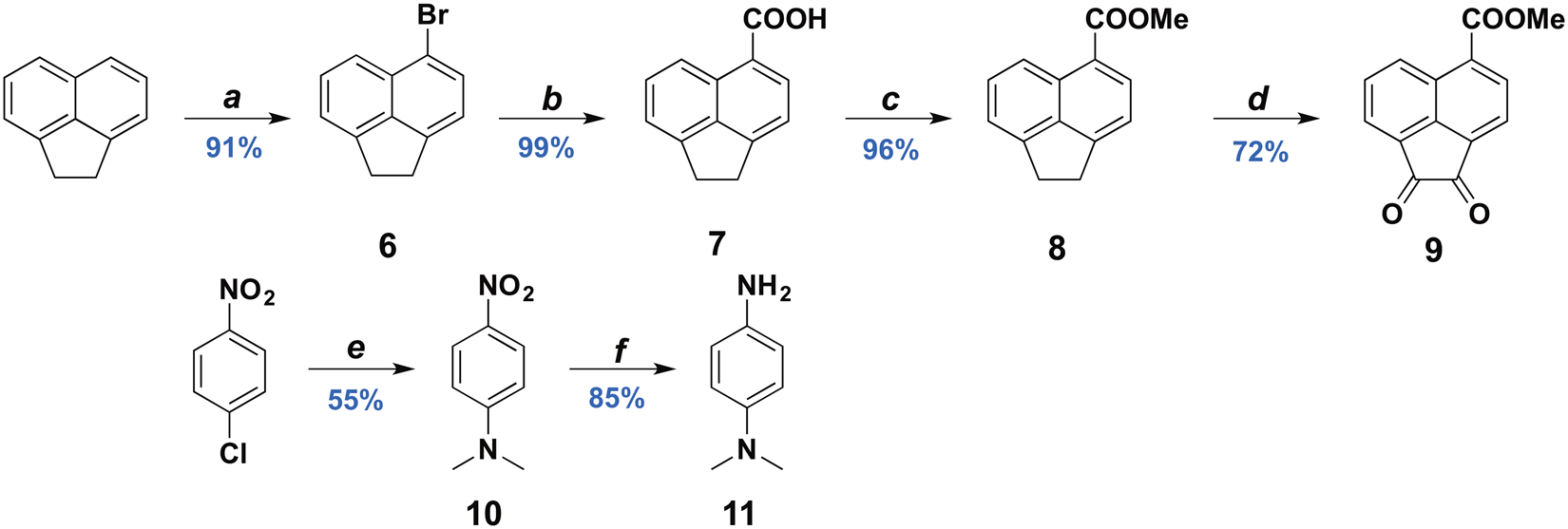
Synthesis of 5-carboxymethylacenaphthoquinone, **9**, and *N*,*N*-dimethyl-4-phenylenediamine, **11**. (**a**) 1 equiv. NBS/MeCN, RT, 19 h; (**b**) i. 1.5 equiv. *n*-BuLi, −78 °C 30 min; ii. CO_2_(s), RT; (**c**). 2.5 equiv. SOCl_2_/MeOH, reflux, 19 h; (**d**) i. 7.8 equiv. CrO_3_/Ac_2_O, 110 °C; ii. conc. HCl, 0 °C; (**e**) 10 equiv. KOH/DMF, 155 °C, 19 h; (**f**) 3 equiv. NaBH_4_, 20 mol% Cu(acac)_2_/1:1 v/v *i*-PrOH:EtOH, 35 °C, N_2_, 9 h.

**Figure 3:**
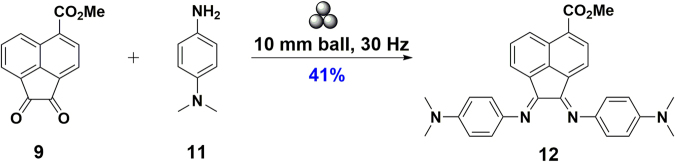
Mechanochemical synthesis of a methyl ester-modified Ar-BIAN ligand. The symbol for mechanical milling above the arrow of the equation has been proposed by Hanusa *et al*.^31^.

The synthesis of 5-carboxymethylacenaphthoquinone, **9**, and *N*,*N*-dimethyl-4-phenylenediamine, **11**, is outlined in Fig. 2. Although compounds **6**, **10**, and **11** are commercially available, we elected to synthesise them from much more affordable precursors. Acenaphthene was regioselectively monobrominated at the 5-position using *N*-bromosuccinimide in acetonitrile (MeCN)^32^. The vital carboxymethyl group was installed in two steps. Lithiation of **6** and quenching with dry ice^33^ yielded **7**, following which **7** was esterified using thionyl chloride and methanol to afford **8** in excellent yield^34^. Oxidation of **8** with chromium trioxide in acetic anhydride following the procedure of Pei and co-workers^35^ resulted in the formation of an inseparable mixture of dione **9** and a second similarly symmetric product, putatively identified as the enediol by ^1^H NMR spectroscopy. Initially, we attempted to prepare ligand **12** *via* solution methods through a ZnCl_2_ templated condensation between **9** and **11** ^36^, the latter of which was obtained in two steps from 4-chloronitrobenzene *via* nucleophilic aromatic substitution with DMF^37^, followed by reduction of the nitro group to the corresponding amine^38^. This templating method with ZnCl_2_ was deemed necessary because acid-catalysed condensation reactions do not work well with sterically hindered ketones and amines. Furthermore, Cenini *et al*. showed that the driving force for this double condensation is the precipitation of the metal Ar-BIAN complex. In cases where the final metal complex did not precipitate out, the product was formed in only minute amounts^39^. However, in the present case, attempts at removal of the Zn salts often led to hydrolysis, hindering purification of the ligand. Instead, ligand **12** was successfully obtained by mechanochemical synthesis through an acetic acid-catalysed condensation between **9** and **11** (Fig. 3). Mechanochemical synthesis through ball milling has been found to be remarkably effective for the solid-state synthesis of both organic and inorganic molecules, including an indium Ar-BIAN complex from our team, and has been especially advantageous for the facile synthesis and purification of **12** here^23^.

**Figure 4:**
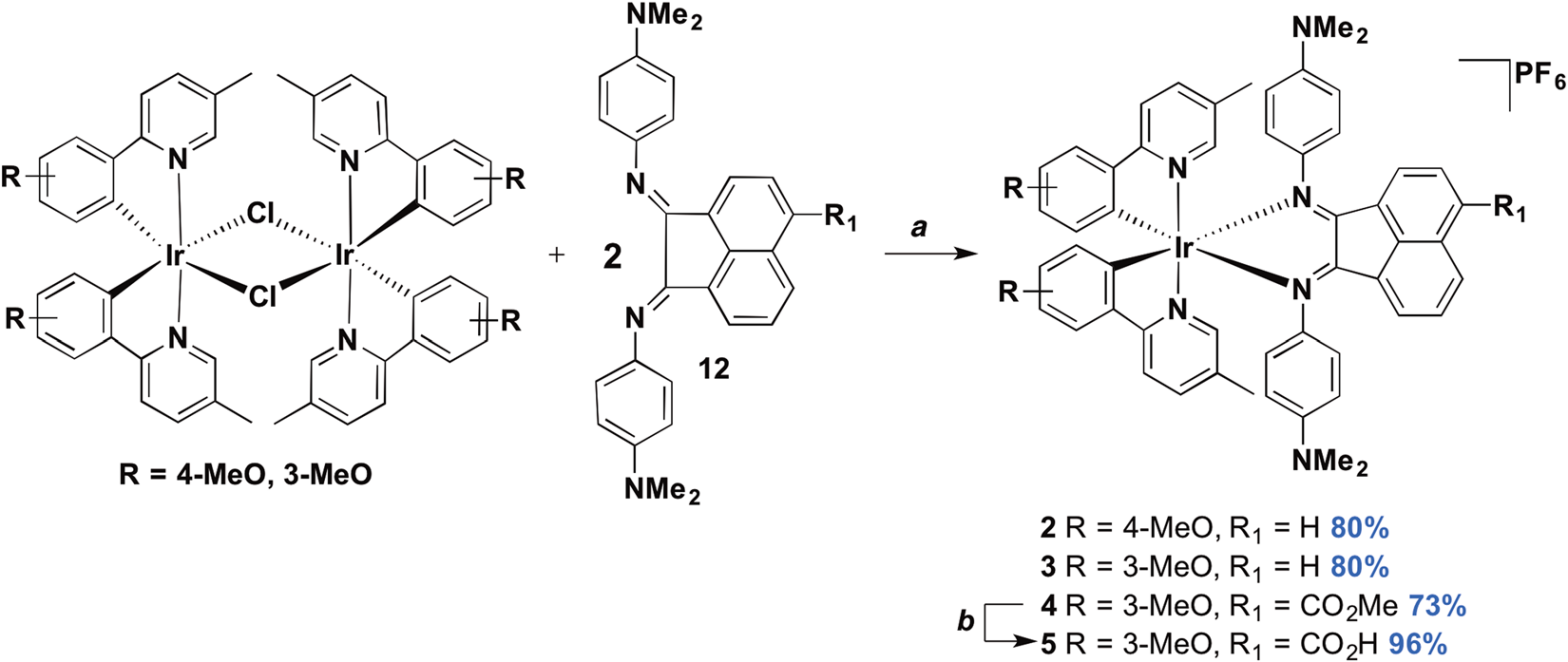
Synthesis of iridium(III) complexes **2**–**5**. (**a**) i. DCM, 50 °C, 19 h; ii. NH_4_PF_6_ (aq); (**b**) 2.7 equiv. NaOH/1:1 MeCN:H_2_O v/v, 85 °C, 22 h.

In parallel, 2-(3′-anisyl)-5-methylpyridine (3MeO-5Meppy) and 2-(4′-anisyl)-5-methylpyridine (4MeO-5Meppy) were obtained in 47 and 95% yield, respectively, through the Suzuki coupling of 2-bromo-5-methylpyridine and the corresponding arylboronic acids^36,40^. The corresponding chloro-bridged iridium(III) dimers were then isolated in 68 and 71% yield, respectively, following the procedure of Watts and co-workers^41^. We had previously reported **1** ^20^, a reference compound for our current manuscript, and we adopted a similar protocol to synthesise mononuclear complexes **2**–**4**. The new Ir complexes were prepared in good yields through cleavage of the corresponding iridium dimer, [Ir(C^N)_2_(μ-Cl)]_2_, with the corresponding Ar-BIAN ligands (Fig. 4). Saponification of the methyl ester in **4** afforded complex **5** almost quantitatively. The identity and purity of **2**–**5** were confirmed by ^1^H and ^13^C NMR spectroscopy, melting points, and high-resolution mass spectrometric (HRMS) analyses.

**Figure 5:**
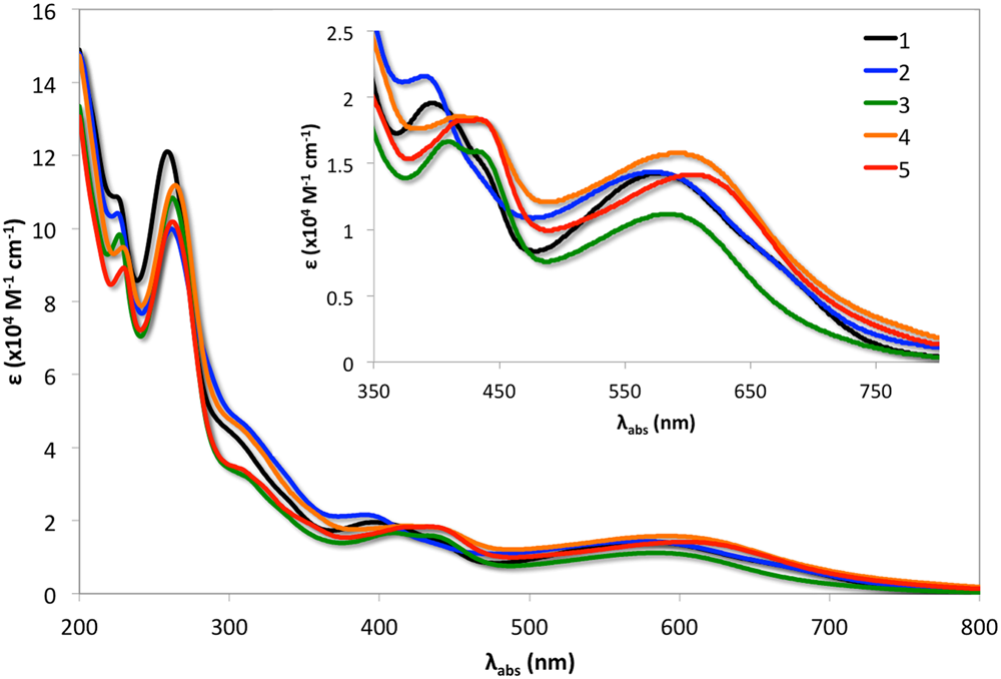
Electronic absorption spectra of **1**–**5** recorded in MeCN at 298 K. Inset: Expanded UV-vis-NIR absorption spectra from 350–800 nm.

**Figure 6:**
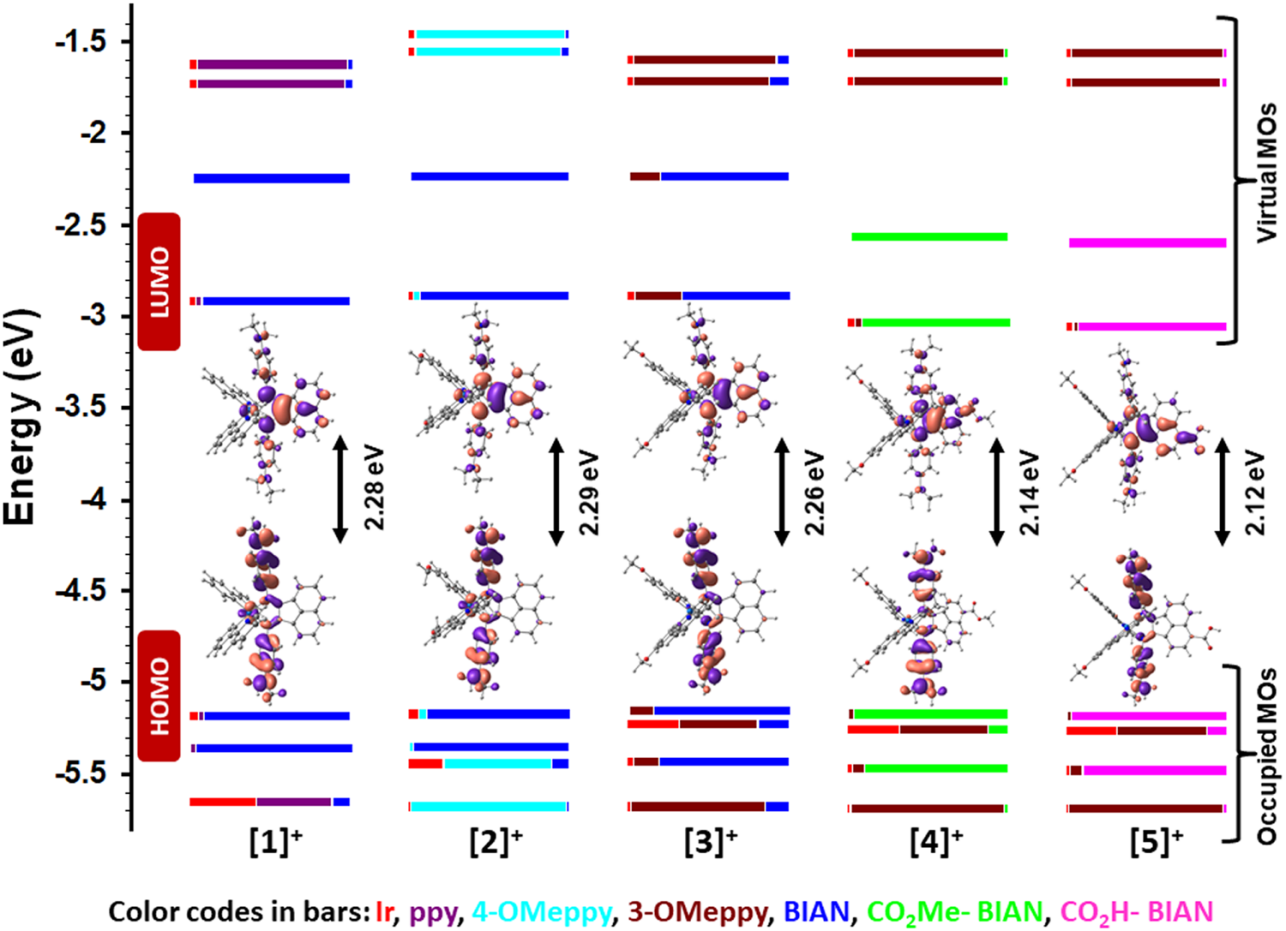
Calculated frontier Kohn-Sham MOs of **1**–**5**. DFT calculations were performed with the B3LYP/SBKJC-VDZ basis set for Ir(III) and 6–31 G** for C, H, N, and O, using a CPCM (MeCN) solvent model. The orbitals are isocontoured at 0.02.

To assess the light absorption behavior among our iridium(III) complexes, the UV-vis-NIR absorption spectra were collected in acetonitrile (MeCN) solution and are illustrated in Fig. 5. Some of the significant absorption band maxima along with their corresponding molar extinction coefficients are collected in Table 1. Overlays of experimentally observed and theoretically predicted absorption data by TD-DFT are shown in Supplementary Figs S23–S27. In all of these complexes, the HOMO resides on the *N*,*N*-dimethylaniline part of the Ar-BIAN ligand and the LUMO resides on the acenaphthylene-1,2-diimine core of the Ar-BIAN ligand (Fig. 6). The intense bands in the UV region at ~225 nm are assigned to be spin-allowed ligand-centered (^1^LC) ^1^(π-π*) transitions^37^. For **1**, the band at 258 nm is an admixture of ^1^LC, (^1^(π_Ar-BIAN_-π_Ar-BIAN_*)) and singlet ligand-ligand charge transfer (^1^LLCT) (Ar-BIAN(π) to ppy(π*)) transitions (Supplementary Table S1). For complexes **2**–**5**, the bands at ~260 nm consist primarily of ^1^LLCT (Ar-BIAN(π)to ppy(π*) for **2**) or ^1^LC (^1^(π_ppy_-π_ppy_*) for **3**–**5**), with minor contributions from singlet metal-to-ligand charge-transfer (^1^MLCT) (Ir(dπ) to ppy(π*) for **2**–**5**) (Supplementary Tables S1–S5). Similarly, the bands at ~310 nm of **1–5** also possess varying contributions of ^1^LC, ^1^LLCT, and ^1^MLCT transitions (Supplementary Tables S1–S5). Despite the structural variation on both sets of ligands in **1–5**, the energies of these higher-energy absorption bands differ very little across the series.

**Table 1:** Spectroscopic data for **1**–**5**.

Complex	λ_abs_ / nm (ε / 10^4^ M^−1^ cm^−1^)^a^
**1**	224 (10.9); 258 (12.1); 310 (sh,^b^ 4.02); 396 (1.95); 576 (1.42); 675 (sh,^b^ 0.71)
**2**	225 (10.4); 261 (9.99); 310 (sh,^b^ 4.62); 390 (2.15); 570 (1.43); 665 (sh,^b^ 0.78)
**3**	227 (9.82); 262 (10.8); 312 (sh,^b^ 3.18); 409 (1.66); 432 (1.59); 585 (1.11)
**4**	229 (9.48); 264 (11.2); 309 (sh,^b^ 4.48); 416 (1.85); 590 (1.57)
**5**	229 (8.91); 262 (10.2); 310 (sh,^b^ 3.37); 427 (1.82); 604 (1.41)

^a^Absorption spectra recorded in aerated MeCN at 298 K. Absorbance values were collected over a concentration range of 8.78 × 10^−2^ to 3.51 × 10^1^ µM, and the molar extinction coefficients (ε) were determined by assuming the complexes obeyed the Beer-Lambert law. ^b^Shoulder.

Inspection of the lower energy region of the spectrum reveals a more notable structure-property relationship. The absorption bands in **1** and **2** between 390–396 and 570–576 nm are shifted bathochromically in **3**–**5** to 409–427 nm and 585–604 nm, respectively. For **1**–**3**, the set of bands at 390–409 nm are composed of an admixture of ^1^LLCT (ppy(π) to Ar-BIAN(π*)) and ^1^MLCT (Ir((dπ)) to Ar-BIAN(π*)) transitions, whereas bands in **4** and **5** at 416 nm and 427 nm, respectively, are described primarily by ^1^LC (ppy(π) to ppy(π*)) transitions with minor contributions from ^1^MLCT (Ir((dπ)) to ppy(π*)) transitions (Supplementary Tables S1–S5). The magnitude of the red-shift for the absorption bands around 590 nm increases from **3** to **5**, while the bands centered around 400 nm are less sensitive to the functionalisation in the bay region of the acenaphthene core. The bands at 570–576 nm in **1** and **2** are principally ^1^LLCT (ppy(π) to Ar-BIAN (π*)) in nature with minor contribution from ^1^MLCT (Ir(dπ) to Ar-BIAN(π*)) (Supplementary Tables S1 and S2). For **3**, the band at 585 nm is an admixture of transitions, primarily consisting of ^1^LC (Ar-BIAN/ppy(π) to Ar-BIAN/ppy(π*)) with minor ^1^LLCT (Ar-BIAN(π) to ppy(π*)) contributions (Supplementary Table S3). On the other hand, notably, the bands at 590 nm and 604 nm in **4** and **5**, respectively, are composed exclusively of intra-ligand charge-transfer transitions (^1^ILCT) within the Ar-BIAN moiety (Supplementary Tables S4 and S5). Moreover, as the conjugative framework of the ancillary ligand is expanded with the incorporation of the carboxy moiety in **4** and **5**, the molar extinction coefficients of the charge-transfer (CT) bands also increase.

The absorption bands at λ_abs_ > 600 nm in **1** and **2** are principally ^1^ILCT and ^1^LLCT in character, respectively (Ar-BIAN(π) to Ar-BIAN(π*) for **1** and ppy(π) to Ar-BIAN(π*) for **2**). Although no distinct bands at λ_abs_ > 600 nm could be observed for **3**–**5**, spin-forbidden ^3^CT transitions at 752–783 nm with very low molar absorptivity are predicted by TD-DFT. Quantification of the light-harvesting capacities of **1**–**5** and comparison to that of the benchmark dye **N3**, [Ru(dcbpy)_2_(NCS)_2_] consisted of an analysis of the integrated product of their absorption spectra with the AM 1.5 solar irradiance spectrum over the range of 400–800 nm (dcbpy = 4,4′-dicarboxy-2,2′-bipyridine). Complexes **1**, **3**, and **5** absorb 1.56, 1.47, and 2.19 times more light over this spectral range compared to **N3**. The light harvesting capacity of **5** is in fact even larger since its absorption profile extends beyond 800 nm.

**Figure 7:**
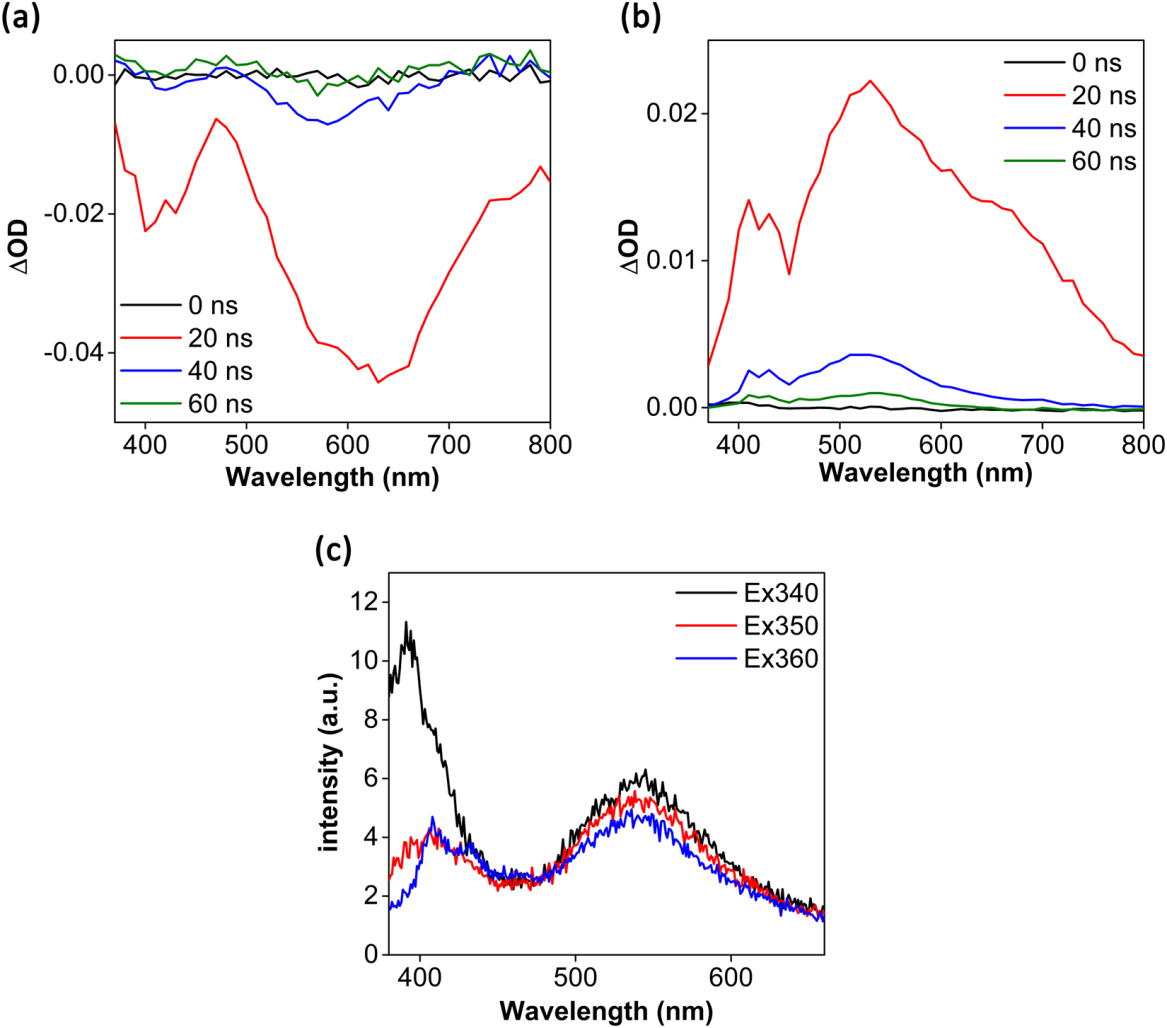
Transient (**a**) absorption and (**b**) emission spectra of 0.050 mM solutions of **4** collected in DCM at 298 K. (**c**) Steady-state photoluminescence spectra of a 0.050 mM solution of **4** collected in DCM at 298 K.

**Figure 8:**
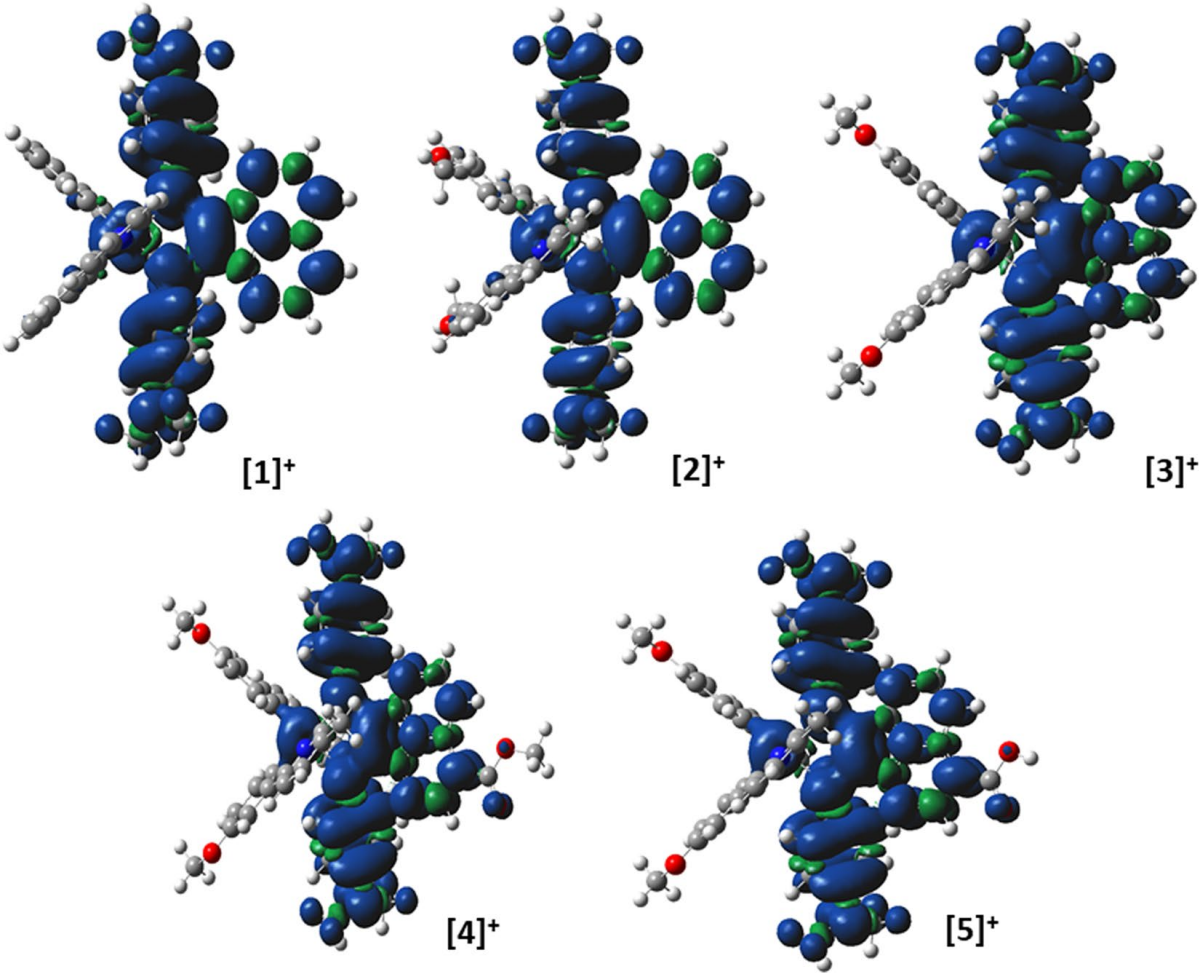
Triplet spin density distributions of complexes **1–5**, obtained from DFT calculations with the UB3LYP/SBKJC-VDZ basis set for Ir(III) and 6–31 G** for C, H, N, and O, using a CPCM (MeCN) solvent model. The contours are isovalued at 0.004.

In order to examine the time-resolved photophysical properties of the Ir complexes, steady-state photoluminescence (PL) spectra were first recorded for **4** (in DCM) and **5** (in MeCN since it is insoluble in DCM). For compound **4**, weak emission was observed at 540 and 410 nm when the sample was excited at 420 and 340 nm, respectively (Fig. 7). A similar emission profile was also observed when **5** was irradiated at the same wavelengths (Supplementary Fig. S28). We propose that the two emission bands may arise from mixed MLCT/ILCT transitions due to the two different ligand motifs. Both the C^N and Ar-BIAN ligands are π-acceptors, although the π* orbitals of the Ar-BIAN ligand are lower in energy due to more extensive conjugation. Thus, we expect that there could be MLCT/ILCT/LLCT excited states arising from promotion of the Ir *d* electrons to the two distinct ligand π* orbitals, resulting in radiative recombination from the Ar-BIAN ligand back to Ir, between the C^N and Ar- BIAN ligands, and within the Ar-BIAN ligand at a lower energy. The spin density distribution obtained from unrestricted DFT calculations are predominantly distributed within the Ar-BIAN ligand, with minor contributions from the Ir center (Fig. 8). The overall photoluminescence quantum yield, ϕ_PL_, for **4** was estimated to be 0.03% in DCM, using the comparative method described by Williams *et al*.^42^.

To obtain additional insights into the excited state characteristics of these Ir complexes, nanosecond transient absorption and emission spectroscopic measurements were conducted (Fig. 7). In each time resolved optical spectroscopic experiment, the sample was probed by a broadband xenon lamp beam before and after 5–8 ns pulses. In the transient absorption spectra, the detected intensity of the transmitted signal is presented as *∆OD*, which is the logarithm of the ratio of the light intensity from the probe beam after laser excitation to the intensity before laser excitation. Hence, a positive *∆OD* refers to increased absorption while a negative *∆OD* refers to reduced absorption/emission of the excited state relative to the ground state. The transient emission spectra show two broad emission bands with maxima at 410 nm and 530 nm when irradiated by a 355 nm laser pulse, matching the spectral profile derived from the steady-state PL experiments. On the other hand, the transient absorption spectra reveal two bands with negative *∆OD* peaked at 410 and 630 nm, which results from a superposition of both the excited state emission and the ground state bleach, since a fraction of the molecules has been promoted to the excited state by the laser pulse. We attempted to estimate the excited [Ir]^+*^ lifetime at 520 nm (and other wavelengths), but the lifetime turned out to be shorter than the time-resolution of our instrument (Supplementary Fig. S29). Nevertheless, the steady-state and time-resolved spectroscopic studies confirmed that both **4** and **5** exhibit weak PL with time-scales consistent with fluorescence. There is overlap between the PL and the absorption spectra for both **4** and **5**, concurring with our previous assignment that the spin-allowed fluorescence arises from the ^1^ILCT/^1^MLCT/^1^LLCT absorptions at higher energy, whereas the lowest energy absorption bands are due to spin-forbidden CT transitions (λ_abs_ > 600 nm). As reported by Tkachenko *et al*., the MLCT excited state for complexes with Ar-BIAN ligands decays to an intra-molecular Ar-BIAN triplet state, which typically decays back to the ground state on the order of picoseconds^43^. This is likely due to the substantial spatial overlap of the HOMO and LUMO at the bis(arylimine) part of the ligand, which facilitates ultrafast recombination. Presumably, the π-accepting orbitals become more localised on the acenaphthene bay region, which provides better spatial separation of the electron from Ir after mixed MLCT/ILCT/LLCT transitions, thus leading to sufficiently long-lived, radiative singlet photoexcited states. There was little solvent dependence observed for **4** (Fig. 7 and Supplementary Fig. S30), suggesting that ligand dissociation or exciplex formation with MeCN did not occur upon photoexcitation. Gratifyingly too, these observations validate our attempts to improve the excited state photophysical properties, grafting abilities, and applicability of the Ar-BIAN Ir complexes in DSSCs and DSPECs.

**Figure 9:**
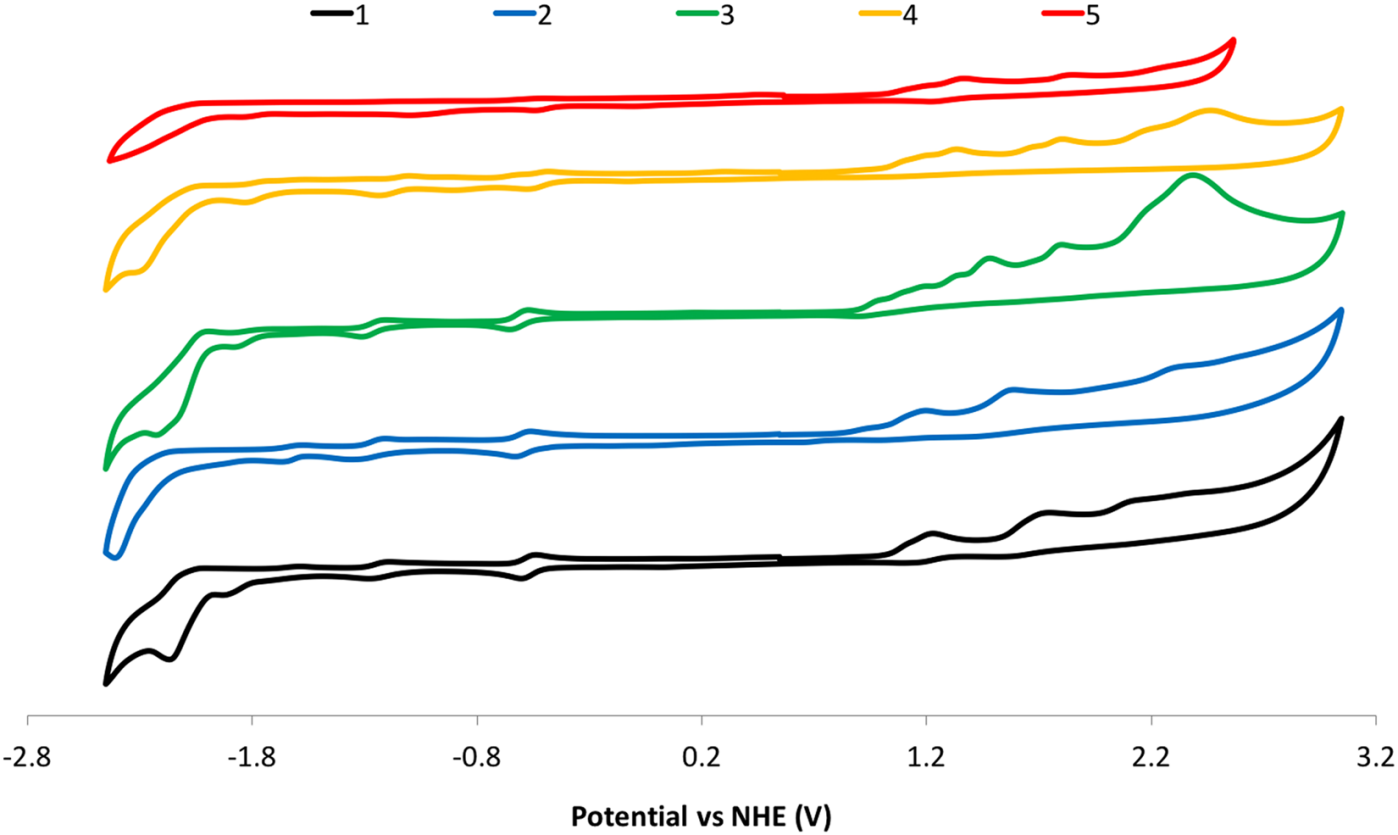
CV traces for **1**–**5** recorded at 298 K at 50 mV/s in MeCN solution with 0.10 M (n-Bu_4_N)PF_6_ as the supporting electrolyte.

Cyclic voltammetry studies of **1**–**5** were conducted in order to further probe their ground state electronic behavior. The cyclic voltammograms (CVs) are shown in Fig. 9 and the observed redox couples are summarised in Table 2. The CV of **1**, previously reported by us^20^, exhibits a reversible first reduction wave at −0.57 V versus NHE and a second quasi-reversible wave at −1.23 V, both ascribed to reduction of the Ar-BIAN ligand. The series of oxidation waves in **1** are all irreversible. We had previously assigned the first oxidation wave at 1.22 V to be localised on the *N*,*N*-dimethylaniline fragment, while the second oxidation consisted of contributions from the C^N ligands and the Ir^IV^/Ir^III^ redox couple. The incorporation of an electron-donating MeO group leads to a cathodic shift in both the reduction and oxidation waves^20,21,24,37^. The oxidation waves are more substantively affected with substitution at the 3-position resulting in a 0.13 V shift of *E* ^1^_ox_ to lower potential in **3** compared to a more modest 0.03 V shift in **2** with the 4MeO-5Meppy C^N ligand. The lower energies calculated for the HOMO of **2** (E_HOMO_ = −5.18 eV) compared to that of **3** (E_HOMO_ = −5.15 eV) are in good agreement with the higher anodic potentials measured for **2** compared to that of **3** (Table 2). Addition of the electron-withdrawing ester functionality in **4** results in an anodic shift of both the oxidation and the reduction waves. Hydrolysis of the ester to a carboxylic acid as in **5** anodically shifts *E* ^1^_ox_ further to 1.33 V, but does not dramatically affect *E* ^1^_red_. Thus, peripheral substitution of the iridium complexes in **2**–**5** does not appear to alter the nature of the first oxidation and reduction processes. In comparison, the important redox processes in the **N3** dye in methanol are found at 1.13 and −0.89 V, resulting in an electrochemical gap of 2.02 V, which is significantly larger than that found experimentally for **5** ^7^.

**Table 2:** Summary of electrochemical data for 1–5.

Compound	*E*_1/2_ /V vs NHE (Δ*E*_p_ /mV)^*a*^				ΔE^*d*^/V	E_HOMO_^*e*^/eV	E_LUMO_^*e*^/eV	${| {{\rm{\Delta }}E}_{{\rm{H}}-{\rm{L}}}| }^{{\rm{e}}}/{\rm{eV}}$	*E*_0,0_^*f*^/eV	*E* (S^+^/S^*^)^*g*^/V
	*E* ^1^_ox_	*E* ^2^_ox_	*E* ^1^_red_	*E* ^2^_red_						
**1**	1.22^*b*^	1.73^*b*^	−0.57 (70)^*c*^	−1.23 (60)^*b*^	1.82	−5.19	−2.91	2.28	1.66	−0.44
**2**	1.19^*b*^	1.57^*b*^	−0.60 (65)^*c*^	−1.28 (131)^*b*^	1.82	−5.18	−2.89	2.29	1.60	−0.41
**3**	1.09^*b*^	1.51^*b*^	−0.61 (75)^*c*^	−1.28 (65)^*b*^	1.74	−5.15	−2.89	2.26	1.66	−0.57
**4**	1.21^*b*^	1.38^*b*^	−0.53 (81)^*c*^	−1.17 (127)^*b*^	1.77	−5.18	−3.04	2.14	1.55	−0.34
**5**	1.33^*b*^	1.83^*b*^	−0.52 (78)^*b*^	−1.05 ^*h*^	1.89	−5.18	−3.06	2.12	1.55	−0.27

^*a*^CV traces recorded at 298 K at 50 mV/s in MeCN solution with 0.1 M (n-Bu_4_N)PF_6_. Values are in V vs. NHE (Fc^+^/Fc vs. NHE = 0.63 V). The numbers in parentheses refer to ΔE_p_, which is the difference between the anodic and cathodic peak potentials^38^. A non-aqueous Ag^+^/Ag electrode (silver wire in a solution of 0.1 M AgNO_3_ in MeCN) was used as the pseudo reference electrode; a glassy-carbon electrode was used for the working electrode, and a Pt electrode was used as the counter electrode. ^*b*^Irreversible. E_pa_ reported for oxidation peak potentials and E_pc_ for reduction peak potentials. ^*c*^Quasireversible. ^*d*^ΔE = ΔE_redox_ = E^1^_ox_(pa)-E^1^_red_(pc). Note that the E^1^_ox_(pa) and E^1^_red_(pc) are distinct from E_1ox_ and E_1red_, respectively, in the table since the latter are averaged values for the quasi-reversible redox waves. ^*e*^DFT calculations were performed with the B3LYP/SBKJC-VDZ basis set for Ir(III) and 6–31 G** for C, H, N, and O, using a CPCM (MeCN) solvent model. ^f^E_0,0_ is estimated from the onset of the absorption spectrum at ca. 10% intensity. ^*g*^Calculated from E(S^+^/S*) = E(S^+^/S) − E_0,0_. ^*h*^E^2^_red_ (pc).

## Conclusions

Overall, we have presented a series of five panchromatic iridium complexes (four of which are new) coordinated by Ar-BIAN ligands. The Ir complexes have absorption profiles that surpass the renowned **N3** dye, and both the electrochemical measurements and DFT calculations support the existence of MLCT, LLCT, and ILCT mixed states that account for the low energy optical absorption bands. Most significantly, in contrast to traditional solution methods, mechanochemical ball milling enabled the synthesis of new Ar-BIAN ligands in which ester and carboxylate functionalities are present at the bay region of the acenaphthene motif. This increases the ligands′ π-accepting capacities and imparts grafting capabilities to our iridium complexes. Our ongoing efforts include the introduction of these iridium Ar-BIAN compounds into DSSCs and more generally, the creation of new Ar-BIAN copper and other metal complexes that can be incorporated into DSPECs to produce solar fuels and chemicals.

## Methods

### Compound 5-carboxy-1,2-dihydroacenaphthene, 7

To a −78 °C (60 mL) solution of **6** (3.00 g, 12.8 mmol, 1.0 equiv.) in diethyl ether, 1.6 M *n*-BuLi (12 mL, 19 mmol, 1.5 equiv.) was added dropwise over 30 min. The reaction mixture was stirred an additional 30 min at −78 °C. The solution was then allowed to warm to RT and stirred for an additional 1 h. The reaction was quenched with dry ice and a white precipitate formed, which was separated by vacuum filtration to obtain the desired product. The crude product was purified by recrystallisation using aqueous ethanol, and collected as an off-white solid (2.51 g). **Yield:** 99%. **Mp:** 217 °C. ^**1**^ **H NMR (400 MHz, D**_**2**_ **O) δ (ppm):** 7.91 (d, *J* = 8.37 Hz, 1 H), 7.57 (d, *J* = 7.05 Hz, 1 H), 7.30 (t, *J* = 7.58 Hz, 1 H), 7.06 (t, *J* = 6.85 Hz, 2 H), 3.04 (s, 4 H). ^**13**^ **C NMR (100 MHz, D**_**2**_ **O) δ (ppm):** 171.4, 149.4, 146.7, 138.9, 130.9, 129.0, 128.8, 128.4, 121.2, 119.5, 118.6, 29.9, 29.7. **HRMS (EI, 70 eV):** [M-H]^−^ **Calculated:** (C_13_H_9_O_2_) 197.0597; **Found:** 197.0603. This compound has been twice previously reported where characterisation was limited to a melting point (Mp^44^: 207–211 °C and Mp^45^: 214–218 °C).

### Compound 5-carboxymethyl-1,2-dihydroacenaphthene, 8

To a stirred solution of **7** (3.00 g, 15.1 mmol, 1.0 equiv.) in 50 mL of MeOH cooled in an ice bath was added dropwise, SOCl_2_ (1.8 mL, 38 mmol, 2.5 equiv.) over 30 min. The reaction mixture was then allowed to warm to RT, before it was heated to reflux for 19 h. The MeOH was evaporated under reduced pressure and H_2_O was then added (50 mL). The product was extracted with DCM (50 mL), and the organic phase was dried over MgSO_4_, filtered under vacuum, and concentrated under reduced pressure to obtain the desired product as a brownish solid (2.45 g). **Yield:** 96%. **Mp:** 74 °C. ^**1**^ **H NMR (400 MHz, CDCl**_**3**_ **) δ (ppm):** 8.63 (d, *J* = 8.37 Hz, 1 H), 8.29 (d, *J* = 7.35 Hz, 1 H), 7.59 (dd, *J* = 6.91, 8.56 Hz, 1 H), 7.35 (d, *J* = 6.87 Hz, 1 H), 7.30 (dd, *J* = 1.25, 7.37 Hz, 1 H) 3.99 (s, 3 H), 3.42 (s, 4 H). ^**13**^ **C NMR (100 MHz, CDCl**_**3**_ **) δ (ppm):** 168.1, 153.2, 146.5, 139.8, 133.3, 130.4, 130.0, 122.2, 122.1, 120.2, 118.6, 52.03, 30.7, 30.6. The characterisation matches that previously reported^46,47^.

### Compound 5-methylcarboxylate-1,2-dioxo-1,2-dihydroace-naphthylene, 9

Compound **8** (1.63 g, 7.67 mmol. 1.0 equiv.) was dissolved in 50 mL of acetic anhydride at 110 °C. CrO_3_ (6.0 g, 60 mmol, 7.8 equiv.) was added carefully to the stirred solution over a period of 1 h. The resulting green suspension was stirred at 110 °C for a further 1 h and poured onto crushed ice. Concentrated HCl (10 mL) was added and the mixture was filtered. The yellow precipitate was washed with water and dried in vacuum. The crude product (*R*_*f*_ of 0.15, DCM on silica) was purified by column chromatography with silica gel using MeOH/DCM (2.5:97.5%). A yellow flaky solid (1.31 g) was collected. **Yield:** 72%. **Mp:** 189 °C. ^**1**^ **H NMR (400 MHz, CDCl**_**3**_ **) δ (ppm):** 9.32 (dd, *J* = 8.68, 19.9 Hz, 1 H), 8.58 (m, 2 H), 8.16 (dd, *J* = 7.16, 29.0 Hz, 1 H), 7.95 (m, 1 H), 4.08 (s, 3 H). ^**13**^ **C NMR (100 MHz, CDCl**_**3**_ **) δ (ppm):** 188.2, 187.4, 166.2, 145,9, 132.9, 132.5, 132.0, 131.2, 130.3, 129.7, 129.3, 122.8, 121.0, 53.0. HR-MS (EI, 70 eV): [M + Na]^+^ **Calculated:** (C_14_H_8_O_4_Na) 263.0315; **Found:** 263.0324.

### Compound (1E,2E)-methyl-1,2-bis((4-(dimethylamino)phen-yl)imino)-1,2-dihydroacenaph-thylene-5-carboxylate, 4-Me_2_N Ph-CO_2_MeBIAN, 12

A stainless steel grinder jar was dried in an oven at 120 °C overnight prior to use. The grinder jar was charged with **9** (0.072 g, 0.30 mmol), *N*,*N*-dimethyl-4-phenylenediamine (0.094 g, 0.67 mmol), acetic acid (4.3 µL, 0.075 mmol, 25 mol%), and Na_2_SO_4_ (0.043 g, 0.30 mmol), and equipped with a 10 mm stainless steel ball. The contents in the jar were then ground for 4 h at 30 Hz. The resultant purple gel was suspended in dichloromethane (DCM), filtered, and the filtrate was concentrated to dryness and rinsed with cyclohexane. The purple reside was recrystallised from diethyl ether (Et_2_O) and the isolated yield was 0.054 g (41%). ^**1**^ **H NMR (400 MHz, CDCl**_**3**_) ***δ*** **(ppm):** 8.90 (d, *J* = 8.4 Hz, 1 H), 8.16 (d, *J* = 7.6 Hz, 1 H), 7.51 (t, *J* = 8.0 Hz, 1 H), 7.32–7.35 (m, 2 H), 7.10–7.14 (m, 4 H), 6.82–6.86 (m, 4 H), 3.99 (s, 3 H), 3.04 (s, 6 H), 3.03 (s, 6 H). ^**13**^ **C{** ^**1**^ **H} NMR (100 MHz, CDCl**_**3**_ **)** ***δ*** **(ppm):** 166.9, 160.3, 159.7, 148.8, 148.5, 141.6, 141.5, 141.2, 133.3, 132.0, 130.0, 129.7, 129.3, 128.2, 127.2, 123.7, 121.9, 120.8, 120.5, 113.4, 113.1, 52.4, 41.1, 41.0. **HRMS (ESI + , m/z):** [M + H]^+^ **Calculated:** (C_30_H_29_N_4_O_2_) 477.2291; **Found** 477.2268. Anal. **Calcd**. for C_30_H_28_N_4_O_2_: C, 75.61; H, 5.92; N, 11.76; **Found:** C, 75.89; H, 5.57; N, 11.29.

### General procedure for the synthesis of Ir(III) bis[(C^N)-N,C^2′^]-*N*,*N’*-bis(phenylmino)acenaphthene (Ar-BIAN) hexafluoro-phosphate complexes 1–4

The iridium(III) dimer [(C^N)_2_Ir(*μ*-Cl)]_2_ (0.080 mmol, 1.0 equiv.) and bis(arylimino)acenaphthene (Ar-BIAN) ligand (0.16 mmol, 2.0 equiv.) were solubilised in 12 mL of dry DCM. The mixture was degassed repeatedly, placed under N_2_, and heated to 50 °C for 19 h. Over the course of the reaction, the mixture darkened in color from the initial yellow. The solution was cooled to RT and the solvent was removed under reduced pressure. The crude solid was re-dissolved in a minimum amount of MeOH and added slowly to an aqueous solution of NH_4_PF_6_ (10 mL, 6.13 mmol, 1 g/10 mL) under gentle stirring. The first drop caused the precipitation of a dark-colored solid. The solid suspension was conserved at 0 °C for 2 h, collected on a Buchner funnel, and the resulting solid was washed with water and Et_2_O. The residue was dried *in vacuo* to obtain a dark brown/green solid. The complexes were then crystallised in dichloromethane/diisopropylether (50:50) by slow evaporation. Complexes **1–4** were obtained using this protocol.

### Iridium(III)bis[2-phenyl-pyridinato-N,C^2′^]-*N*,*N’*-bis(4-*N*,*N*-di-methylphenylimino)acenaph-thenehexafluorophos-phate: [Ir(ppy)_2_(4-NMe_2_PhBIAN)]^+^(PF_6_)^−^, 1

Black crystals (0.146 g). Yield: 86%. The characterization matches that previously reported^21^.

Iridium (III) bis[4-methoxy-2-phenyl-5-methylpyridinato-N,C^2′^]-*N*,*N’*-bis(4-*N*,*N*-dimethyl-phenylimino)acenaphth-ene hexafluorophosphate: [Ir(4-MeO-5-Me-ppy)_2_(4-NMe_2_Ph-BI-AN)]^+^(PF_6_)^−^, **2:** Black crystals (0.073 g). **Yield:** 80%. Mp: 337–339 °C (turned into dark liquid). ^**1**^ **H NMR (500 MHz, CD**_**3**_ **CN) δ (ppm):** 8.39–8.34 (m, 2 H), 8.21 (d, *J* = 8.2 Hz, 2 H), 7.68 (d, *J* = 1.5 Hz, 2 H), 7.65 (s, 2 H), 7.57–7.53 (m, 2 H), 7.44 (d, *J* = 7.3 Hz, 2 H), 7.32 (d, *J* = 8.6 Hz, 2 H), 6.36 (dd, *J* = 8.6, 2.6 Hz, 10 H), 5.45 (s, 2 H), 3.50 (s, 6 H), 2.89 (s, 12 H), 2.28 (s, 6 H). ^**13**^ **C NMR (125 MHz, CDCl**_**3**_ **) δ (ppm):** 169.74, 165.16, 160.15, 150.75, 150.45, 148.60, 144.51, 143.45, 139.26, 136.83, 134.17, 132.07, 131.81, 130.68, 128.89, 127.70, 125.37, 123.54, 118.36, 117.32, 111.34, 107.02, 54.96, 40.51, 18.62. **HRMS (EI, 70 eV):** [M-PF_6_]^+^ **Calculated:** (C_54_H_50_IrN_6_O_2_) 1007.3623; **Found:** 1007.3646.

### Iridium (III) bis[3-methoxy-2-phenyl-5-methylpyridinato-N,C^2′^]-*N*,*N’*-bis(4-*N*,*N*-dimethyl-phenylimino)acenaphth-ene hexafluorophosphate: [Ir(3-MeO-5-Me-ppy)_2_(4-NMe_2_Ph-BIAN)]^+^(PF_6_)^−^, 3

Black crystals (0.116 g). **Yield:** 80%. Mp: 354 °C (turned into dark liquid). ^**1**^ **H NMR (500 MHz**, **CD**_**3**_ **CN) δ (ppm):** 8.41 (d, *J* = 1.7 Hz, 2 H), 8.21 (d, *J* = 8.2 Hz, 2 H), 7.78–7.67 (m, 5 H), 7.55 (t, *J* = 7.8 Hz, 2 H), 7.47 (d, *J* = 7.3 Hz, 2 H), 6.95 (d, *J* = 2.7 Hz, 2 H), 6.43–6.27 (m, 8 H), 5.90 (d, *J* = 8.3 Hz, 2 H), 5.45 (s, 1 H), 3.66 (s, 6 H), 2.89 (s, 12 H), 2.30 (s, 6 H). ^**13**^ **C NMR (125 MHz**, **CDCl**_**3**_ **) δ (ppm):** 169.79, 165.25, 156.29, 150.33, 149.15, 144.46, 143.53, 139.30, 138.46, 134.04, 133.52, 131.82, 131.73, 130.58, 128.87, 127.81, 123.46, 119.11, 116.78, 111.33, 109.21, 55.63, 40.47, 18.67. **HRMS (EI, 70 eV):** [M-PF_6_]^+^ **Calculated:** (C_54_H_50_IrN_6_O_2_) 1007.3623; **Found:** 1007.3625.

### Iridium (III) methyl-bis[3-methoxy-2-phenyl-5-methyl-pyridinato-N,C^2′^]-*N*,*N’*-bis(4-*N*,*N*-di-methylphenyl-imino)ace-naphthene-5-carboxylate hexafluorophosphate: [Ir(3-MeO-5-Me-ppy)_2_(4-NMe_2_Ph-BIAN-CO_2_Me)]^+^(PF_6_)^−^, 4

Black crystals (0.095 g). **Yield:** 73%. Mp: 353–355 °C (turned into dark liquid). ^**1**^ **H NMR (500 MHz, CD**_**3**_ **CN) δ (ppm):** 9.07 (dd, *J* = 8.7, 0.7 Hz, 1 H), 8.41 (dd, *J* = 1.9, 1.0 Hz, 2 H), 8.22 (d, *J* = 7.7 Hz, 1 H), 7.78–7.72 (m, 4 H), 7.67 (dd, *J* = 8.7, 7.4 Hz, 2 H), 7.63–7.57 (m, 2 H), 7.57–7.52 (m, 1 H), 6.98 (d, *J* = 2.6 Hz, 2 H), 6.52–6.05 (m, 8 H), 5.92 (dd, *J* = 9.8, 8.3 Hz, 2 H), 3.99 (s, 3 H), 3.69 (d, *J* = 2.4 Hz, 6 H), 2.93 (d, *J* = 5.7 Hz, 12 H), 2.33 (s, 6 H). ^**13**^ **C NMR (125 MHz, CDCl**_**3**_ **) δ (ppm):** 169.25, 168.03, 166.52, 165.14, 156.40, 150.70, 150.40, 149.10, 149.03, 144.37, 143.58, 143.51, 139.46, 139.40, 138.09, 133.94, 133.64, 132.93, 131.93, 131.75, 131.73, 130.43, 130.35, 130.11, 128.50, 128.35, 123.58, 121.78, 119.17, 116.85, 116.79, 111.28, 111.20, 109.19, 55.63, 52.84, 40.45, 40.42, 18.65. **HRMS (EI, 70 eV):** [M-PF_6_]^+^ **Calculated:** (C_56_H_52_IrN_6_O_4_) 1065.3678; **Found:** 1065.3668.

Synthesis of Ir(III) bis[3-methoxy-2-phenyl-5-methylpyridinato-N,C^2′^]-*N*,*N′*-bis(4-*N*,*N*-dimethylphenyl-imino)acenaphthene-5-carboxylate hexafluorophosphate, 5: Complex [Ir(3-MeO-5-Me-ppy)_2_(4-NMe_2_Ph-BIAN-CO_2_Me)]^+^ (PF_6_)^−^ (**4**) (0.050 g, 0.040 mmol, 1.0 equiv.) and NaOH (0.0050 g, 0.12 mmol, 2.7 equiv.) were dissolved with 6 mL H_2_O and 6 mL MeCN. The mixture was degassed three times, placed under N_2_, and heated to 85 °C for 22 h. The solution was cooled to RT and quenched with 1.2 mL of 0.10 N aqueous HCl. After careful removal of the solvent, the product was extracted with DCM (3 × 15 mL), evaporated to dryness, and the crude solid was re-dissolved in a minimum amount of MeOH. To this solution was slowly added a solution of NH_4_PF_6_ (10 mL, 6.13 mmol, 1 g/10 mL) under gentle stirring. Upon the first drop addition of aqueous NH_4_PF_6_ solution, the appearance of a greenish black precipitate was observed and continued until the addition was complete. Then, the greenish black solid suspension was conserved for 3 h at 0 °C and collected by a Buchner funnel filtration following washing with water and Et_2_O. The resulting residue was dried *in vacuo* to obtain a greenish dark solid powder. The crude product was recrystallised in dichloromethane/diisopropylether (50:50) by slow evaporation to obtain greenish black crystals. Greenish black crystals (0.047 g). **Yield:** 96%. Mp: 359–360 °C (turned into dark liquid). ^**1**^ **H NMR (400 MHz**, **CD**_**3**_ **CN) δ (ppm):** 9.12 (d, *J* = 8.6 Hz, 1 H), 8.42 (bs, 2 H), 8.25 (d, *J* = 7.7 Hz, 1 H), 7.76 (d, *J* = 2.1 Hz, 5 H), 7.65 (m, 2 H), 7.60–7.52 (m, 2 H), 6.98 (d, *J* = 2.6 Hz, 2 H), 6.47–6.24 (m, 8 H), 5.97–5.88 (m, 2 H), 3.69 (d, *J* = 1.7 Hz, 6 H), 2.94 (s, 12 H), 2.33 (s, 6 H). ^**13**^ **C NMR (125 MHz**, **CD**_**3**_ **CN) δ (ppm):** 170.44, 169.38, 166.52, 164.41, 156.27, 150.49, 150.29, 144.72, 144.10, 144.06, 139.16, 137.92, 137.88, 134.23, 134.19, 132.74, 132.58, 130.10, 130.02, 129.41, 128.99, 128.09, 127.04, 123.21, 121.59, 118.90, 118.89, 115.86, 115.83, 111.13, 111.11, 109.99, 108.97, 54.99, 39.61, 39.58, 17.33, 17.31. **HR-MS (EI**, **70 eV): [M-PF**_**6**_ **]** ^**+**^ **Calculated:** (C_55_H_50_IrN_6_O_4_) 1051.3521; **Found:** 1051.3519.

### Data availability

Supplementary Information available: General procedures, experimental details, photophysical and electrochemical characterisation protocols, ^1^H and ^13^C NMR spectra, and computational details. The data supporting this study are available at: 10.17630/c28fddad-6877-4c31-80fa-f88600b735ae.

## Electronic supplementary material

Supplementary Information 
