# Supplementary material for: Bay-Region Functionalisation of Ar-BIAN Ligands and Their Use Within Highly Absorptive Cationic Iridium(III) Dyes
Source: Sci Rep. 2017 Nov 14;7:15520. doi: 10.1038/s41598-017-14996-4 (PMC5686200; doi:10.1038/s41598-017-14996-4)
Supplement: Supplementary file 1 — Supplementary Information [file 41598_2017_14996_MOESM1_ESM.pdf]

Bay-Region Functionalisation of Ar-BIAN Ligands and Their Use Within Highly Absorptive Cationic Iridium(III) Dyes

Kamrul Hasan,[a,b] Jingyi Wang,[c] Amlan K. Pal,[d] Claus Hierlinger,[d,e] Véronique Guerchais,[e] Han Sen Soo,[c,f]* Felipe García,[c]* and Eli Zysman-Colman[a,d]*

[a] Département de Chimie, Université de Sherbrooke, 2500 Boul. de l'Université, Sherbrooke, QC, Canada, J1K 2R1;

[b] Department of Chemistry, College of Sciences, University of Sharjah, Sharjah, P. O. Box 27272, UAE;

[c] Division of Chemistry and Biological Chemistry, School of Physical and Mathematical Sciences, Nanyang Technological University, 21 Nanyang Link 637371, Singapore;

[d] Organic Semiconductor Centre, EaStCHEM School of Chemistry, University of St Andrews, St Andrews, Fife, KY16 9ST, UK, Fax: +44-1334 463808; Tel: +44-1334 463826

[e] Institut des Sciences Chimiques de Rennes, UMR 6226 CNRS-Université de Rennes 1, Campus de Beaulieu, 35042 Rennes Cedex, France. E-mail: veronique.guerchais@univ-rennes1.fr.

[f] Solar Fuels Laboratory, Nanyang Technological University, 50 Nanyang Avenue, Singapore 639798;

SUPPLEMENTARY INFORMATION

Table of Contents:

	Pages
General procedures	S2
Synthesis of compounds **6, 10** and **11**	S2-S3
Synthesis of (C^N) ligands	S3-S4
Photophysical and electrochemical characterisation protocols	S4-S5
Steady-state photoluminescence measurements	S5
Fluorescence quantum yield measurements	S5
Transient absorption and emission spectroscopic measurements	S6
^{1}H and ^{13}C NMR spectra	S7-S24
Computational section	S25-S43
Transient absorption, emission, and steady-state photoluminescence spectra	S44-S46
References	S47-S48

General Procedures

Commercial chemicals were used as supplied. All reactions were performed using standard Schlenk techniques under inert (N_2) atmosphere with freshly distilled anhydrous solvents obtained from a Pure Solv™ solvent purification system from Innovative Technologies, except where specifically mentioned. The iridium dimer $[(ppy)_2Ir\text{-}(\mu\text{-}Cl)]_2$[1] and *p*-$NMe_2$Ph-BIAN[2] were prepared following the previously published procedures. Flash column chromatography was performed using silica gel (Silia-P from Silicycle, 60 Å, 40-63 μm). Analytical thin layer chromatography (TLC) was performed on silica plates with aluminum backings (250 μm with indicator F-254). Compounds were visualised under UV light. The ^{1}H and ^{13}C NMR spectra were recorded on a Bruker Avance spectrometer at 500 MHz for complexes **2-4**, 400 MHz for complex **5** and 125 MHz for all the complexes, respectively. The following abbreviations have been used for multiplicity assignments: "s" for singlet, "d" for doublet, "t" for triplet, "m" for multiplet, and "br" for broad. Deuterated chloroform ($CDCl_3$), deuterated acetonitrile (CD_3CN) were used as the solvents for the NMR spectroscopic measurements. Melting points (Mp's) were recorded using open-ended capillaries on a Meltemp melting point apparatus and were uncorrected. Each of the high-resolution mass spectrum (HRMS) was recorded on a quadrupole time-of-flight (ESI-Q-TOF) model Maxis from Bruker in positive electrospray ionisation mode, using sodium formate solution as a calibrant.

Synthesis of compounds 6, 10 and 11

5-bromo-1,2-dihydroacenaphthene, 6: To a 0 °C solution of acenaphthene (5.20 g, 33.7 mmol, 1.0 equiv.) in 50 mL acetonitrile (MeCN) was added dropwise, a solution of *N*-bromosuccinimide (NBS) (6.00 g, 33.7 mmol, 1.0 equiv.). The solution was allowed to warm to room temperature (RT) and stirred for 19 h. The reaction was quenched with 100 mL of H_2O, hexanes were added, and the layers separated. The aqueous phase was extracted 3 x 100 mL hexanes. The organic phase was dried over $MgSO_4$, filtered under vacuum, and concentrated under reduced pressure to obtain the desired product. The crude product (R_f of 0.3, DCM on silica) was purified by column chromatography with silica gel using DCM/hexanes (1:1). A yellow solid (7.2 g) was collected. Yield: 91%. Mp 52 °C. ^{1}H NMR (400 MHz, $CDCl_3$) δ (ppm): 7.77 (d, J = 8.37 Hz, 1 H), 7.66 (d, J = 7.34 Hz, 1 H), 7.55 (t, J = 8.34 Hz, 1 H), 7.33 (d, J = 6.85 Hz, 1 H),

7.14 (dt, J = 1.35, 7.35 Hz, 1 H), 3.42 (m, 2 H), 3.34 (m, 2 H). ^{13}C NMR (100 MHz, $CDCl_3$) δ (ppm): 147.2, 146.5, 140.5, 131.1, 129.3, 122.0, 120.4, 120.3, 117.0, 30.9, 30.2. HRMS (EI, 70 eV): $[M]^+$Calculated: ($C_{12}H_9Br$) 231.9888; Found: 231.9892. This compound has been previously reported where characterisation was limited to a melting point (Mp: 96-98 °C).[3] However, the melting point for commercially available 5-bromo-1,2-dihydroacenaphthene is 54-56 °C, which is in line with our experimental results 52 °C.

4-*N,N*-dimethylaminonitrobenzene, 10: This compound was prepared in accordance to a previously published procedure.[4] Lemon yellow needle like crystals (2.57 g) were collected. Yield: 55%. Mp 165 °C. ^{1}H NMR (400 MHz, $CDCl_3$) δ (ppm): 8.09 (m, 2 H), 6.58 (m, 2 H), 3.10 (s, 6 H). The characterisation matches that reported.[5]

***N,N*-dimethylaminoaniline, 11:** This compound was prepared by adapting a previously published protocol[6] to yield black solid (0.802 g). Yield: 85%. ^{1}H NMR (400 MHz, $CDCl_3$) δ (ppm):6.69 (d, J = 9 Hz), 6.67 (d, J = 9 Hz), 3.36 (s, 2 H), 2.82 (s, 6 H). The characterisation matches that reported.[7]

Synthesis for the modification of cyclometallating (C^N) ligands:

2-(4'-methoxyphenyl)-5-methylpyridine:[8] The precursor *p*-methoxybenzeneboronic acid was prepared from 4-bromoanisole following a previously reported procedure.[9] Compound 2-bromo-5-methylpyridine (0.595 g, 3.85 mmol, 1.0 equiv.), K_2CO_3 (1.43 g, 10.3 mmol, 3.0 equiv.), and *p*-methoxybenzeneboronic acid (0.657 g, 4.48 mmol, 1.25 equiv.) were dissolved in degassed dioxane/H_2O (40 mL/10 mL). Subsequently, $Pd(PPh_3)_4$ (0.200 g, 0.18 mmol, 5 mol%) was added and the solution was degassed three times. The reaction mixture was then heated at reflux for 19 h. Upon cooling, H_2O (50 mL) was added and the crude product was extracted with DCM (50 mL). The organic layer was separated and dried over anhydrous $MgSO_4$. Removal of the solvent under reduced pressure gave a dark slurry (product R_f of 0.25, 10% EtOAc/hexanes on silica), which was purified by column chromatography on silica gel (EtOAc/hexane, 1:9) to afford an off-white solid (0.675 g). Yield: 95%. ^{1}H NMR (400 MHz, $CDCl_3$) δ (ppm): 8.48 (s, 1 H), 7.92 (m, 2 H), 7.54 (dt, J = 4.95, 8.17 Hz, 2 H), 6.99 (m, 2 H), 3.86 (s, 3 H), 2.35 (s, 3 H). The characterisation matches that previously reported.[10,11]

2-(3'-methoxyphenyl)-5-methylpyridine:[12] The precursor *m*-methoxybenzeneboronic acid was prepared from 3-bromoanisole following a previously reported procedure.[13] 2-Bromo-5-methylpyridine (1.84 g, 10.7 mmol, 1.0 equiv.), K_2CO_3 (4.43 g, 32.1 mmol, 3.0 equiv.) and *m*-methoxybenzeneboronic acid (1.96 g, 13.4 mmol, 1.25 equiv.) were dissolved in degassed dioxane/H_2O (5:1 v/v) in order to obtain a 0.05 M concentration in 2-bromo-5-methylpyridine. $Pd(PPh_3)_4$ (0.60 g, 0.54 mmol, 5 mol%) was then added and the solution was degassed three times. The reaction mixture was heated at reflux for 19 h. Upon cooling, the solution was quenched with equal volume of H_2O and diluted with equal volume of DCM. The organic layer was separated and dried over anhydrous $MgSO_4$, filtered under vacuum, and concentrated under reduced pressure to obtained desired product (R_f of 0.20, 10% EtOAc/hexanes on silica). A colorless, oily liquid (0.996 g) was collected. Yield: 47%. ^{1}H NMR (400 MHz, $CDCl_3$) δ (ppm): 8.51 (dd, J = 0.70, 1.85 Hz, 1 H), 7.61 (d, J = 8.06 Hz, 1 H), 7.54 (m, 2 H), 7.51 (ddd, J = 0.87, 1.46, 7.68, 1 H), 7.36 (m, 1 H), 6.94 (t, J = 8.17 Hz, 1 H), 3.88 (s, 3 H), 2.36 (s, 3 H). The characterisation matches that previously reported.[14]

Photophysical and Electrochemical Characterisation protocols: All samples were prepared in HPLC grade MeCN with varying concentrations on the order of μM. Absorption spectra were recorded at RT using a Shimadzu UV-1800 double beam spectrophotometer. Molar extinction coefficients were determined by a linear least-squares fit of values obtained from the absorbances measured from at least three independent solutions at varying concentrations ranging from 6.81×10^{-1} to 2.39×10^{2} μM.

Cyclic voltammetry measurements were performed on an Electrochemical Analyzer potentiostat model 600D from CH Instruments. Solutions for cyclic voltammetry were prepared in MeCN and by nitrogen bubbling for about 10 min prior to the measurements. Tetra(*n*-butyl)ammoniumhexafluorophosphate ($TBAPF_6$; ca. 0.10 M in MeCN) was used as the support-

ing electrolyte. A non-aqueous Ag/Ag^+ electrode (silver wire in a solution of 0.10 M $AgNO_3$ in MeCN) was used as the pseudoreference electrode; a glassy-carbon electrode was used for the working electrode, and a Pt electrode was used as the counter electrode. The redox potentials are reported relative to a normal hydrogen electrode (NHE) electrode with a ferrocenium/ferrocene (Fc^+/Fc) redox couple as an internal reference (0.63 V vs NHE).[15]

Steady-state photoluminescence measurements

The steady-state photoluminescence (PL) measurements for **4** (in DCM and MeCN) and **5** (in DCM) were conducted using a Cary Eclipse Fluorescence Spectrophotometer from Varian Technologies. The samples were prepared, each with a concentration of 0.050 mM, in a glovebox under a N_2 atmosphere. The excitation and emission slit widths were opened to the maximum of 10 nm for all PL measurements of **4** and **5** due to their weak signals.

Fluorescence quantum yield measurements

The fluorescence quantum yield Φ for **4** was obtained by comparing its PL intensity with that from $[Ru(bpy)_3](PF_6)_2$ in a 0.050 mM DCM solution. The calculation was conducted using the following expression:

$$\Phi = \Phi_{\mathrm{ref}} \frac{\mathrm{I}}{\mathrm{A}} \frac{\mathrm{A}ref}{\mathrm{I}ref}$$

where Φ_{ref} is the quantum yield of the reference compound (Φ_{ref} = 0.029 for $[Ru(bpy)_3](PF_6)_2$ in DCM);[19] I and I_{ref} refer to the integrated fluorescence intensities of the sample and the reference compound respectively; A and A_{ref} are the absorbances at the wavelength of excitation for the sample and for the reference compound respectively.

Transient absorption and emission spectroscopic measurements

The transient absorption and emission spectra were obtained using a LP920 transient absorption spectrometer from Edinburgh instrument, with a power of 1.39 kV and a Q-switch delay of 106 μs. Each sample was prepared with a concentration of 0.050 mM in a glovebox under a N_2 atmosphere. UV-vis spectra were collected before and after the transient absorption/emission spectroscopic measurements to ensure that minimal compound decomposition occurred during these studies.

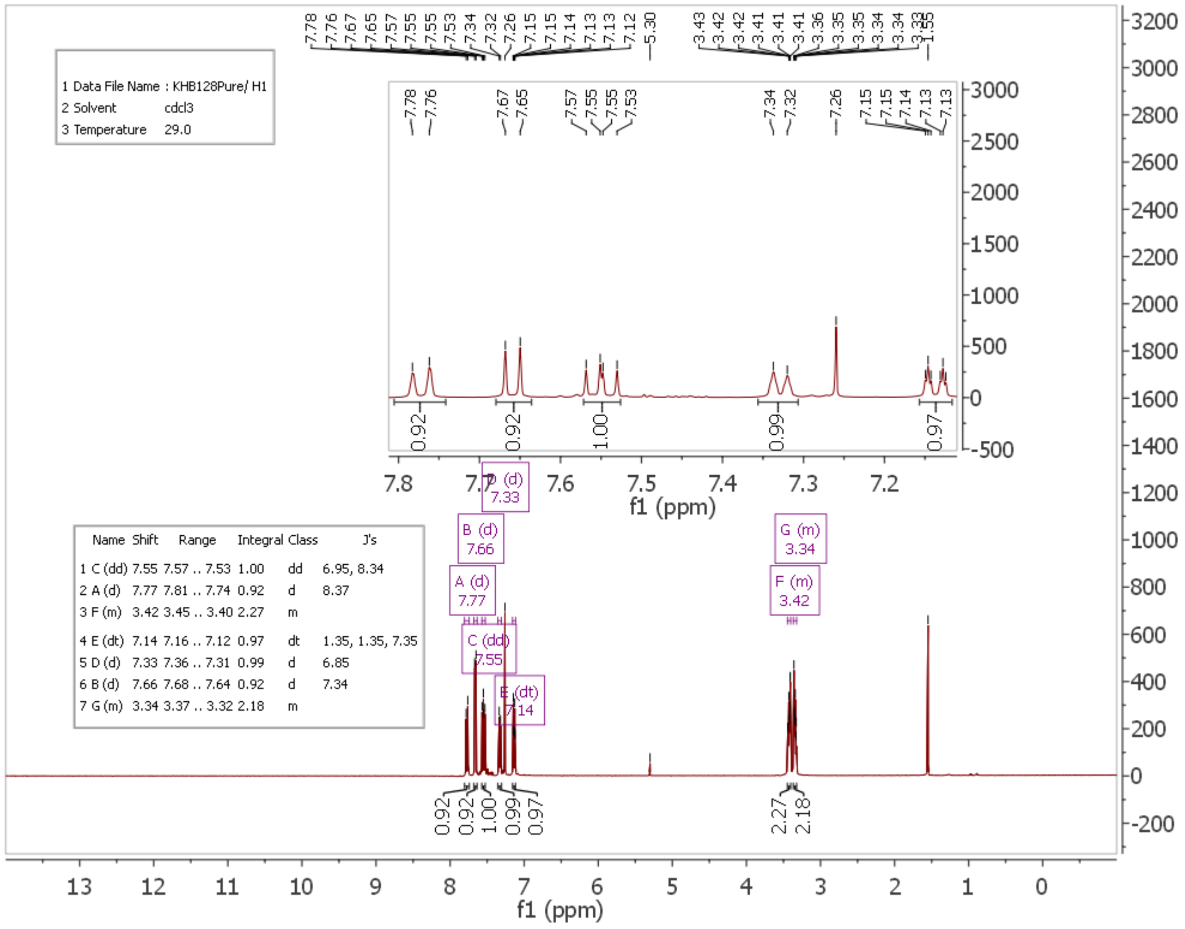

Supplementary Figure S1. ^{1}H NMR spectrum of 5-bromo-1,2-dihydroacenaphthylene (**6**) in $CDCl_3$

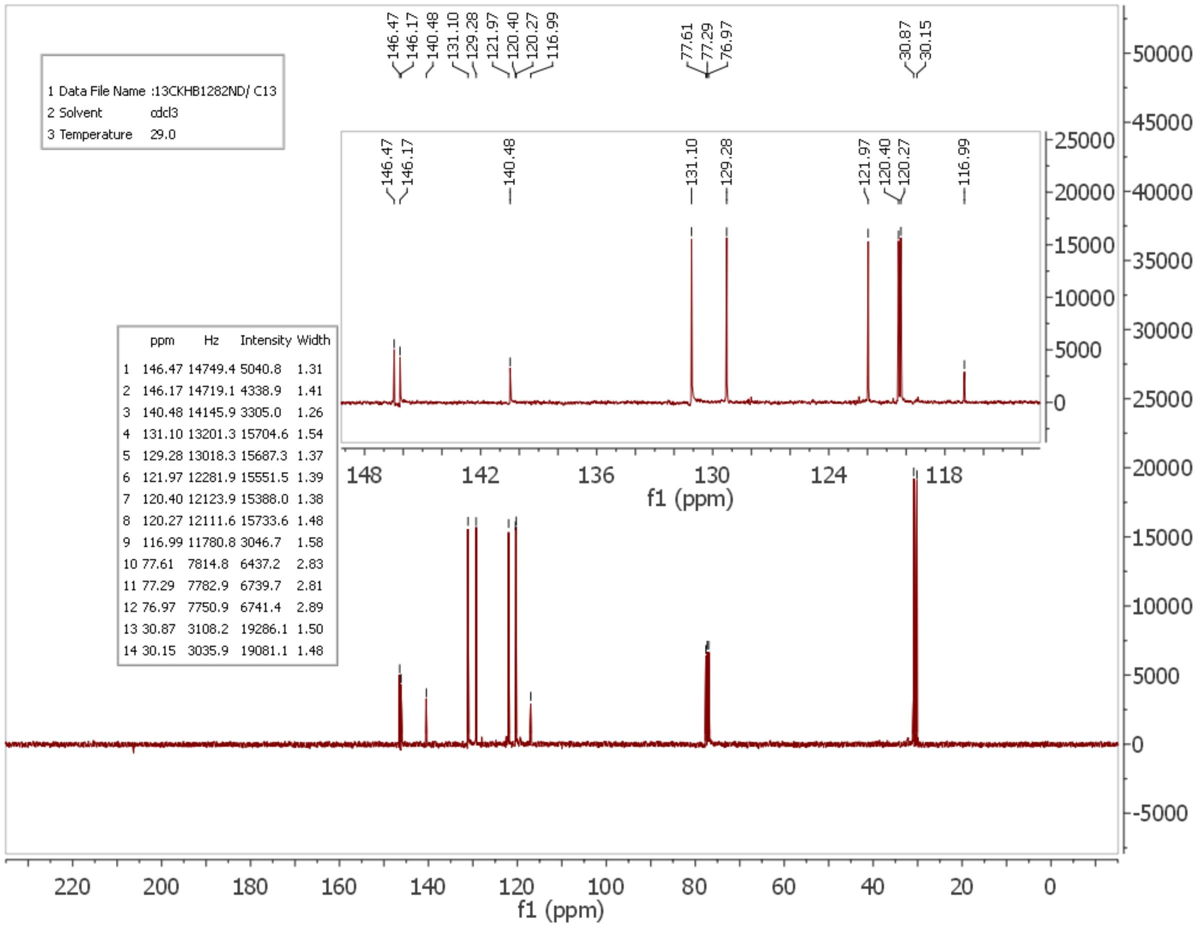

	ppm	Hz	Intensity	Width
1	146.47	14749.4	5040.8	1.31
2	146.17	14719.1	4338.9	1.41
3	140.48	14145.9	3305.0	1.26
4	131.10	13201.3	15704.6	1.54
5	129.28	13018.3	15687.3	1.37
6	121.97	12281.9	15551.5	1.39
7	120.40	12123.9	15388.0	1.38
8	120.27	12111.6	15733.6	1.48
9	116.99	11780.8	3046.7	1.58
10	77.61	7814.8	6437.2	2.83
11	77.29	7782.9	6739.7	2.81
12	76.97	7750.9	6741.4	2.89
13	30.87	3108.2	19286.1	1.50
14	30.15	3035.9	19081.1	1.48

Supplementary Figure S2. ^{13}C NMR spectrum of 5-bromo-1,2-dihydroacenaphthylene (**6**) in $CDCl_3$

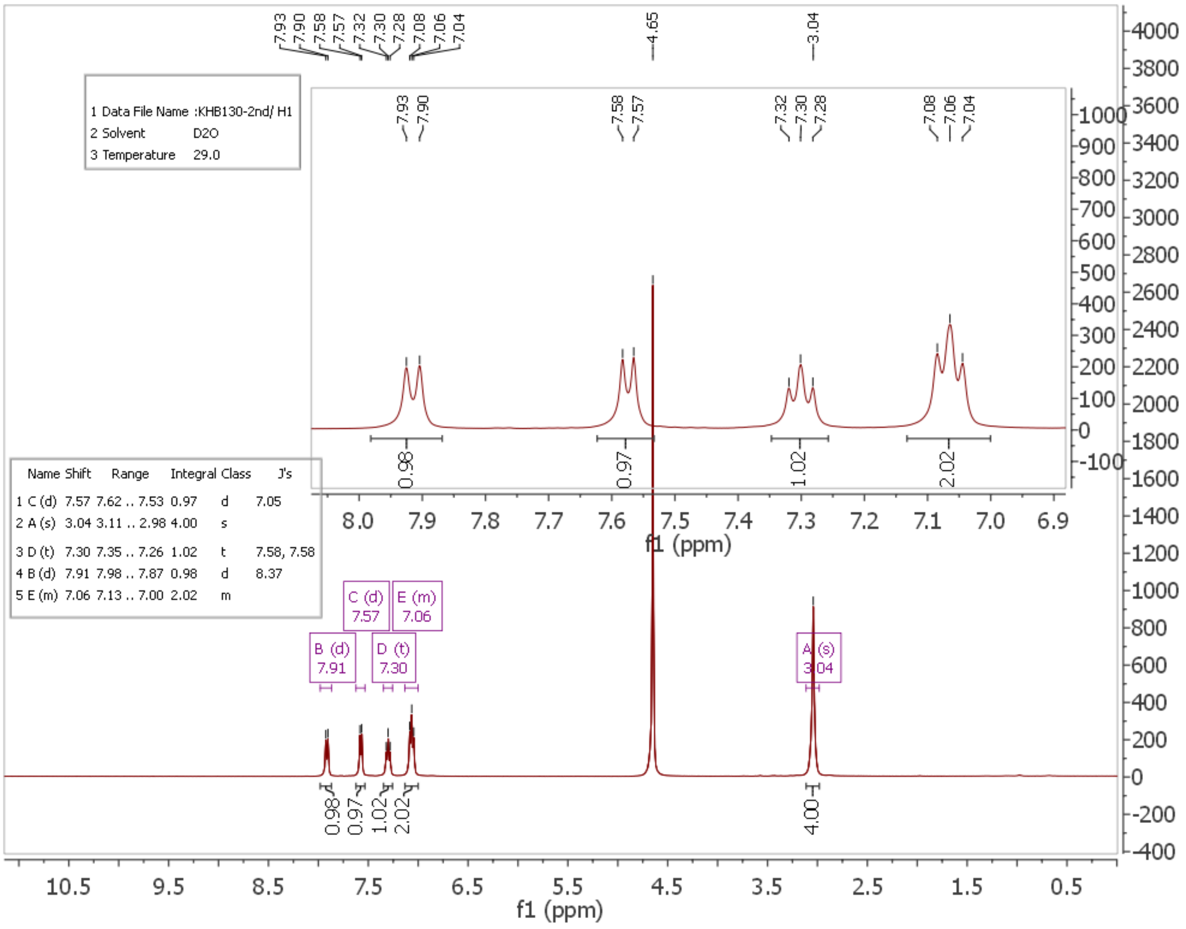

Supplementary Figure S3. ^{1}H NMR spectrum of 5-carboxy-1,2-dihydroacenaphthylene (**7**) in D_2O

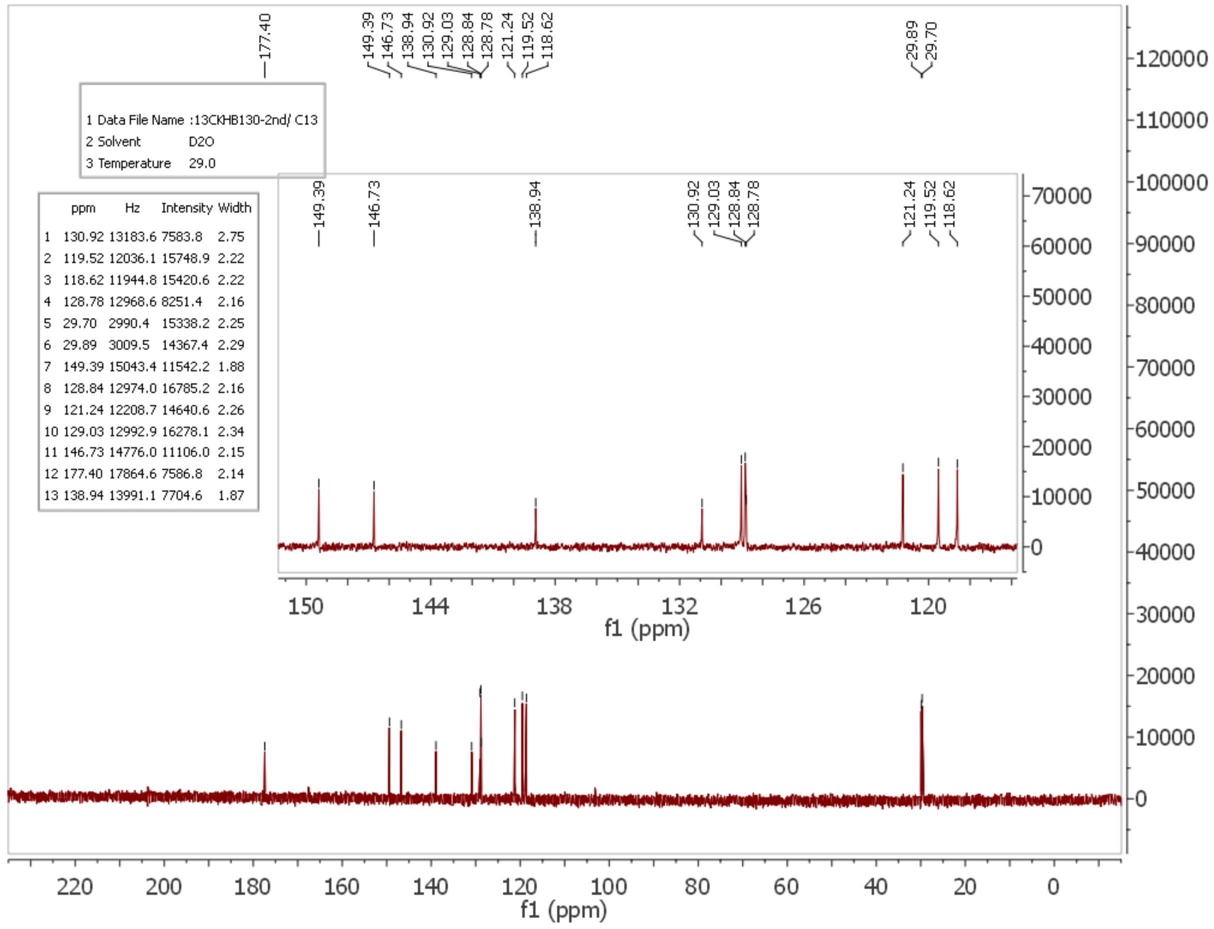

Supplementary Figure S4. ^{13}C NMR spectrum of 5-carboxy-1,2-dihydroacenaphthylene (**7**) in D_2O

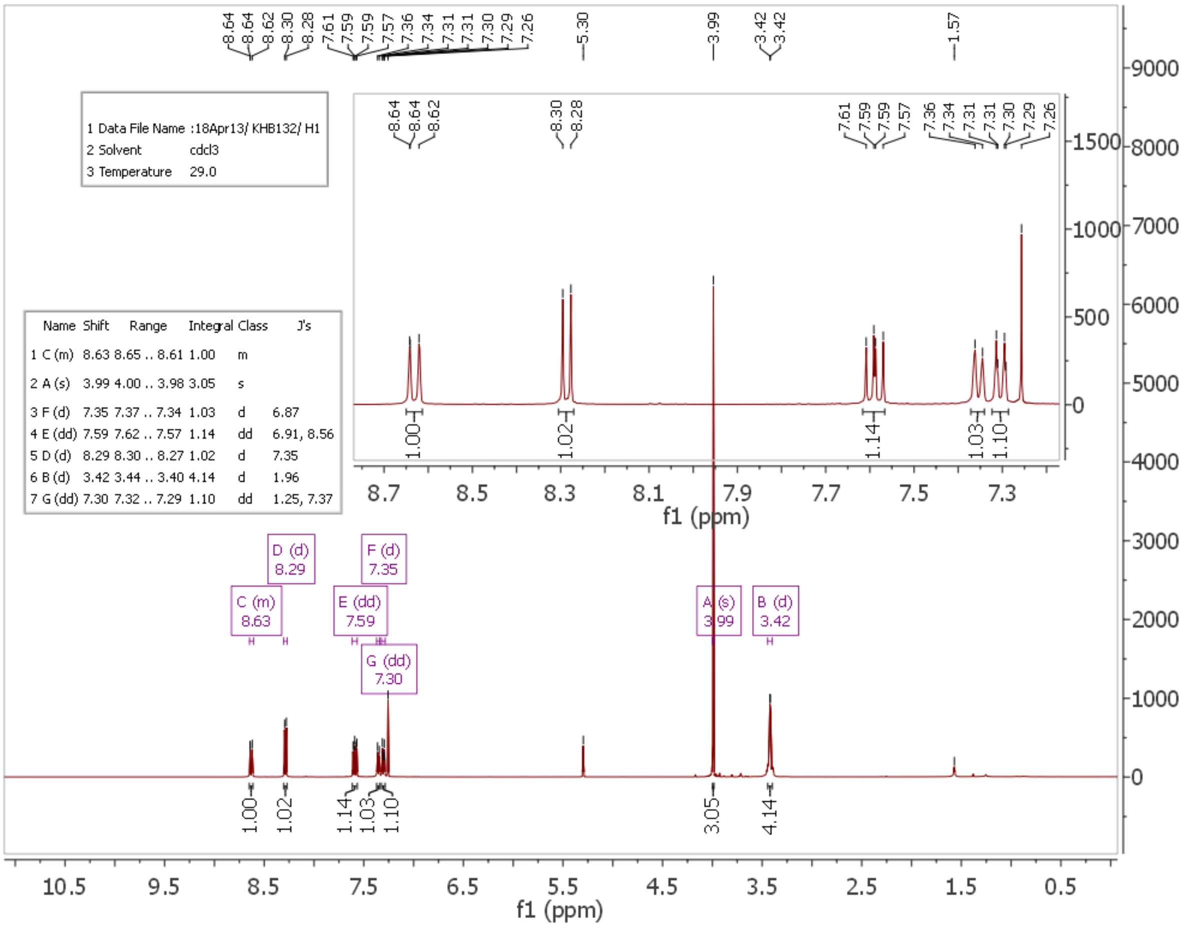

Supplementary Figure S5. ^{1}H NMR spectrum of 5-methylcarboxylate-1,2-dihydroacenaphthylene (**8**) in $CDCl_3$

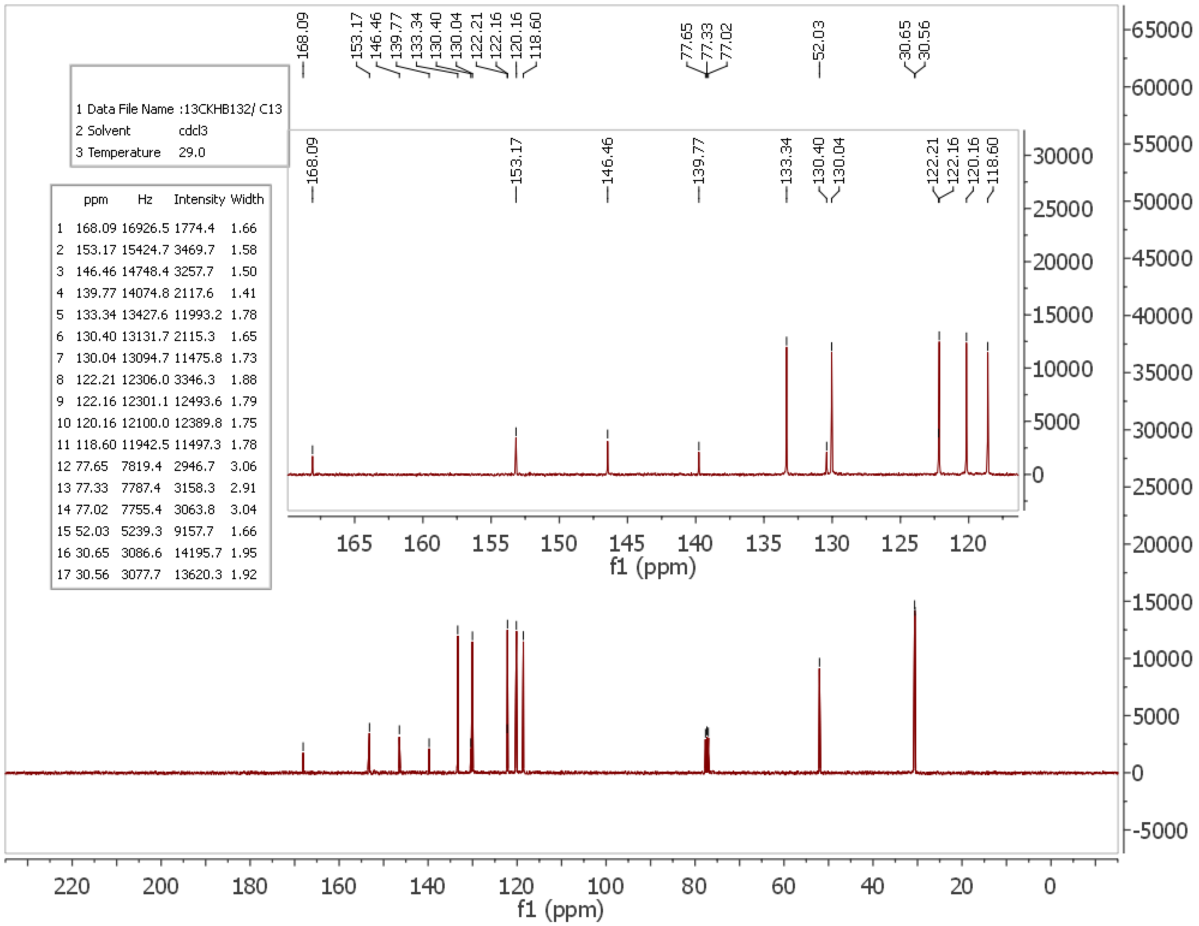

Supplementary Figure S6. ^{13}C NMR spectrum of 5-methylcarboxylate-1,2-dihydroacenaphthylene (**8**) in $CDCl_3$

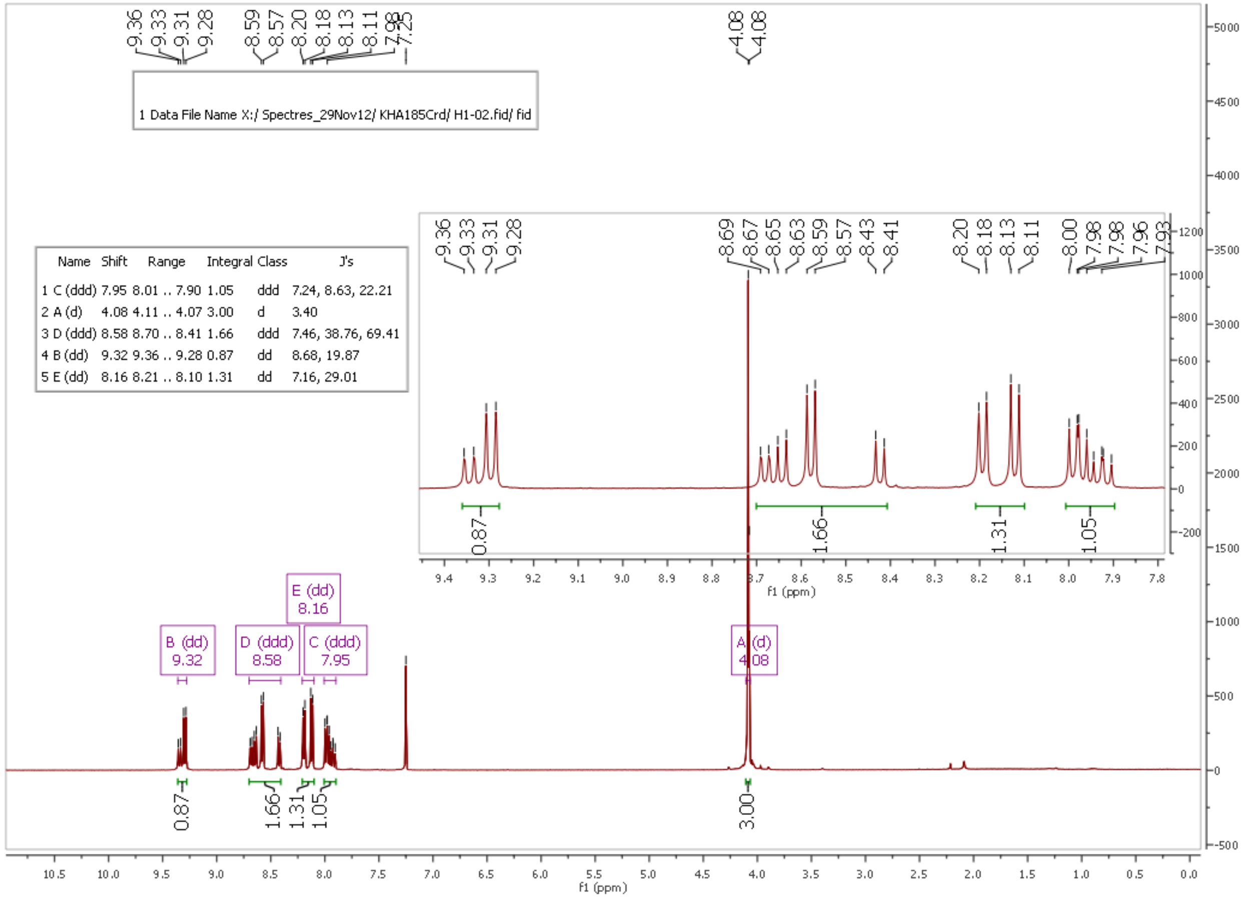

Supplementary Figure S7. ^{1}H NMR spectrum of 5-methylcarboxylate-1,2-dioxo-1,2-dihydroacenaphthylene (**9**) in $CDCl_3$

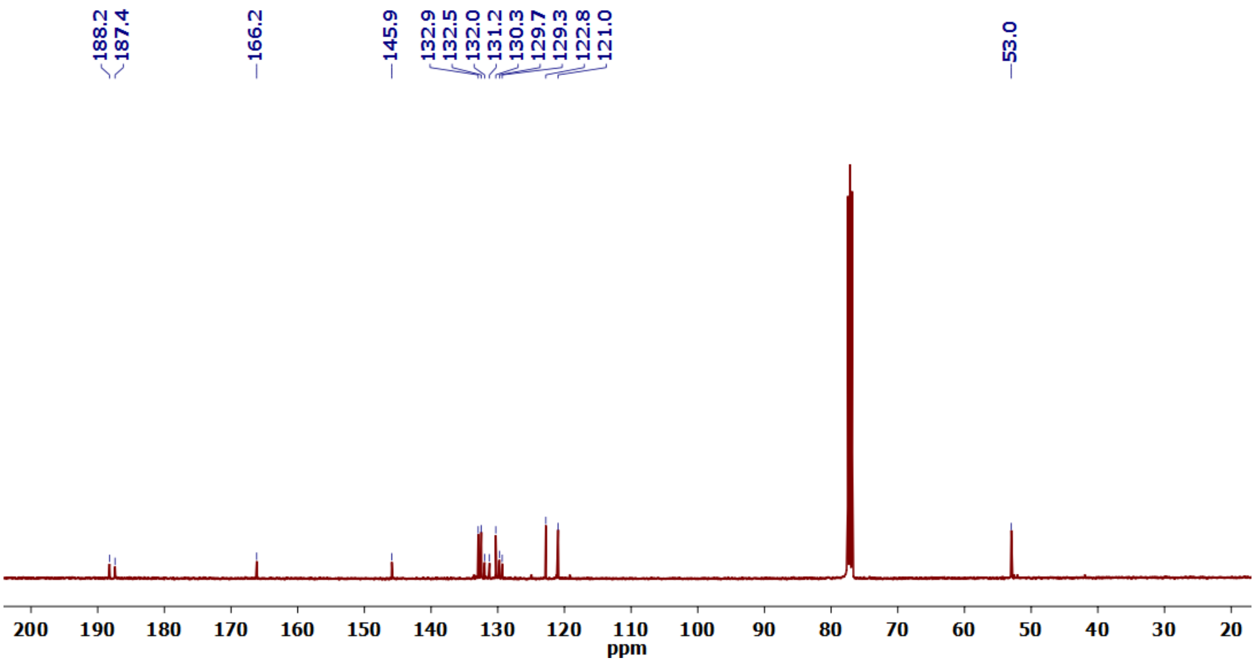

Supplementary Figure S8. ^{13}C NMR spectrum of 5-methylcarboxylate-1,2-dioxo-1,2-dihydroacenaphthylene (**9**) in $CDCl_3$

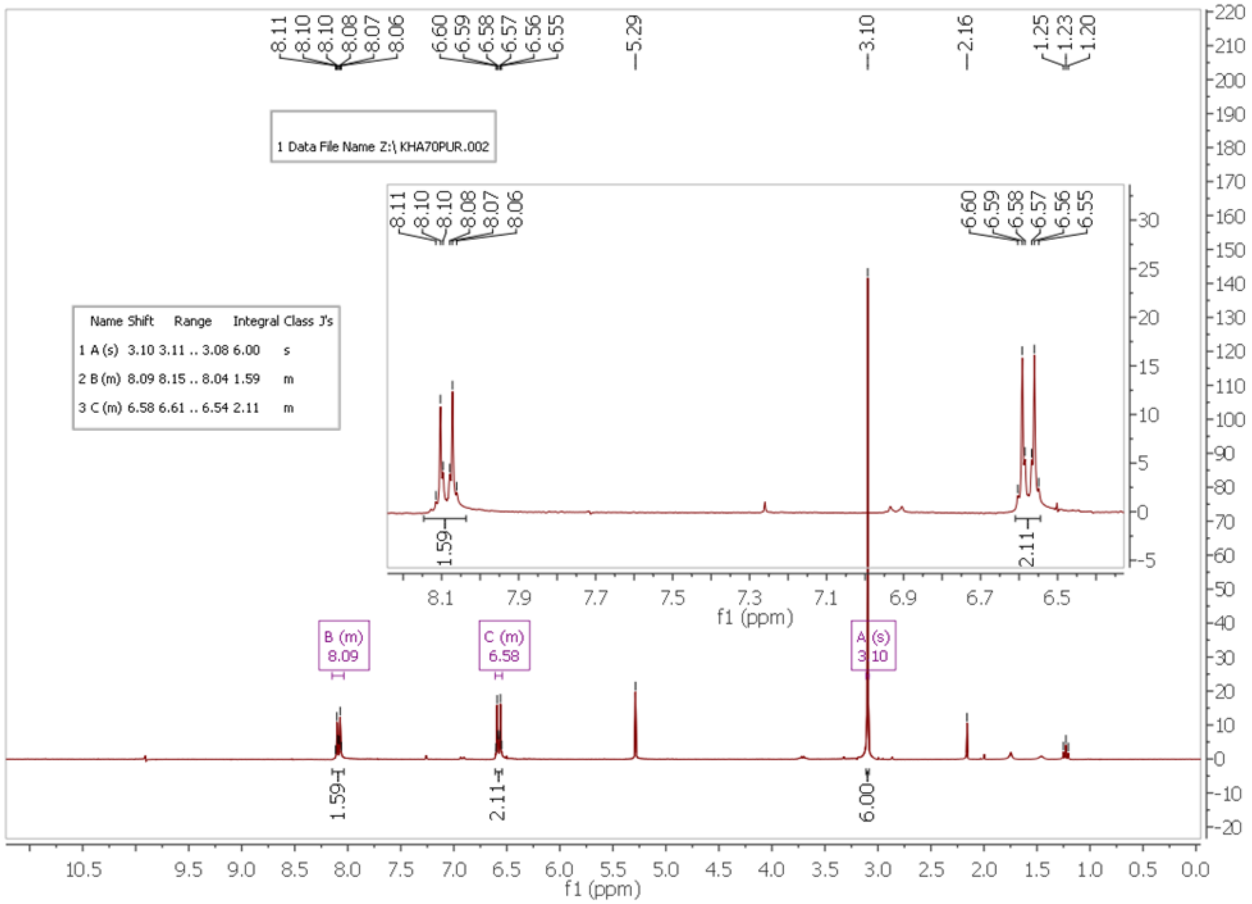

Supplementary Figure S9. ^{1}H NMR spectrum of *N,N*-dimethylamino-*p*-nitrobenzene (**10**) in $CDCl_3$

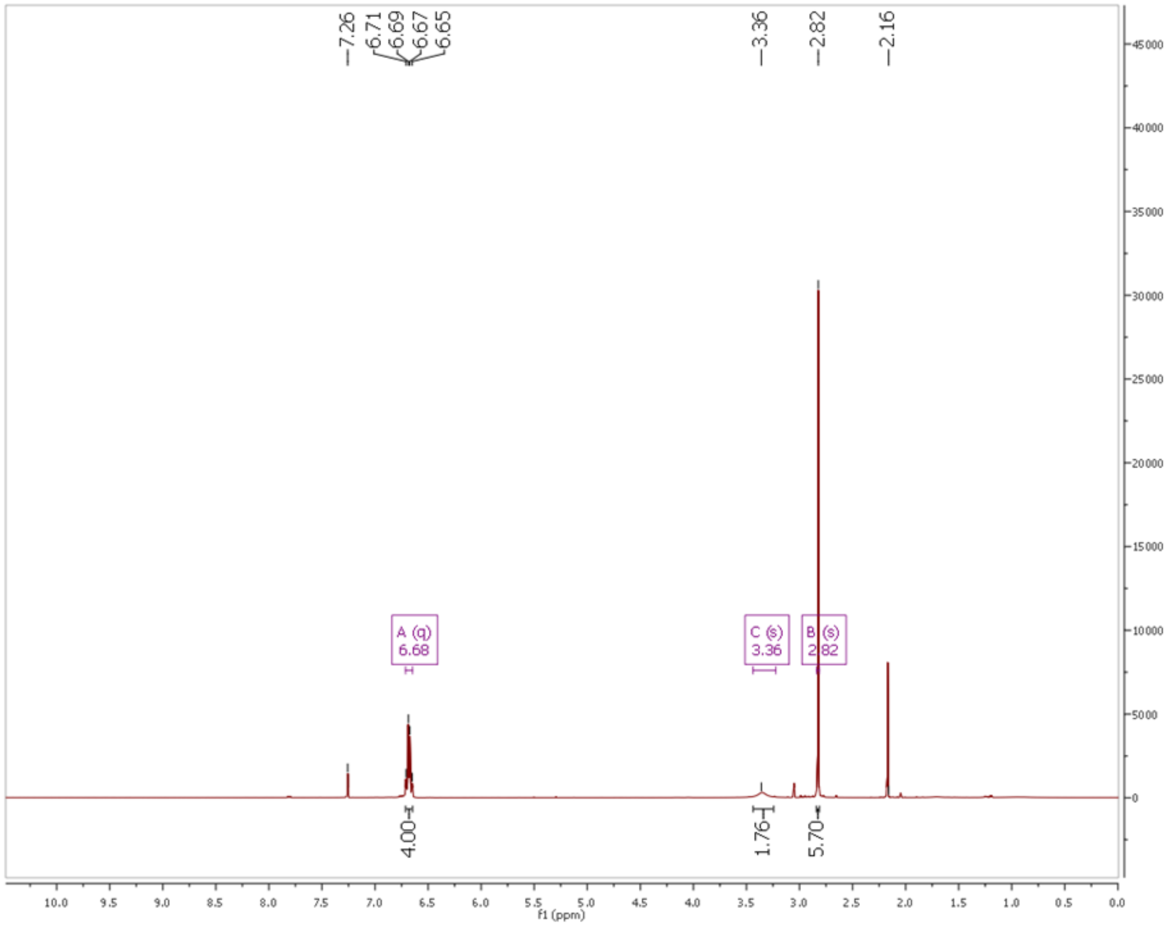

Supplementary Figure S10. ^{1}H NMR spectrum of *N,N*-dimethylamino-*p*-aniline (**11**) in $CDCl_3$

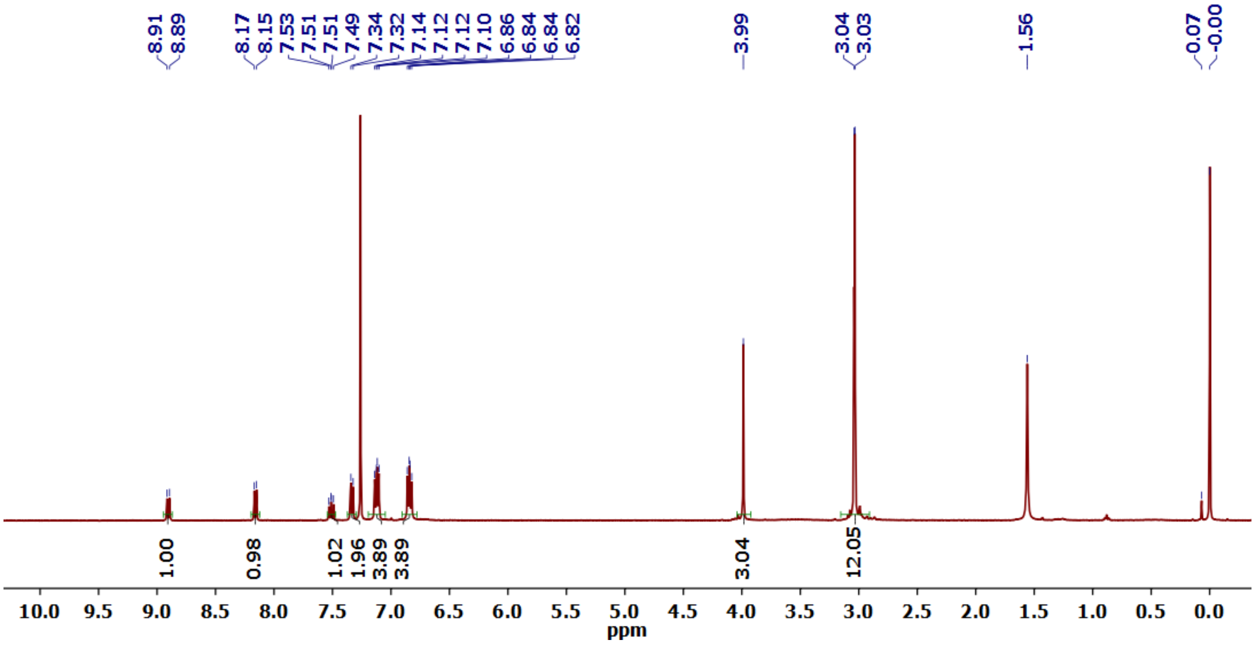

Supplementary Figure S11. ^{1}H NMR spectrum of (1*E*,2*E*)-methyl 1,2-bis((4-(dimethylamino)phenyl)imino)-1,2-dihydroacenaphthylene-5-carboxylate (4-Me2NPh-CO2MeBIAN) (**12**) in $CDCl_3$.

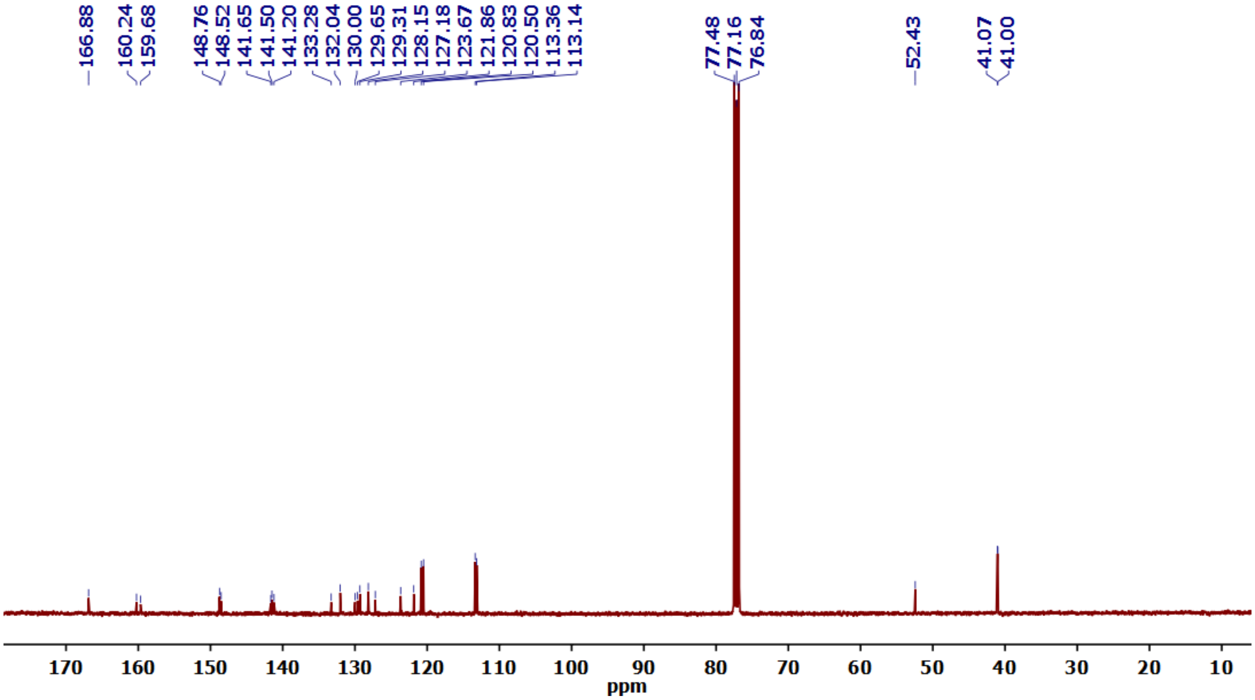

Supplementary Figure S12. ^{13}C NMR spectrum of (1*E*,2*E*)-methyl 1,2-bis((4-(dimethylamino)phenyl)imino)-1,2-dihydroacenaphthylene-5-carboxylate (4-Me2NPh-CO2MeBIAN) (**12**) in $CDCl_3$.

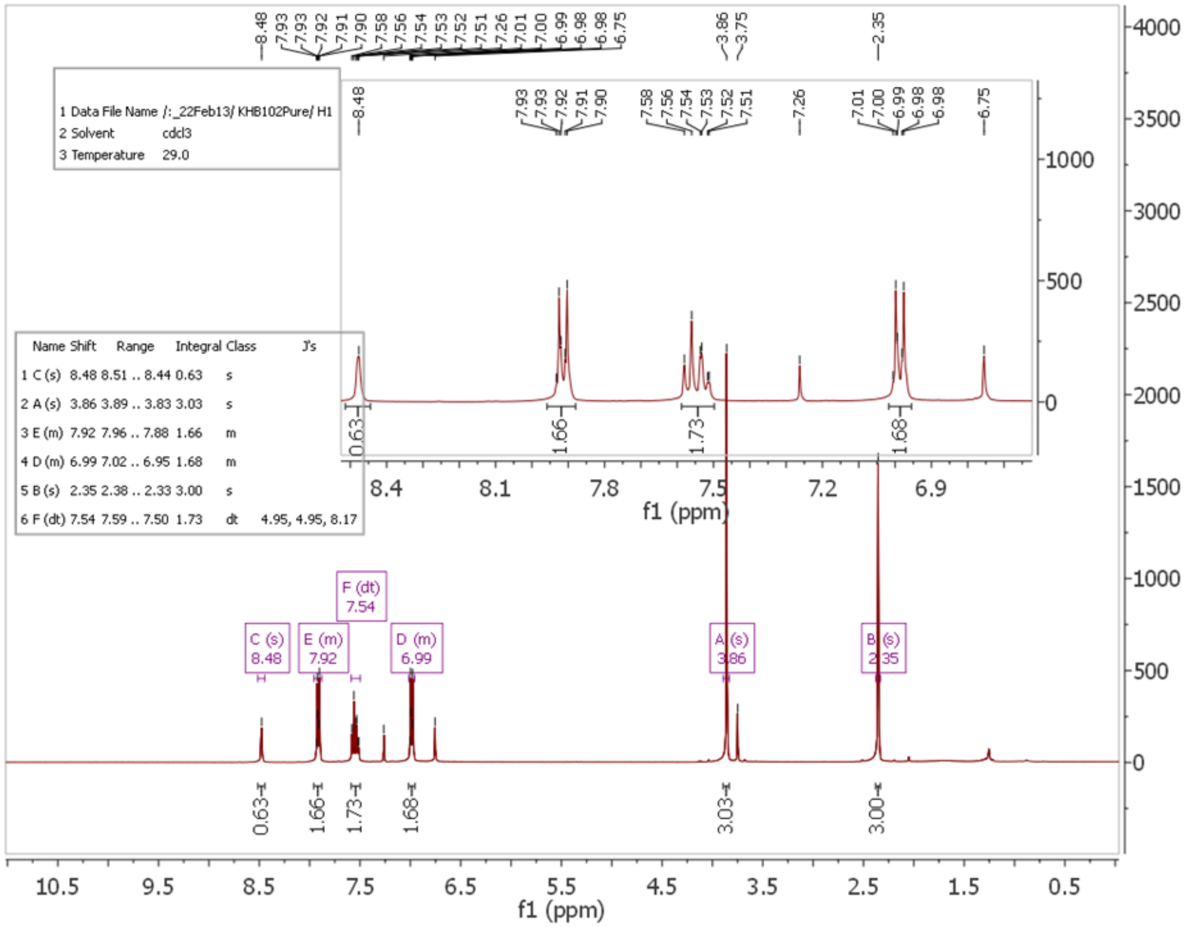

Supplementary Figure S13. ^{1}H NMR spectrum of 2-(4'-methoxyphenyl)-5-methylpyridine in $CDCl_3$

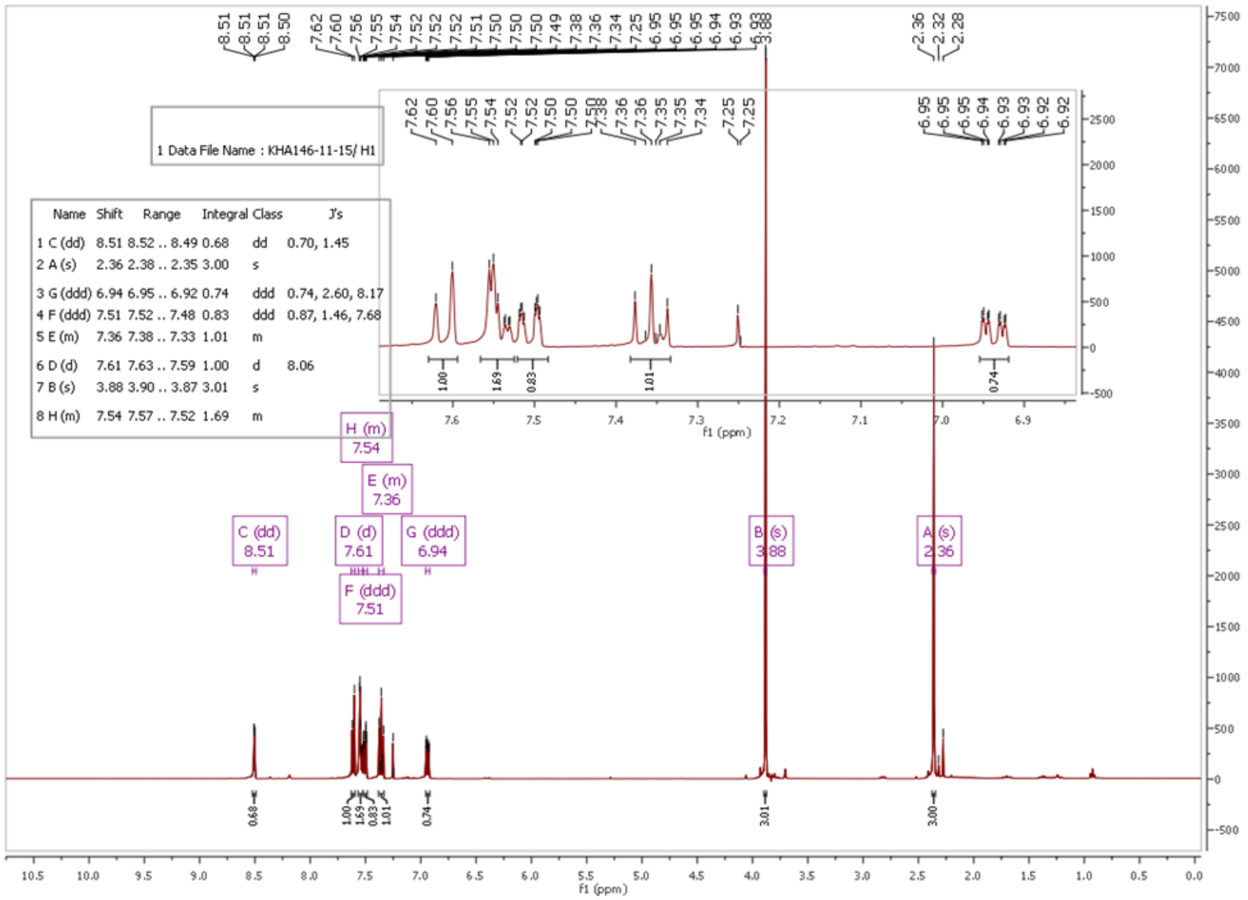

Supplementary Figure S14. ^{1}H NMR spectrum of 2-(3'-methoxyphenyl)-5-methylpyridine in $CDCl_3$

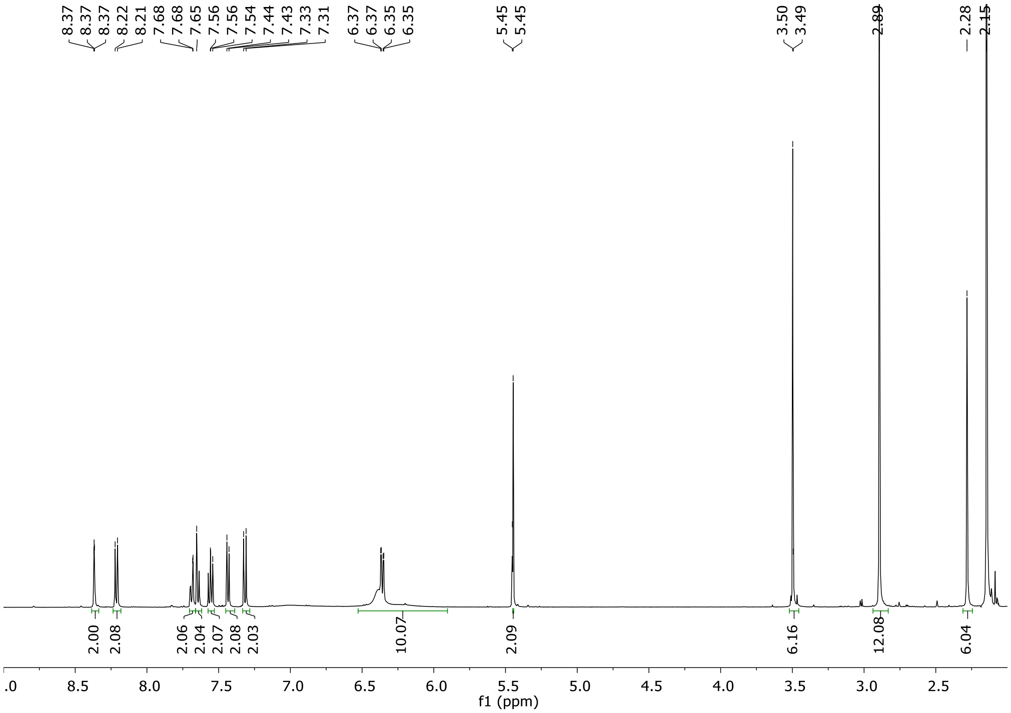

Supplementary Figure S15. ^{1}H NMR spectrum of [Ir(4-MeO-5-Me-ppy)$_2$(4-NMe$_2$Ph-BIAN)](PF$_6$) (**2**) in CD_3CN.

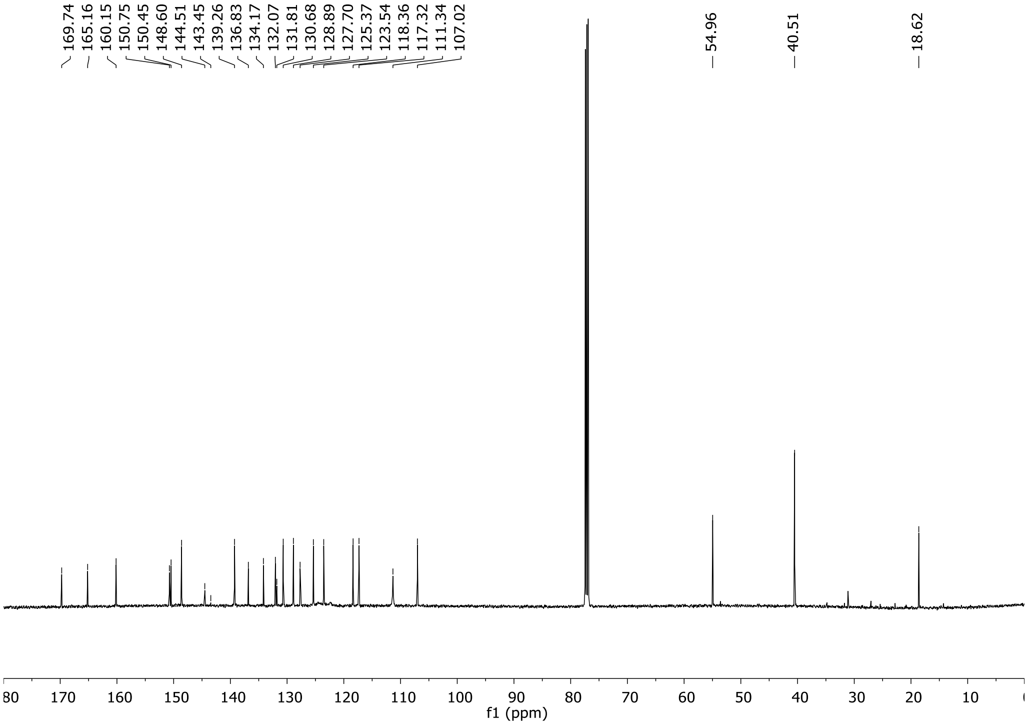

Supplementary Figure S16. ^{13}C NMR spectrum of [Ir(4-MeO-5-Me-ppy)$_2$(4-NMe$_2$Ph-BIAN)](PF$_6$) (**2**) in $CDCl_3$

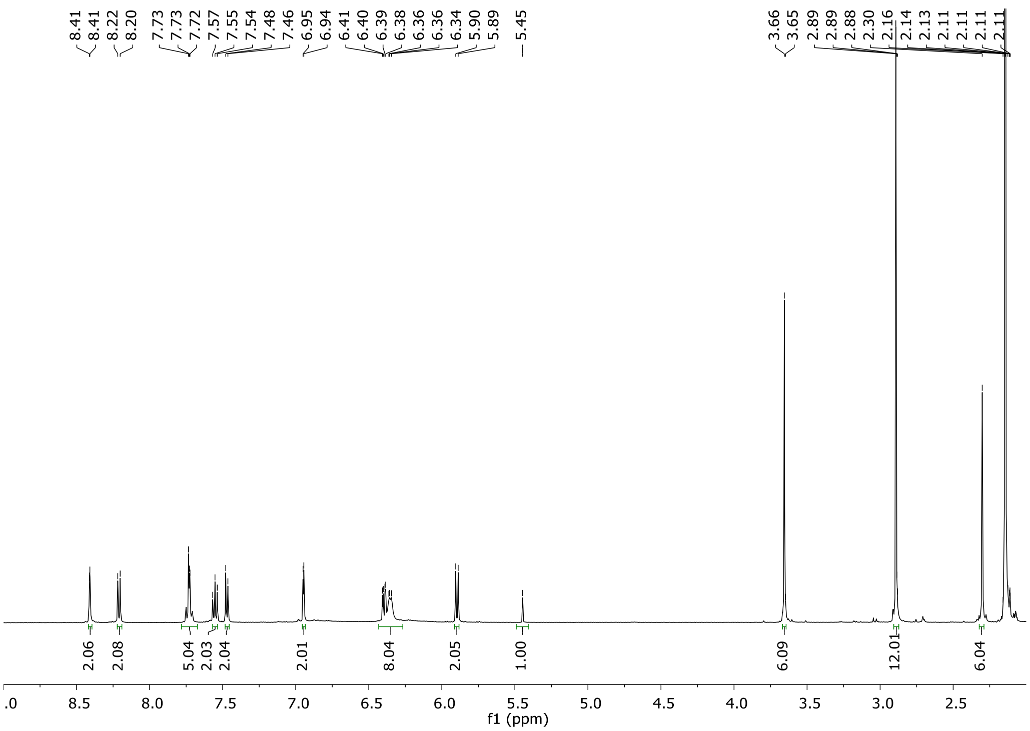

Supplementary Figure S17. ^{1}H NMR spectrum of [Ir(3-MeO-5-Me-ppy)$_2$(4-NMe$_2$Ph-BIAN)](PF$_6$) (**3**) in CD_3CN.

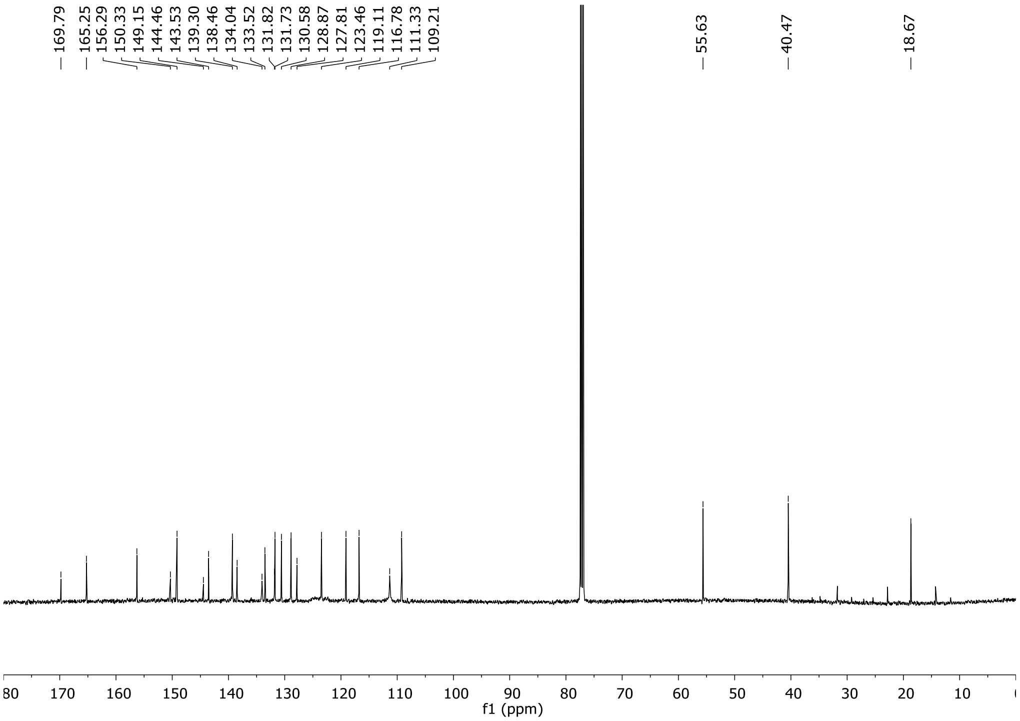

Supplementary Figure S18. ^{13}C NMR spectrum of [Ir(3-MeO-5-Me-ppy)$_2$(4-NMe$_2$Ph-BIAN)](PF$_6$) (**3**) in $CDCl_3$.

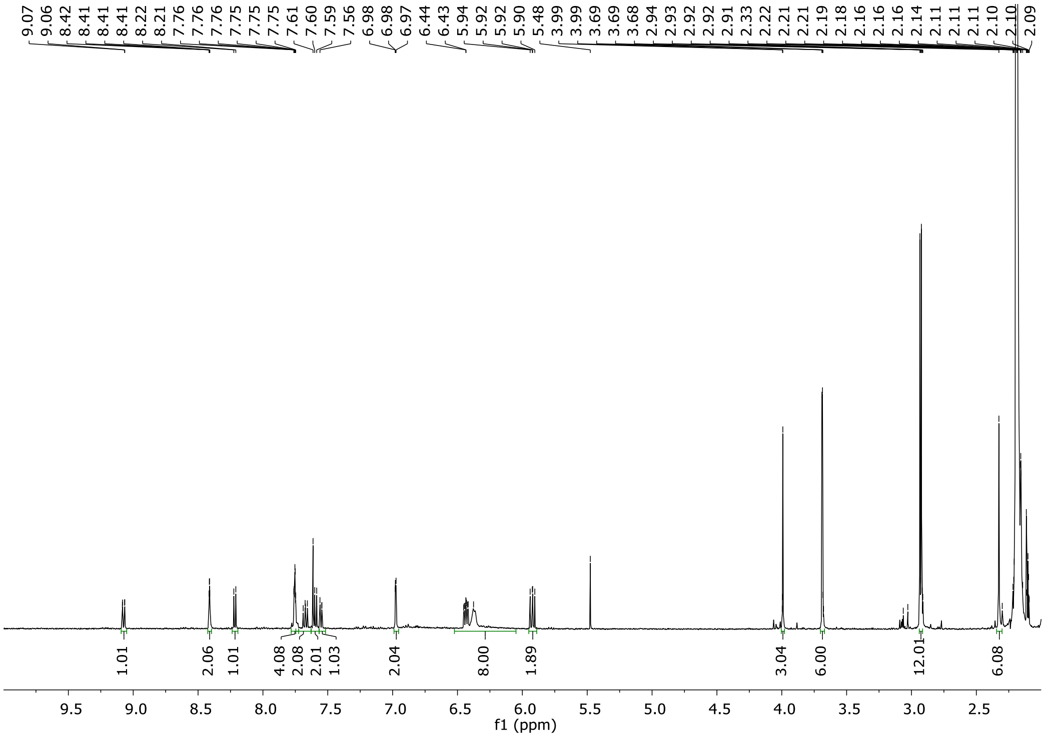

Supplementary Figure S19. ^{1}H NMR spectrum of [Ir(3-MeO-5-Me-ppy)$_2$(4-NMe$_2$Ph-BIAN-CO_2Me)](PF_6) (**4**) in CD_3CN.

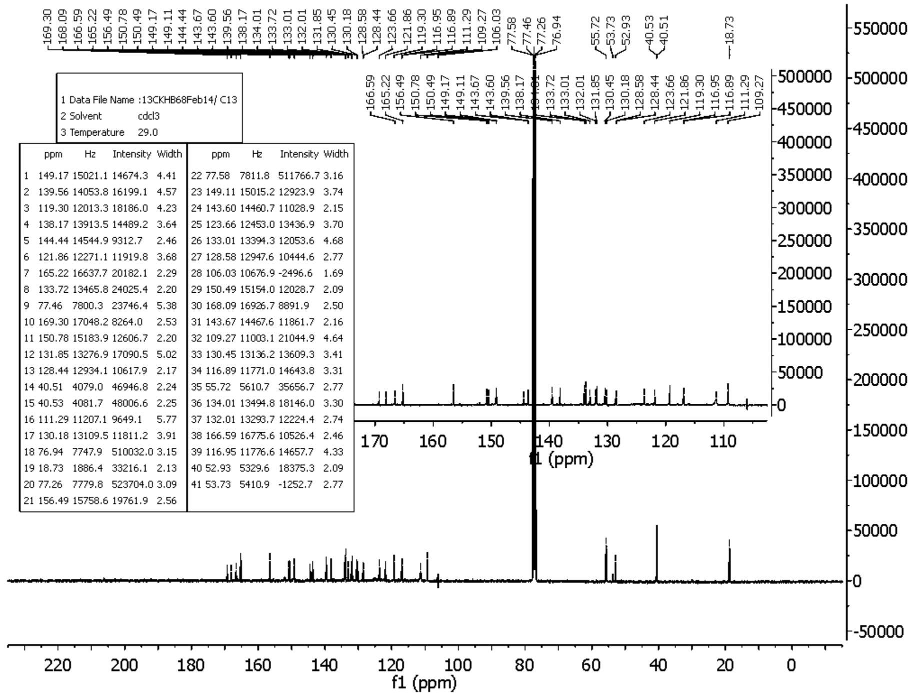

Supplementary Figure S20. ^{13}C NMR spectrum of [Ir(3-MeO-5-Me-ppy)$_2$(4-NMe$_2$Ph-BIAN-CO_2Me)](PF_6) (**4**) in $CDCl_3$.

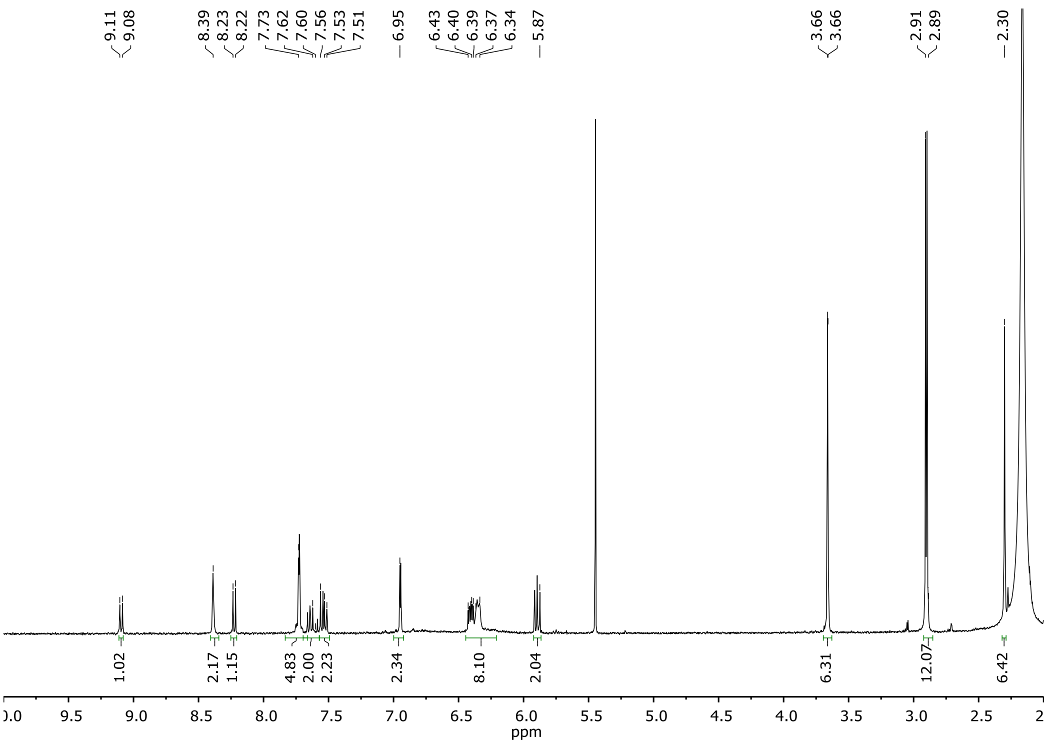

Supplementary Figure S21. ^{1}H NMR spectrum of [Ir(3-MeO-5-Me-ppy)$_2$(4-NMe$_2$Ph-BIAN-CO$_2$H)](PF$_6$) (**5**) in CD_3CN.

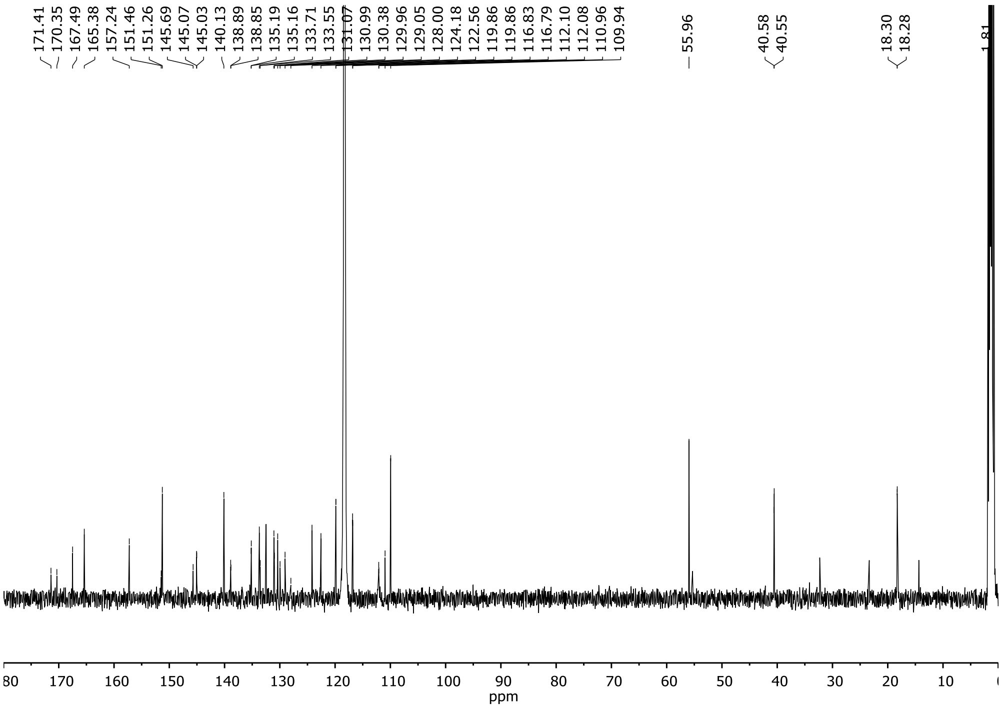

Supplementary Figure S22. ^{13}C NMR spectrum of [Ir(3-MeO-5-Me-ppy)$_2$(4-NMe$_2$Ph-BIAN-CO$_2$H)](PF$_6$) (**5**) in CD_3CN.

DFT Calculations:

All calculations were performed with the Gaussian09, revision D.01[16] suite of programmes employing the DFT method, the Becke three-parameter hybrid functional,[17] and the Lee-Yang-Parr's gradient-corrected correlation functional (B3LYP).[18] Singlet and triplet ground state geometry optimisations and single point energy calculations for [**1**]$^+$-[**5**]$^+$ were carried out at the (R)B3LYP and (U)B3LYP levels, using their respective crystallographic structures as starting points. All elements except iridium were assigned the 6-31G(d,p) basis set.[19] The double-ζ quality SBKJC VDZ ECP basis set[20] with an effective core potential was employed for the Ir(III) center. Vertical electronic excitations based on (R)B3LYP-optimised geometries were computed for [**1**]$^+$-[**5**]$^+$ using the TD-DFT formalism[21,22] in acetonitrile using conductor-like polarisable continuum model (CPCM).[23-25] Vibrational frequency calculations were performed to ensure that the optimised geometries represent the local minima and only positive eigenvalues were obtained. The electronic distribution and localisation of the singlet excited states were visualised using the electron density difference maps (ED-DMs).[26] *GaussSum 2.2* and *Chemissian* v3.8[27] were employed to visualise the absorption spectra (simulated using a Gaussian distribution with a full-width at half maximum (fwhm) set to 3000 cm^{-1}) and to calculate the fractional contributions of various groups to each molecular orbital. All calculated structures and Kohn-Sham orbitals were visualised with ChemCraft.[28]

Supplementary Table S1. Selected transitions from TD-DFT calculations of [**1**]$^+$ in the singlet ground state (B3LYP/SBKJC-VDZ[Ir]6-31G**[C,H,N], CPCM (MeCN)).

State	λ_{abs}/nm (TD-DFT)	λ_{abs}/nm (ε × 10^4 M^{-1}cm^{-1}) [expt.]	f (TD-DFT)	Major transition(s)	Character
73	260	258 (12.1)	0.2045	H-2->L+8 (26%), H-1->L+9 (12%), H->L+10 (45%)	BIAN(π) to ppy(π*) + BIAN(π) to BIAN(π*) (almost equal contributions)
42	303	310 (4.02)	0.1818	H-6->L+3 (81%)	ppy(π) to ppy(π*) (major) + Ir(dπ) to ppy(π*) (minor)
10	420	396 (1.95)	0.2426	H-6->L (55%), H-2->L+1 (33%)	ppy(π) to BIAN(π*) (major) + Ir(dπ) to BIAN(π*) (minor)
3	608	576 (1.42)	0.1672	H-2->L (94%)	ppy(π) to BIAN(π*) (major) + Ir(dπ) to BIAN(π*) (minor)
1	712	675 (0.71)	0.4242	H->L (94%)	BIAN(π) to BIAN(π*)

Supplementary Table S2. Selected transitions from TD-DFT calculations of [**2**]$^+$ in the singlet ground state (B3LYP/SBKJC-VDZ[Ir]6-31G**[C,H,N,O], CPCM (MeCN)).

State	λ_{abs}/nm (TD-DFT)	λ_{abs}/nm (ε × 10^4 $M^{-1}cm^{-1}$) [expt.]	f (TD-DFT)	Major transition(s)	Character
65	272	261 (9.99)	0.1851	H-8->L+2 (78%)	BIAN(π) to 4-OMeppy(π*) (major) + Ir(dπ) to 4-OMeppy(π*) (minor)
39	312	310 (4.62)	0.2738	H-5->L+2 (52%), H-4->L+2 (25%)	BIAN(π) to 4-OMeppy(π*) (major) + Ir(dπ) to 4-OMeppy(π*) (minor) + 4-OMeppy(π) to 4-OMeppy(π*) (minor)
10	424	390 (2.15)	0.3694	H-6->L (87%)	Ir(dπ) to BIAN(π*) (major) + 4-OMeppy(π) to BIAN(π*) (minor)
4	547	570 (1.43)	0.0057	H-3->L (99%)	4-OMeppy(π) to BIAN(π*) (major) + Ir(dπ) to BIAN(π*) (minor)
2	641	665 (0.78)	0.2405	H-2->L (87%), H->L (11%)	4-OMeppy(π) to BIAN(π*) (major) + Ir(dπ) to BIAN(π*) (minor) + BIAN(π) to BIAN(π*) (minor)

Supplementary Table S3. Selected transitions from TD-DFT calculations of [**3**]$^+$ in the singlet ground state (B3LYP/SBKJC-VDZ[Ir]6-31G**[C,H,N,O], CPCM (MeCN)).

State	λ_{abs}/nm (TD-DFT)	λ_{abs}/nm (ε × 10^4 $M^{-1}cm^{-1}$) [expt.]	f (TD-DFT)	Major transition(s)	Character
74	266	262 (10.8)	0.3317	H-5->L+5 (78%)	3-OMeppy(π) to 3-OMeppy(π*) (major) + Ir(dπ) to 3-OMeppy(π*) (minor)
47	297	312 (3.18)	0.2662	H-6->L+2 (48%), H-3->L+5 (20%)	3-OMeppy(π) to 3-OMeppy(π*) (major) + BIAN(π) to 3-OMeppy(π*) (minor)
17	400	409 (1.66)	0.061	H-8->L (14%), H-7->L (70%)	3-OMeppy(π) to BIAN(π*) (major) + Ir(dπ) to BIAN(π*) (minor)
9	443	432 (1.59)	0.2654	H-5->L (81%)	3-OMeppy(π) to BIAN(π*) (major) + Ir(dπ) to BIAN(π*) (minor) + 3-OMeppy(π) to 3-OMeppy(π*) (minor) + Ir(dπ) to 3-OMeppy(π*) (minor)
3	601	585 (1.11)	0.0517	H-2->L (95%),	BIAN(π) to BIAN(π*) (major) + 3-OMeppy(π) to 3-OMeppy(π*) (major) + BIAN(π) to 3-OMeppy(π*) (minor)

Supplementary Table S4. Selected transitions from TD-DFT calculations of [**4**]$^+$ in the singlet ground state (B3LYP/SBKJC-VDZ[Ir]6-31G**[C,H,N,O], CPCM (MeCN)).

State	λ_{abs}/nm (TD-DFT)	λ_{abs}/nm (ε × 10^4 $M^{-1}cm^{-1}$) [expt.]	f (TD-DFT)	Major transition(s)	Character
79	267	264 (11.2)	0.3659	H-5->L+5 (68%)	3-OMeppy(π) to 3-OMeppy(π*) (major) + Ir(dπ) to 3-OMeppy(π*) (minor)
46	306	309 (4.48)	0.0861	H-7->L+3 (12%), H-5->L+3 (65%), H-4->L+3 (10%)	3-OMeppy(π) to 3-OMeppy(π*) (major) + Ir(dπ) to 3-OMeppy(π*) (minor) + BIAN(π) to 3-OMeppy(π*) (minor)
11	435	416 (1.85)	0.0886	H-1->L+2 (94%)	3-OMeppy(π) to 3-OMeppy(π*) (major) + Ir(dπ) to 3-OMeppy(π*) (minor)
4	578	590 (1.57)	0.1237	H->L+1 (90%)	BIAN(π) to BIAN(π*)

Supplementary Table S5. Selected transitions from TD-DFT calculations of [**5**]$^+$ in the singlet ground state (B3LYP/SBKJC-VDZ[Ir]6-31G**[C,H,N,O], CPCM (MeCN)).

State	λ_{abs}/nm (TD-DFT)	λ_{abs}/nm (ε × 10^4 $M^{-1}cm^{-1}$) [expt.]	f (TD-DFT)	Major transition(s)	Character
78	267	262 (10.2)	0.3906	H-5->L+5 (72%)	3-OMeppy(π) to 3-OMeppy(π*) (major) + Ir(dπ) to 3-OMeppy(π*) (minor)
55	295	310 (3.37)	0.2366	H-7->L+2 (31%), H-1->L+7 (16%), H-1->L+8 (10%)	3-OMeppy(π) to 3-OMeppy(π*) (major) + Ir(dπ) to 3-OMeppy(π*) (minor) + 3-OMeppy(π) to BIAN(π*) (minor) + Ir(dπ) to BIAN(π*) (minor)
11	435	427 (1.82)	0.0898	H-1->L+2 (94%)	3-OMeppy(π) to 3-OMeppy(π*) (major) + Ir(dπ) to 3-OMeppy(π*) (minor)
4	587	604 (1.41)	0.1402	H->L+1 (96%)	BIAN(π) to BIAN(π*)

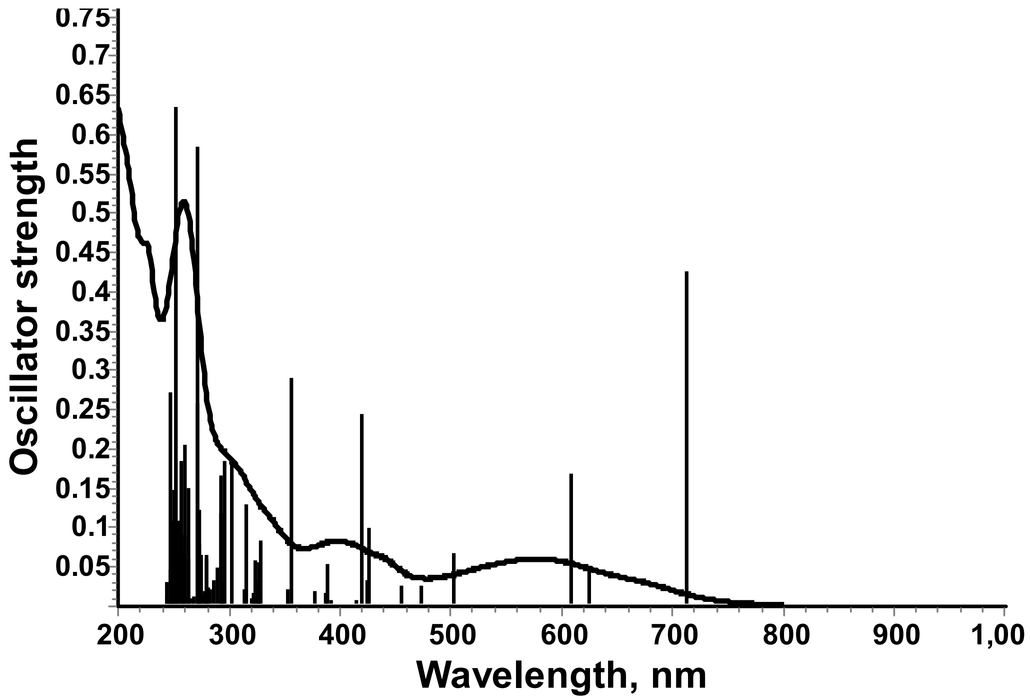

Supplementary Figure S23. Overlay of experimental UV-vis absorption spectra (curved line) of **1** with their predicted transitions (vertical bars) calculated by singlet TD-DFT at room temperature in MeCN.

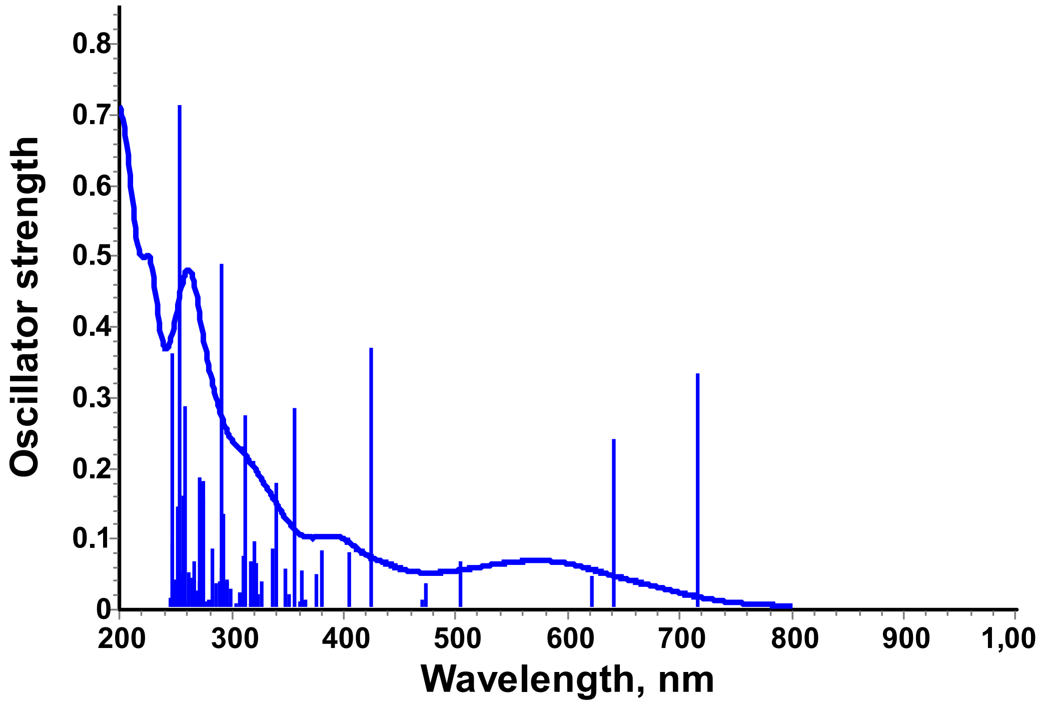

Supplementary Figure S24. Overlay of experimental UV-vis absorption spectra (curved line) of **2** with their predicted transitions (vertical bars) calculated by singlet TD-DFT at room temperature in MeCN.

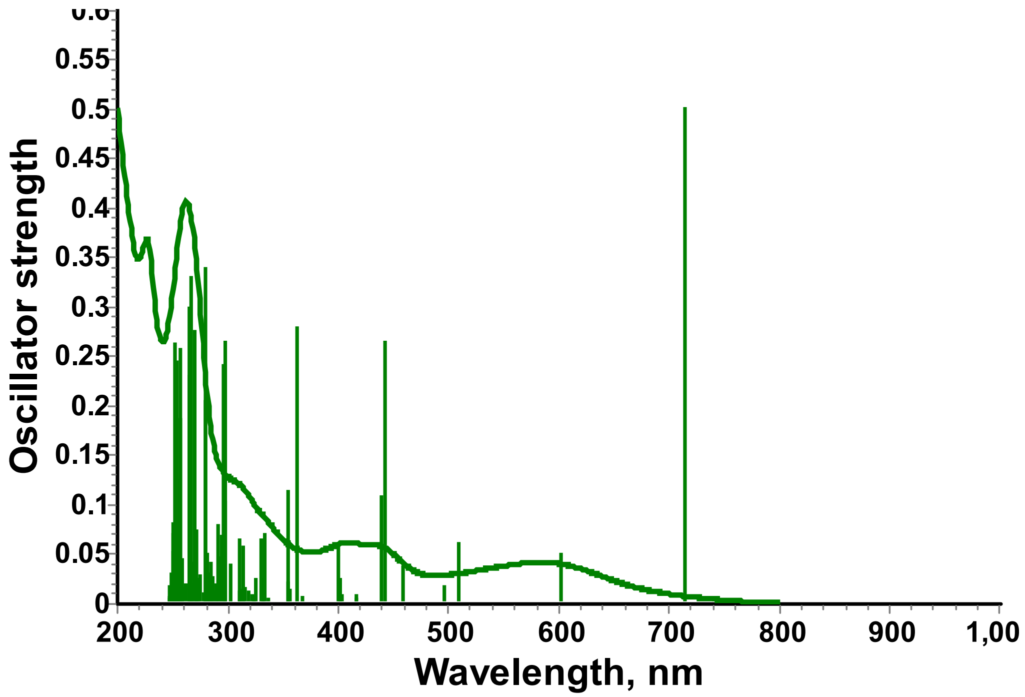

Supplementary Figure S25. Overlay of experimental UV-vis absorption spectra (curved line) of **3** with their predicted transitions (vertical bars) calculated by singlet TD-DFT at room temperature in MeCN.

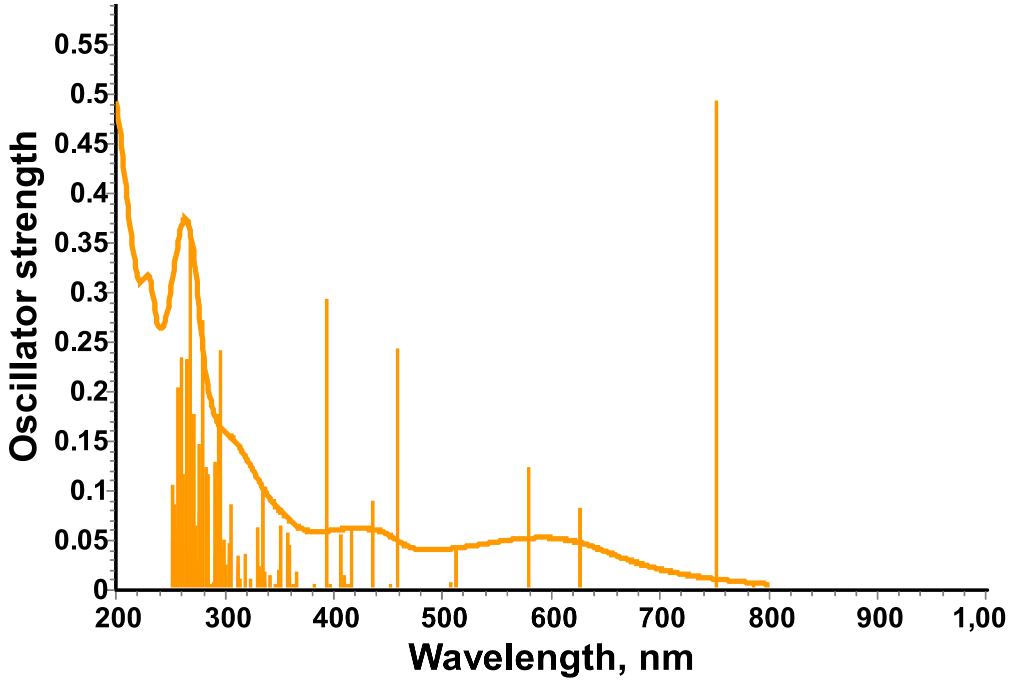

Supplementary Figure S26, Overlay of experimental UV-vis absorption spectra (curved line) of **4** with their predicted transitions (vertical bars) calculated by singlet TD-DFT at room temperature in MeCN.

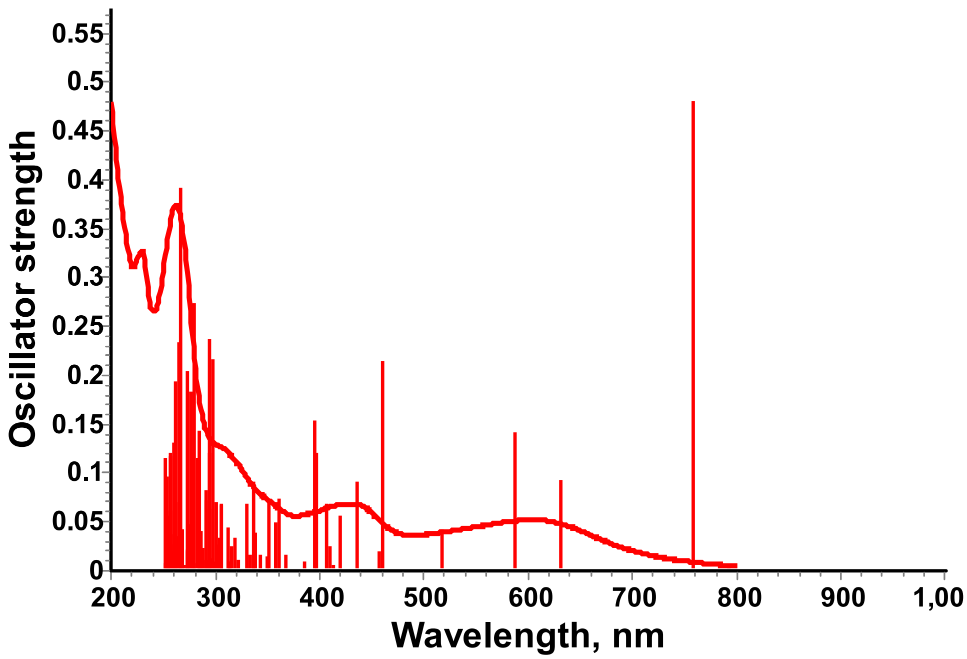

Supplementary Figure S27. Overlay of experimental UV-vis absorption spectra (curved line) of **5** with their predicted transitions (vertical bars) calculated by singlet TD-DFT at room temperature in MeCN.

Supplementary Table S6. Optimised atomic coordinates obtained from DFT calculations of [**1**]$^+$.

Center Number	Atomic Number	Atomic Type	Coordinates (Angstroms) X	Y	Z
1	77	0	-0.000016	-0.877141	0.000039
2	7	0	1.342294	0.897630	-0.264978
3	1	0	1.130262	-2.869833	-2.322171
4	6	0	1.669443	-3.078700	-1.402803
5	6	0	1.371194	-2.355783	-0.236752
6	6	0	2.656583	-4.067489	-1.412024
7	6	0	2.100119	-2.681816	0.938599
8	6	0	3.374137	-4.367707	-0.248366
9	6	0	1.746062	-1.913724	2.134104
10	6	0	3.094127	-3.675356	0.925824
11	1	0	4.141324	-5.135955	-0.257902
12	6	0	2.322572	-2.057576	3.404100
13	6	0	0.351057	-0.211866	2.964987
14	6	0	1.896067	-1.261698	4.459300
15	1	0	3.103599	-2.792113	3.557908
16	6	0	0.891069	-0.316487	4.238403
17	1	0	-0.428173	0.504306	2.737024
18	1	0	2.341928	-1.374311	5.442220

19	7	0	0.757195	-0.988910	1.941156
20	1	0	-1.130588	-2.869287	2.322533
21	6	0	-1.669766	-3.078243	1.403185
22	6	0	-1.371391	-2.355570	0.237021
23	6	0	-2.657032	-4.066909	1.412538
24	6	0	-2.100316	-2.681703	-0.938304
25	6	0	-3.374582	-4.367235	0.248907
26	6	0	-3.094448	-3.675116	-0.925392
27	6	0	-1.746146	-1.913833	-2.133918
28	1	0	-4.141865	-5.135385	0.258546
29	6	0	-2.322630	-2.057812	-3.403909
30	6	0	-0.350971	-0.212206	-2.964992
31	6	0	-1.896022	-1.262115	-4.459207
32	1	0	-3.103721	-2.792297	-3.557641
33	6	0	-0.890954	-0.316957	-4.238411
34	1	0	0.428301	0.503942	-2.737093
35	1	0	-2.341865	-1.374827	-5.442125
36	7	0	-0.757201	-0.989079	-1.941072
37	1	0	-2.867739	-4.607779	2.331809
38	1	0	-3.650670	-3.913552	-1.827334
39	1	0	-0.528856	0.327391	-5.030801
40	1	0	0.529043	0.327994	5.030718
41	1	0	3.650352	-3.913707	1.827788
42	1	0	2.867191	-4.608546	-2.331208
43	7	0	-1.342108	0.897602	0.264911
44	6	0	2.758072	0.811703	-0.323770
45	6	0	3.382050	0.096529	-1.357144
46	6	0	3.563779	1.356703	0.688813
47	6	0	4.763037	-0.016340	-1.416338
48	1	0	2.778272	-0.360306	-2.132882
49	6	0	4.944672	1.223359	0.656960
50	1	0	3.098440	1.869417	1.524874
51	6	0	5.592125	0.549526	-0.411961
52	1	0	5.200945	-0.557228	-2.245561
53	1	0	5.521871	1.635891	1.474644
54	6	0	-2.757939	0.811791	0.323655
55	6	0	-3.382057	0.097033	1.357211
56	6	0	-3.563478	1.356506	-0.689200
57	6	0	-4.763067	-0.015729	1.416285
58	1	0	-2.778397	-0.359573	2.133175
59	6	0	-4.944392	1.223276	-0.657444
60	1	0	-3.098002	1.868920	-1.525367
61	6	0	-5.592004	0.549860	0.411636
62	1	0	-5.201098	-0.556290	2.245655
63	1	0	-5.521472	1.635547	-1.475342
64	6	0	0.737893	2.043325	-0.118735

65	6	0	1.172488	3.449091	-0.202568
66	6	0	0.000063	4.228746	0.000046
67	6	0	2.366229	4.101644	-0.464541
68	6	0	-1.172337	3.449053	0.202634
69	6	0	0.000034	5.638923	0.000051
70	6	0	2.378430	5.519630	-0.500618
71	1	0	3.289018	3.559967	-0.627752
72	6	0	-2.366117	4.101551	0.464579
73	6	0	-1.243183	6.276355	0.264739
74	6	0	1.243225	6.276410	-0.264641
75	1	0	3.318211	6.022226	-0.706546
76	6	0	-2.378369	5.519533	0.500681
77	1	0	-3.288885	3.559828	0.627751
78	1	0	-1.297487	7.361004	0.281063
79	1	0	1.297486	7.361061	-0.280951
80	1	0	-3.318171	6.022098	0.706592
81	6	0	-0.737703	2.043307	0.118761
82	6	0	7.590160	-0.338741	-1.524396
83	1	0	7.346812	0.061226	-2.516236
84	1	0	8.672113	-0.296290	-1.403300
85	1	0	7.284306	-1.393379	-1.498345
86	6	0	-7.780067	0.970840	-0.614969
87	1	0	-8.831623	0.824660	-0.370183
88	1	0	-7.613373	2.045803	-0.752872
89	1	0	-7.578816	0.469353	-1.571672
90	7	0	6.960812	0.440911	-0.466964
91	7	0	-6.960724	0.441351	0.466542
92	6	0	7.780316	0.970878	0.614197
93	1	0	8.831824	0.824062	0.369597
94	1	0	7.614052	2.046012	0.751361
95	1	0	7.578789	0.470126	1.571215
96	6	0	-7.590145	-0.338613	1.523699
97	1	0	-7.347199	0.061304	2.515658
98	1	0	-8.672076	-0.296450	1.402303
99	1	0	-7.283988	-1.393165	1.497617

Supplementary Table S7. Optimised atomic coordinates obtained from DFT calculations of [**2**]$^+$.

Center Number	Atomic Number	Atomic Type	Coordinates (Angstroms) X	Y	Z
1	77	0	-0.000010	-0.608058	-0.000067
2	7	0	1.357243	1.162958	-0.157204
3	1	0	1.282574	-2.588739	-2.216710

4	6	0	1.761645	-2.805556	-1.269939
5	6	0	1.386345	-2.086925	-0.124712
6	6	0	2.752533	-3.794973	-1.202727
7	6	0	2.021778	-2.399829	1.105702
8	6	0	3.385147	-4.086881	0.020343
9	6	0	1.576024	-1.632601	2.263219
10	6	0	3.017566	-3.393316	1.161090
11	1	0	4.149505	-4.856361	0.046441
12	6	0	2.052250	-1.762332	3.578001
13	6	0	0.108066	0.058715	2.989143
14	6	0	1.538777	-0.966921	4.589423
15	1	0	2.826089	-2.487794	3.798610
16	6	0	0.538068	-0.021405	4.309258
17	1	0	-0.659420	0.765992	2.697758
18	1	0	1.913041	-1.074379	5.603711
19	7	0	0.598527	-0.715921	1.999243
20	1	0	-1.282529	-2.589504	2.215918
21	6	0	-1.761743	-2.805901	1.269123
22	6	0	-1.386498	-2.086864	0.124139
23	6	0	-2.752760	-3.795184	1.201659
24	6	0	-2.022096	-2.399213	-1.106329
25	6	0	-3.385573	-4.086491	-0.021460
26	6	0	-3.018034	-3.392531	-1.161981
27	6	0	-1.576301	-1.631654	-2.263613
28	1	0	-4.150034	-4.855865	-0.047782
29	6	0	-2.052637	-1.760855	-3.578405
30	6	0	-0.108029	0.059580	-2.989078
31	6	0	-1.539071	-0.965212	-4.589602
32	1	0	-2.826611	-2.486115	-3.799206
33	6	0	-0.538171	-0.019980	-4.309188
34	1	0	0.659668	0.766559	-2.697528
35	1	0	-1.913414	-1.072276	-5.603903
36	7	0	-0.598605	-0.715258	-1.999396
37	8	0	-3.176435	-4.535188	2.265039
38	1	0	-3.511748	-3.631058	-2.099748
39	6	0	0.044702	0.870351	-5.375993
40	6	0	-0.044533	0.868784	5.376327
41	1	0	3.511140	-3.632268	2.098821
42	8	0	3.176227	-4.534626	-2.266391
43	7	0	-1.357258	1.163071	0.157529
44	6	0	2.773638	1.075674	-0.117316
45	6	0	3.463611	0.355089	-1.103713
46	6	0	3.510692	1.625547	0.943534
47	6	0	4.845505	0.242677	-1.072553
48	1	0	2.909764	-0.108013	-1.912032
49	6	0	4.890899	1.492655	1.002285

50	1	0	2.991455	2.142293	1.744669
51	6	0	5.606824	0.814537	-0.019195
52	1	0	5.337012	-0.303370	-1.867542
53	1	0	5.414110	1.909641	1.853264
54	6	0	-2.773637	1.075851	0.117800
55	6	0	-3.463497	0.355052	1.104124
56	6	0	-3.510827	1.625907	-0.942863
57	6	0	-4.845399	0.242695	1.073138
58	1	0	-2.909550	-0.108289	1.912237
59	6	0	-4.891046	1.493069	-1.001445
60	1	0	-2.991681	2.142713	-1.744017
61	6	0	-5.606865	0.814886	0.020067
62	1	0	-5.336791	-0.303595	1.868030
63	1	0	-5.414336	1.910113	-1.852346
64	6	0	0.744637	2.309409	-0.061362
65	6	0	1.184370	3.715260	-0.114670
66	6	0	0.000143	4.495003	0.000200
67	6	0	2.394121	4.367804	-0.288125
68	6	0	-1.184158	3.715376	0.115051
69	6	0	0.000219	5.905230	0.000209
70	6	0	2.408990	5.785805	-0.323670
71	1	0	3.326347	3.826038	-0.382905
72	6	0	-2.393839	4.368051	0.288521
73	6	0	-1.259084	6.542812	0.172541
74	6	0	1.259592	6.542681	-0.172120
75	1	0	3.361395	6.288320	-0.460207
76	6	0	-2.408560	5.786050	0.324083
77	1	0	-3.326123	3.826386	0.383309
78	1	0	-1.314487	7.627458	0.184894
79	1	0	1.315102	7.627322	-0.184458
80	1	0	-3.360912	6.288662	0.460628
81	6	0	-0.744569	2.309474	0.061685
82	6	0	7.674519	-0.069627	-1.000131
83	1	0	7.494482	0.330695	-2.005340
84	1	0	8.746290	-0.024258	-0.809643
85	1	0	7.370740	-1.125176	-0.995022
86	6	0	-7.723917	1.241878	-1.144368
87	1	0	-8.789026	1.092438	-0.970138
88	1	0	-7.550735	2.317910	-1.265575
89	1	0	-7.459099	0.745690	-2.088217
90	7	0	6.976439	0.707003	0.015463
91	7	0	-6.976528	0.707631	-0.014269
92	6	0	7.723749	1.241710	1.145394
93	1	0	8.788821	1.091353	0.971752
94	1	0	7.551251	2.317968	1.265739
95	1	0	7.458194	0.746422	2.089493

96	6	0	-7.674375	-0.070044	1.000689
97	1	0	-7.493558	0.328944	2.006269
98	1	0	-8.746241	-0.023870	0.810911
99	1	0	-7.371125	-1.125751	0.994089
100	1	0	0.827262	1.516956	-4.971436
101	1	0	0.479711	0.278426	-6.188371
102	1	0	-0.726724	1.509654	-5.819142
103	1	0	-0.826382	1.516285	4.971833
104	1	0	-0.480405	0.276734	6.188161
105	1	0	0.727257	1.507123	5.820219
106	6	0	-2.576314	-4.308029	3.538314
107	6	0	2.577171	-4.306268	-3.539613
108	1	0	2.744669	-3.279240	-3.884105
109	1	0	-2.743490	-3.281294	3.883777
110	1	0	3.061276	-5.000703	-4.227235
111	1	0	1.500125	-4.508383	-3.518383
112	1	0	-3.060093	-5.003065	4.225541
113	1	0	-1.499340	-4.510289	3.515998

Supplementary Table S8. Optimised atomic coordinates obtained from DFT calculations of [**3**]$^{+}$.

Center Number	Atomic Number	Atomic Type	Coordinates (Angstroms) X	Y	Z
1	77	0	0.013279	-0.547317	0.109542
2	7	0	-1.308052	1.253468	0.169766
3	1	0	-1.639866	-2.521745	2.135733
4	6	0	-1.996420	-2.686795	1.123272
5	6	0	-1.440968	-1.967766	0.058606
6	6	0	-3.021445	-3.623405	0.938770
7	6	0	-1.952626	-2.247394	-1.236517
8	6	0	-3.519871	-3.869453	-0.347323
9	6	0	-1.331248	-1.501677	-2.333753
10	6	0	-2.979160	-3.178884	-1.435031
11	6	0	-1.650910	-1.619686	-3.693232
12	6	0	0.310548	0.087664	-2.875905
13	6	0	-0.970806	-0.868125	-4.639092
14	1	0	-2.431583	-2.303877	-4.003109
15	6	0	0.044659	0.014541	-4.240313
16	1	0	1.086130	0.740776	-2.497878
17	1	0	-1.222872	-0.965749	-5.691199
18	7	0	-0.348019	-0.637179	-1.949529
19	1	0	1.620774	-2.493952	-1.976288
20	6	0	1.896848	-2.771612	-0.963091

21	6	0	1.347393	-2.082986	0.124210
22	6	0	2.808163	-3.822881	-0.798283
23	6	0	1.749838	-2.508124	1.419484
24	6	0	3.190783	-4.221325	0.489478
25	6	0	2.658086	-3.558873	1.598781
26	6	0	1.178253	-1.748680	2.535306
27	6	0	1.428943	-1.968346	3.896621
28	6	0	-0.220893	0.052989	3.102873
29	1	0	2.087519	-2.774321	4.197260
30	6	0	-0.012652	-0.112967	4.469377
31	1	0	-0.865869	0.840702	2.733111
32	7	0	0.344078	-0.733141	2.163941
33	1	0	3.206292	-4.321085	-1.674963
34	1	0	2.974303	-3.884193	2.584879
35	6	0	-0.674860	0.792944	5.474499
36	6	0	0.817430	0.845445	-5.231510
37	1	0	-3.378654	-3.392076	-2.421419
38	1	0	-3.418742	-4.150236	1.798911
39	7	0	1.430166	1.209332	0.038118
40	6	0	-2.720323	1.220132	0.018222
41	6	0	-3.518332	0.485808	0.907579
42	6	0	-3.343680	1.848590	-1.071662
43	6	0	-4.895349	0.427247	0.750746
44	1	0	-3.055743	-0.031164	1.739932
45	6	0	-4.717282	1.771720	-1.255599
46	1	0	-2.740050	2.385678	-1.796864
47	6	0	-5.542127	1.072536	-0.336023
48	1	0	-5.472293	-0.133501	1.474925
49	1	0	-5.148592	2.249790	-2.125862
50	6	0	2.829594	1.137195	-0.202389
51	6	0	3.414108	1.801312	-1.293796
52	6	0	3.654129	0.337289	0.602724
53	6	0	4.769966	1.684906	-1.566643
54	1	0	2.796367	2.409577	-1.947600
55	6	0	5.016003	0.238277	0.358862
56	1	0	3.227960	-0.191659	1.445644
57	6	0	5.620538	0.908318	-0.737147
58	1	0	5.166940	2.195807	-2.434640
59	1	0	5.614297	-0.371514	1.023819
60	6	0	-0.664441	2.385892	0.196006
61	6	0	-1.079800	3.791241	0.349765
62	6	0	0.124910	4.543736	0.425401
63	6	0	-2.287065	4.450862	0.513878
64	6	0	1.298568	3.751410	0.289565
65	6	0	0.153454	5.934168	0.660201
66	6	0	-2.277273	5.851184	0.740386

67	1	0	-3.233662	3.928112	0.466903
68	6	0	2.533393	4.370607	0.398722
69	6	0	-1.103576	6.582331	0.808466
70	1	0	-3.227905	6.360429	0.863666
71	6	0	2.580930	5.769587	0.627739
72	1	0	3.459474	3.817879	0.307631
73	1	0	-1.137597	7.653565	0.984681
74	1	0	3.552805	6.246291	0.707946
75	6	0	0.829481	2.360743	0.145434
76	6	0	-7.714254	0.205624	0.406751
77	1	0	-7.612793	0.541382	1.445902
78	1	0	-8.763088	0.299226	0.126908
79	1	0	-7.442556	-0.857977	0.364641
80	6	0	7.548508	1.466965	-2.146932
81	1	0	8.623341	1.289105	-2.158276
82	1	0	7.130356	1.089599	-3.090056
83	1	0	7.386767	2.551330	-2.113279
84	8	0	-4.520034	-4.758730	-0.641471
85	6	0	-5.097948	-5.494252	0.431170
86	1	0	-5.569196	-4.832112	1.167854
87	1	0	-5.860707	-6.132397	-0.017074
88	1	0	-4.354717	-6.122098	0.937719
89	7	0	-6.906502	1.015055	-0.495378
90	7	0	6.967481	0.804999	-0.987091
91	6	0	7.800642	-0.060113	-0.163072
92	1	0	7.479082	-1.108994	-0.212193
93	1	0	8.830044	-0.004657	-0.515540
94	1	0	7.787980	0.250503	0.889054
95	6	0	-7.530645	1.618489	-1.664744
96	1	0	-8.610882	1.492421	-1.598031
97	1	0	-7.320789	2.693396	-1.719825
98	1	0	-7.189947	1.156498	-2.601701
99	6	0	0.837290	-1.160333	4.856033
100	1	0	1.034730	-1.338179	5.909284
101	8	0	4.071089	-5.234691	0.764981
102	6	0	4.638970	-5.943424	-0.330957
103	1	0	5.300162	-6.693589	0.104940
104	1	0	3.869350	-6.445290	-0.930027
105	1	0	5.223709	-5.281312	-0.981205
106	6	0	1.436848	6.539696	0.749414
107	1	0	1.515036	7.609024	0.922696
108	1	0	-1.306815	1.537995	4.985184
109	1	0	-1.300148	0.218839	6.166523
110	1	0	0.071486	1.322911	6.075925
111	1	0	1.552959	1.481206	-4.732768
112	1	0	1.349298	0.208153	-5.946126

113	1	0	0.146972	1.490423	-5.809361

Supplementary Table S9. Optimised atomic coordinates obtained from DFT calculations of [**4**]$^{+}$.

Center Number	Atomic Number	Atomic Type	Coordinates (Angstroms) X	Y	Z
1	77	0	-0.967902	-0.417449	0.120356
2	7	0	0.029509	1.580950	0.057134
3	1	0	-3.346550	0.201047	2.284814
4	6	0	-3.721693	0.413911	1.288159
5	6	0	-2.898075	0.220877	0.173131
6	6	0	-5.034643	0.888012	1.173587
7	6	0	-3.463759	0.507867	-1.097347
8	6	0	-5.564082	1.180190	-0.090288
9	6	0	-2.588716	0.263282	-2.247029
10	6	0	-4.773700	0.985580	-1.226078
11	6	0	-2.923572	0.453416	-3.591869
12	6	0	-0.462592	-0.492351	-2.900494
13	6	0	-2.003999	0.160402	-4.590884
14	1	0	-3.905805	0.828252	-3.853053
15	6	0	-0.735175	-0.329616	-4.258769
16	1	0	0.495087	-0.874585	-2.570486
17	1	0	-2.271620	0.309135	-5.632915
18	7	0	-1.346153	-0.208340	-1.926496
19	1	0	-2.110573	-2.804360	-1.812200
20	6	0	-2.156510	-3.142713	-0.781106
21	6	0	-1.719035	-2.304065	0.250102
22	6	0	-2.659727	-4.427346	-0.536871
23	6	0	-1.821828	-2.811407	1.573978
24	6	0	-2.746070	-4.906097	0.777056
25	6	0	-2.324715	-4.092410	1.832450
26	6	0	-1.334878	-1.918508	2.629607
27	6	0	-1.321857	-2.196850	4.003042
28	6	0	-0.347662	0.170581	3.062473
29	1	0	-1.711601	-3.141437	4.362790
30	6	0	-0.305538	-0.045785	4.437107
31	1	0	0.025046	1.094223	2.636590
32	7	0	-0.845220	-0.724095	2.184235
33	1	0	-2.981651	-5.037880	-1.372873
34	1	0	-2.401207	-4.488399	2.840142
35	6	0	0.259411	0.990644	5.373137
36	6	0	0.301965	-0.673618	-5.297963
37	1	0	-5.210307	1.212493	-2.193549

38	1	0	-5.629226	1.023615	2.069901
39	7	0	1.236582	-0.881828	-0.076720
40	6	0	-0.645154	2.819435	-0.108405
41	6	0	-1.626449	3.231332	0.805504
42	6	0	-0.402671	3.624702	-1.233090
43	6	0	-2.296671	4.434541	0.639499
44	1	0	-1.852955	2.610482	1.664072
45	6	0	-1.088983	4.815008	-1.425621
46	1	0	0.319471	3.300982	-1.976341
47	6	0	-2.044535	5.271181	-0.479738
48	1	0	-3.029488	4.722918	1.382131
49	1	0	-0.887516	5.388001	-2.321653
50	6	0	1.797717	-2.155249	-0.349621
51	6	0	2.620599	-2.362722	-1.471238
52	6	0	1.481341	-3.263652	0.452002
53	6	0	3.119709	-3.620101	-1.775488
54	1	0	2.857379	-1.529918	-2.126417
55	6	0	2.002159	-4.518283	0.177757
56	1	0	0.844886	-3.131938	1.317352
57	6	0	2.837596	-4.740606	-0.949509
58	1	0	3.724212	-3.733779	-2.666187
59	1	0	1.752498	-5.335699	0.842087
60	6	0	1.331717	1.521263	0.029496
61	6	0	2.399689	2.532815	0.116603
62	6	0	3.629614	1.813329	0.152761
63	6	0	2.422846	3.908625	0.261154
64	6	0	3.441351	0.405479	0.055833
65	6	0	4.891857	2.431934	0.321852
66	6	0	3.674432	4.545722	0.431457
67	1	0	1.518424	4.502394	0.244406
68	6	0	4.545945	-0.421872	0.153043
69	6	0	6.022397	1.543454	0.383742
70	6	0	4.871436	3.850716	0.457379
71	1	0	3.694008	5.624877	0.547282
72	6	0	5.815657	0.168617	0.318106
73	1	0	4.466081	-1.499313	0.097813
74	1	0	5.798801	4.388941	0.590550
75	1	0	6.686921	-0.472094	0.391025
76	6	0	1.985406	0.185106	-0.023623
77	6	0	-3.744511	6.864080	0.291836
78	1	0	-3.356058	6.936273	1.314842
79	1	0	-4.122779	7.845899	0.008577
80	1	0	-4.588312	6.160664	0.296916
81	6	0	4.172098	-6.186958	-2.413713
82	1	0	4.492037	-7.227618	-2.450191
83	1	0	3.627264	-5.965284	-3.340962

84	1	0	5.070365	-5.558067	-2.387451
85	6	0	7.455033	1.957473	0.528250
86	8	0	8.352175	1.182394	0.814141
87	8	0	7.671958	3.261353	0.285239
88	6	0	9.040477	3.703732	0.402982
89	8	0	-6.830601	1.651210	-0.317190
90	6	0	-7.680507	1.851802	0.806820
91	1	0	-7.266902	2.595460	1.498995
92	1	0	-8.626553	2.220246	0.407855
93	1	0	-7.858038	0.916161	1.350914
94	7	0	-2.702757	6.466175	-0.645449
95	7	0	3.342615	-5.984124	-1.233127
96	6	0	2.988866	-7.126254	-0.400141
97	1	0	1.907102	-7.312932	-0.400336
98	1	0	3.485953	-8.015954	-0.784863
99	1	0	3.309829	-6.982250	0.638936
100	6	0	-2.476100	7.265169	-1.841954
101	1	0	-3.055336	8.185343	-1.771753
102	1	0	-1.419599	7.540247	-1.944475
103	1	0	-2.781231	6.738409	-2.756592
104	6	0	-0.814182	-1.268890	4.899852
105	1	0	-0.809092	-1.491163	5.963096
106	8	0	-3.222358	-6.141311	1.129651
107	6	0	-3.673965	-7.005542	0.092625
108	1	0	-4.008825	-7.919061	0.585838
109	1	0	-4.512345	-6.567088	-0.462194
110	1	0	-2.867930	-7.249873	-0.610044
111	1	0	1.218048	-0.091315	-5.153247
112	1	0	0.578194	-1.732201	-5.247030
113	1	0	-0.071833	-0.470486	-6.304099
114	1	0	0.613394	1.869684	4.829285
115	1	0	-0.495658	1.319031	6.095340
116	1	0	1.099883	0.583980	5.945513
117	1	0	9.412354	3.525211	1.413856
118	1	0	9.671807	3.175648	-0.314363
119	1	0	9.020375	4.770010	0.183943

Supplementary Table S10. Optimised atomic coordinates obtained from DFT calculations of [**5**]$^{+}$.

Center Number	Atomic Number	Atomic Type	Coordinates (Angstroms) X	Y	Z
1	77	0	-0.548431	-0.711965	0.123600
2	7	0	-0.637938	1.520501	0.077341

3	1	0	-2.959257	-1.303014	2.258681
4	6	0	-3.376868	-1.304903	1.256282
5	6	0	-2.548017	-1.079309	0.151250
6	6	0	-4.753236	-1.528830	1.124988
7	6	0	-3.165964	-1.104012	-1.127012
8	6	0	-5.341125	-1.536213	-0.146763
9	6	0	-2.269661	-0.886562	-2.265412
10	6	0	-4.541194	-1.323769	-1.272642
11	6	0	-2.639692	-0.875923	-3.614422
12	6	0	-0.038631	-0.501025	-2.891416
13	6	0	-1.683385	-0.674483	-4.601491
14	1	0	-3.676828	-1.027422	-3.888034
15	6	0	-0.340836	-0.482984	-4.253068
16	1	0	0.980044	-0.367563	-2.550120
17	1	0	-1.978306	-0.668383	-5.646742
18	7	0	-0.958863	-0.692243	-1.928716
19	1	0	-0.370258	-3.331608	-1.835008
20	6	0	-0.270134	-3.662947	-0.805623
21	6	0	-0.307729	-2.729374	0.235927
22	6	0	-0.100194	-5.034184	-0.573952
23	6	0	-0.178922	-3.238986	1.556729
24	6	0	0.029233	-5.511027	0.737093
25	6	0	-0.010401	-4.607301	1.802501
26	6	0	-0.191547	-2.232547	2.622581
27	6	0	-0.071270	-2.484380	3.995987
28	6	0	-0.306914	0.071577	3.074705
29	1	0	0.021537	-3.504564	4.348356
30	6	0	-0.187766	-0.111833	4.449574
31	1	0	-0.402224	1.066266	2.656718
32	7	0	-0.316310	-0.944065	2.187185
33	1	0	-0.073883	-5.714608	-1.417436
34	1	0	0.096509	-5.003094	2.807488
35	6	0	-0.185600	1.060802	5.395268
36	6	0	0.744147	-0.270347	-5.278467
37	1	0	-5.020935	-1.339320	-2.246169
38	1	0	-5.350113	-1.695532	2.014535
39	7	0	1.606839	-0.055539	-0.051861
40	6	0	-1.829032	2.277085	-0.085455
41	6	0	-2.880545	2.165445	0.836087
42	6	0	-2.016653	3.088771	-1.215754
43	6	0	-4.056068	2.882526	0.668557
44	1	0	-2.768234	1.522302	1.700935
45	6	0	-3.199948	3.787673	-1.408822
46	1	0	-1.232250	3.153169	-1.963658
47	6	0	-4.253161	3.722333	-0.459148
48	1	0	-4.832007	2.782295	1.416554

49	1	0	-3.309658	4.379100	-2.308701
50	6	0	2.712228	-0.898701	-0.326309
51	6	0	3.548526	-0.662584	-1.432875
52	6	0	2.955973	-2.040609	0.454524
53	6	0	4.595928	-1.516837	-1.739942
54	1	0	3.362290	0.192747	-2.075097
55	6	0	4.021010	-2.882804	0.177820
56	1	0	2.321365	-2.251341	1.305631
57	6	0	4.877431	-2.650920	-0.931953
58	1	0	5.192288	-1.308155	-2.618783
59	1	0	4.187915	-3.733462	0.825970
60	6	0	0.531329	2.095835	0.053610
61	6	0	0.977979	3.497035	0.141588
62	6	0	2.401698	3.460467	0.193522
63	6	0	0.332279	4.713550	0.270589
64	6	0	2.916750	2.136237	0.100529
65	6	0	3.206865	4.611958	0.369672
66	6	0	1.119099	5.876411	0.443022
67	1	0	-0.746152	4.796294	0.238813
68	6	0	4.282553	1.943749	0.210555
69	6	0	4.625209	4.377448	0.446053
70	6	0	2.502522	5.845587	0.488044
71	1	0	0.614556	6.832103	0.545299
72	6	0	5.107862	3.073083	0.383286
73	1	0	4.732571	0.961501	0.158432
74	1	0	3.051387	6.766269	0.623260
75	1	0	6.179624	2.933263	0.464321
76	6	0	1.748239	1.240692	0.007118
77	6	0	-6.521318	4.262708	0.305142
78	1	0	-6.226742	4.530204	1.327029
79	1	0	-7.340977	4.918080	0.012225
80	1	0	-6.896333	3.230193	0.317969
81	6	0	6.781800	-3.228563	-2.364999
82	1	0	7.567426	-3.982211	-2.402520
83	1	0	6.224882	-3.270004	-3.310282
84	1	0	7.260172	-2.244107	-2.293649
85	6	0	5.677332	5.427737	0.601896
86	8	0	6.852348	5.182523	0.815223
87	8	0	5.231834	6.692886	0.468408
88	1	0	6.000340	7.277422	0.588976
89	8	0	-6.673479	-1.742169	-0.390842
90	6	0	-7.530637	-1.965120	0.723347
91	1	0	-7.534862	-1.107950	1.407728
92	1	0	-8.531677	-2.100436	0.311593
93	1	0	-7.244768	-2.866197	1.279625
94	7	0	-5.416659	4.434735	-0.628832

95	7	0	5.923803	-3.490028	-1.216533
96	6	0	6.157236	-4.674949	-0.400514
97	1	0	5.297285	-5.356607	-0.417488
98	1	0	7.021573	-5.211232	-0.790244
99	1	0	6.363820	-4.412376	0.644639
100	6	0	-5.622274	5.218790	-1.839038
101	1	0	-6.581358	5.731633	-1.773083
102	1	0	-4.842193	5.979941	-1.958802
103	1	0	-5.629872	4.593725	-2.742803
104	6	0	-0.072148	-1.434848	4.902489
105	1	0	0.019403	-1.638401	5.965533
106	8	0	0.198445	-6.827272	1.077461
107	6	0	0.249339	-7.788795	0.029069
108	1	0	0.389727	-8.755996	0.513561
109	1	0	-0.682769	-7.806973	-0.548785
110	1	0	1.089037	-7.600770	-0.651120
111	1	0	1.273529	0.672835	-5.107578
112	1	0	1.488701	-1.072708	-5.239126
113	1	0	0.327525	-0.246441	-6.287982
114	1	0	-0.301140	2.006237	4.859819
115	1	0	-1.001239	0.979672	6.121610
116	1	0	0.750259	1.104283	5.962644

Transient absorption, emission, and steady-state photoluminescence spectra

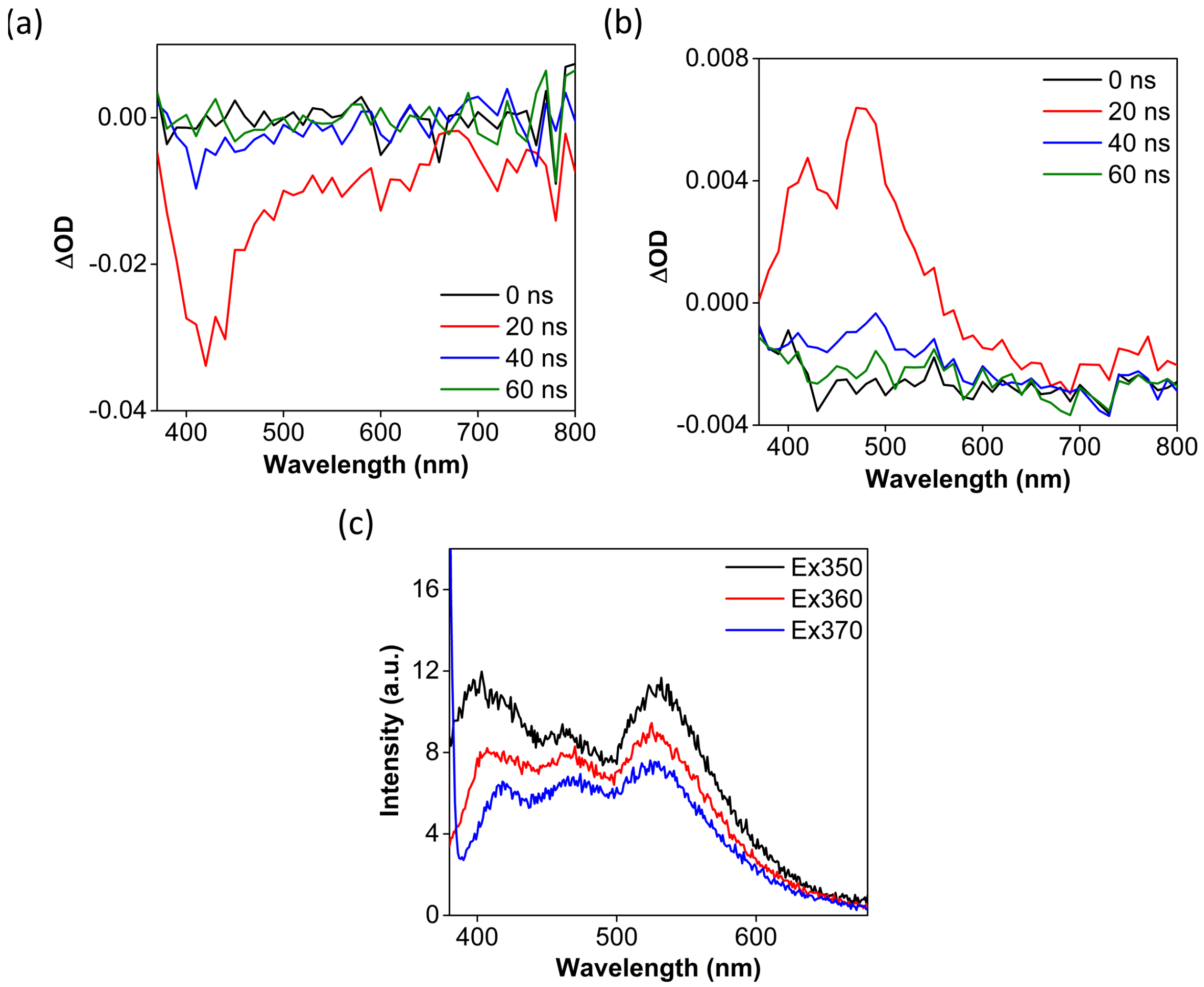

Supplementary Figure S28. Transient (a) absorption and (b) emission spectra of 0.050 mM solutions of **5** collected in MeCN at 298, after irradiation at 355 nm. (c) Steady-state photoluminescence spectra of a 0.050 mM solution of **5** collected in MeCN at 298 K.

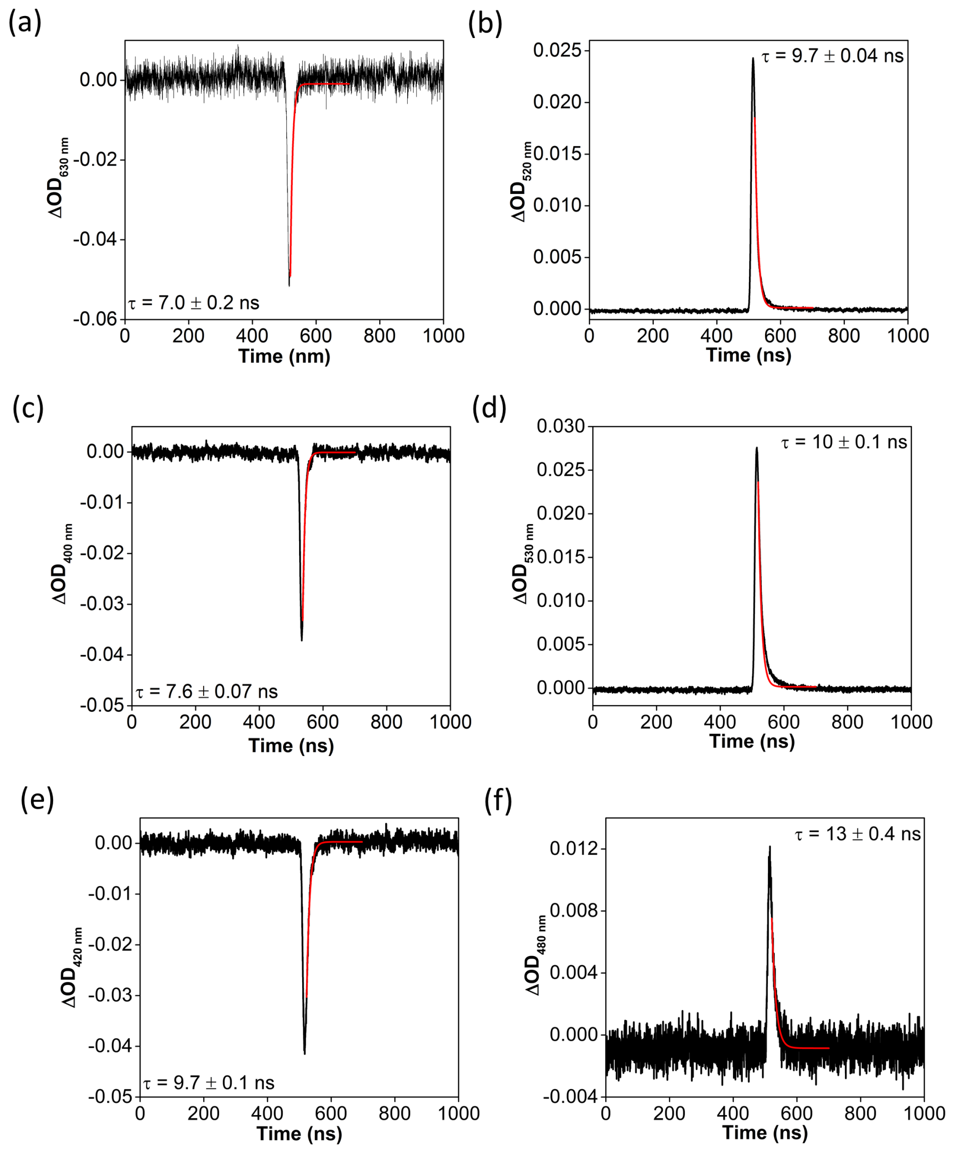

Supplementary Figure S29. Transient (a) absorption spectrum at 630 nm and (b) emission spectrum at 520 nm for 0.050 mM **4** in DCM. Transient (c) absorption spectrum at 400 nm and (d) emission spectrum at 530 nm for 0.050 mM **4** in MeCN. Transient (e) absorption spectrum at 420 nm and (f) emission spectrum at 480 nm for 0.050 mM **5** in MeCN. All samples were irradiated at 355 nm.

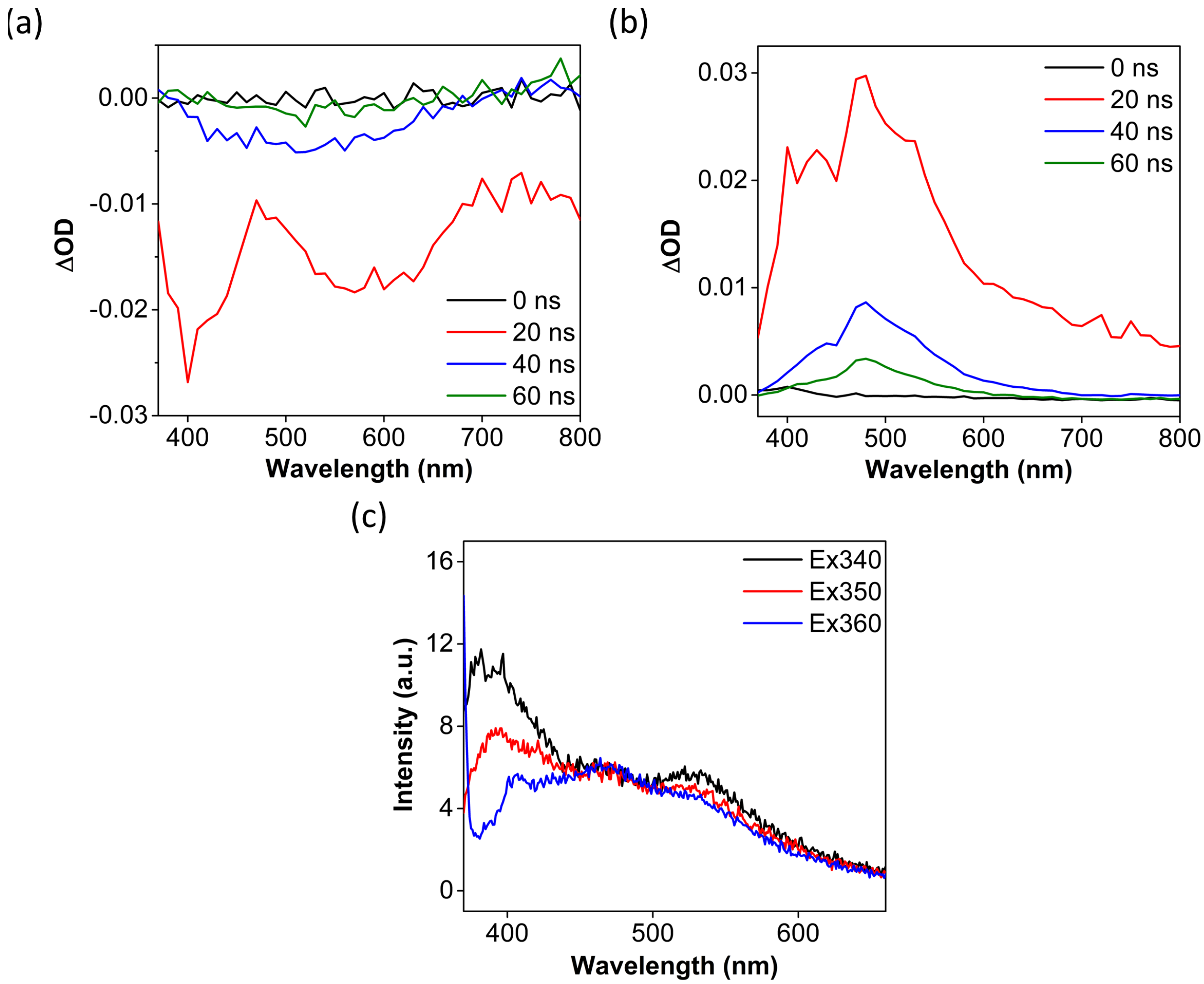

Supplementary Figure S30. Transient (a) absorption and (b) emission spectra of 0.050 mM solutions of **4** collected in MeCN at 298, after irradiation at 355 nm. (c) Steady-state photoluminescence spectra of a 0.050 mM solution of **4** collected in MeCN at 298 K.

References

1 Nonoyama, M. Benzo[*h*]quinolin-10-yl-*N* iridium (III) complexes. *Bull. Chem. Soc. Jpn.* **47**, 767-768 (1974).

2 Hasan, K. & Zysman-Colman, E. Synthesis, UV–vis and CV properties of a structurally related series of bis(arylimino)acenaphthenes (Ar-BIANs). *J. Phys. Org. Chem.* **26**, 274-279 (2013).

3 Ross, S. D., Finkelstein, M. & Petersen, R. C. Solvent effects in the reactions of *N*-bromosuccinimide with toluene, fluorene and acenapthene; evidence for a polar mechanism in propylene carbonate. *J. Am. Chem. Soc.* **80**, 4327-4330 (1958).

4 Watanabe, T., Tanaka, Y., Sekiya, K., Akita, Y. & Ohta, A. A convenient synthesis of methylamino and dimethylamino substituted aromatic compounds. *Synthesis* **1980**, 39-41 (1980).

5 Popp, T. A. & Bracher, F. N-methylation of aromatic amines and n-heterocycles under acidic conditions with the TTT (1,3,5-trioxane–triethylsilane–trifluoroacetic acid) system. *Synthesis* **47**, 3333-3338 (2015).

6 Hanaya, K., Muramatsu, T., Kudo, H. & Chow, Y. L. Reduction of aromatic nitro-compounds to amines with sodium borohydride-copper(II) acetylacetonate. *J. Chem. Soc., Perkin Trans. 1*, 2409-2410 (1979).

7 Sakai, N., Asama, S., Konakahara, T. & Ogiwara, Y. Indium(III)-catalyzed reduction of nitrobenzenes to anilines: Scope and limitations. *Synthesis* **47**, 3179-3185 (2015).

8 Hasan, K. *et al.* Tuning the emission of cationic iridium (III) complexes towards the red through methoxy substitution of the cyclometalating ligand. *Sci. Rep.* **5**, 12325 (2015).

9 Li, W. J. *et al.* An improved protocol for the preparation of 3-pyridyl- and some arylboronic acids. *J. Org. Chem.* **67**, 5394-5397 (2002).

10 Deng, J. Z. *et al.* Copper-facilitated suzuki reactions: Application to 2-heterocyclic boronates. *Org. Lett.* **11**, 345-347 (2008).

11 Sindhuja, E., Ramesh, R. & Liu, Y. Palladium(II) thiocarboxamide complexes: Synthesis, characterisation and application to catalytic suzuki coupling reactions. *Dalton Trans.* **41**, 5351-5361 (2012).

12 Sasaki, I., Vendier, L., Sournia-Saquet, A. & Lacroix, P. G. Facile synthesis of cyclometalated ruthenium complexes with substituted phenylpyridines. *Eur. J. Inorg. Chem.*, 3294-3302 (2006).

13 Lloyd, D. G. *et al.* Benzoxepin-derived estrogen receptor modulators: A novel molecular scaffold for the estrogen receptor. *J. Med. Chem.* **47**, 5612-5615 (2004).

14 Sasaki, I., Vendier, L., Sournia-Saquet, A. & Lacroix, P. G. Facile synthesis of cyclometalated ruthenium complexes with substituted phenylpyridines. *Eur. J. Inorg. Chem.* **2006**, 3294-3302 (2006).

15 Pavlishchuk, V. V. & Addison, A. W. Conversion constants for redox potentials measured versus different reference electrodes in acetonitrile solutions at 25 degrees C. *Inorg. Chim. Acta* **298**, 97-102 (2000).

16 Gaussian 09, Revision D.01, M. J. Frisch *et al.* Gaussian, Inc., Wallingford CT (2013).

17 Becke, A. D. Density functional thermochemistry. III. The role of exact exchange. *J. Chem. Phys.* **98**, 5648-5652 (1993).

18 Lee, C., Yang, W. & Parr, R. G. Development of the colle-salvetti correlation-energy formula into a functional of the electron density. *Phys. Rev. B* **37**, 785-789 (1988).

19 McLean, A. D. & Chandler, G. S. Contracted gaussian basis sets for molecular calculations. I. Second row atoms, *Z*=11–18. *J. Chem. Phys.* **72**, 5639-5648 (1980).

20 Binkley, J. S., Pople, J. A. & Hehre, W. J. Self-consistent molecular orbital methods. 21. Small split-valence basis sets for first-row elements. *J. Am. Chem. Soc.* **102**, 939-947 (1980).

21 Casida, M. E., Jamorski, C., Casida, K. C. & Salahub, D. R. Molecular excitation energies to high-lying bound states from time-dependent density-functional response theory: Characterisation and correction of the time-dependent local density approximation ionisation threshold. *J. Chem. Phys.* **108**, 4439-4449 (1998).

22 Stratmann, R. E., Scuseria, G. E. & Frisch, M. J. An efficient implementation of time-dependent density-functional theory for the calculation of excitation energies of large molecules. *J. Chem. Phys.* **109**, 8218-8224 (1998).

23 Cossi, M., Rega, N., Scalmani, G. & Barone, V. Energies, structures, and electronic properties of molecules in solution with the c-pcm solvation model. *J. Comput. Chem.* **24**, 669-681 (2003).

24 M. Cossi, V. B. Time-dependent density functional theory for molecules in liquid solutions. *J. Chem. Phys.* **115**, 4708-4717 (2001).

25 Barone, V. & Cossi, M. Quantum calculation of molecular energies and energy gradients in solution by a conductor solvent model. *J. Phys. Chem. A* **102**, 1995-2001 (1998).

26 Browne, W. R., O'Boyle, N. M., McGarvey, J. J. & Vos, J. G. Elucidating excited state electronic structure and intercomponent interactions in multicomponent and supramolecular systems. *Chem. Soc. Rev.* **34**, 641-663 (2005).

27 O'Boyle, N. M., Tenderholt, A. L. & Langner, K. M. GaussSum v2.2. *J. Comp. Chem.* 29, 839-845 (2008).

28 D. A. Zhurko, G. A. Z. *Chemcraft v1.5; Plimus: San Diego, ca.*, <http://www.chemcraftprog.com>
